# Supplementary material for: Randomised controlled trial to investigate the effectiveness of local oestrogen treatment in postmenopausal women undergoing pelvic organ prolapse surgery (LOTUS): a pilot study to assess feasibility of a large multicentre trial
Source: BMJ Open. 2020 Sep 10;10(9):e025141. doi: 10.1136/bmjopen-2018-025141 (PMC7485245; doi:10.1136/bmjopen-2018-025141)
Supplement: Author's manuscript [file bmjopen-2018-025141.draft_revisions.pdf]

BMJ Open

BMJ Open is committed to open peer review. As part of this commitment we make the peer review history of every article we publish publicly available.

When an article is published we post the peer reviewers' comments and the authors' responses online. We also post the versions of the paper that were used during peer review. These are the versions that the peer review comments apply to.

The versions of the paper that follow are the versions that were submitted during the peer review process. They are not the versions of record or the final published versions. They should not be cited or distributed as the published version of this manuscript.

BMJ Open is an open access journal and the full, final, typeset and author-corrected version of record of the manuscript is available on our site with no access controls, subscription charges or pay-per-view fees (http://bmjopen.bmj.com).

If you have any questions on BMJ Open's open peer review process please email info.bmjopen@bmj.com

BMJ Open

A Randomised Controlled Trial to investigate the effectiveness of Local Oestrogen Treatment in Postmenopausal Women Undergoing Pelvic Organ Prolapse Surgery (LOTUS) – a pilot study to assess feasibility of a large multi-centre trial

Journal:	*BMJ Open*
Manuscript ID	bmjopen-2018-025141
Article Type:	Research
Date Submitted by the Author:	01-Nov-2018

Complete List of Authors:	Verghese, Tina; University of Birmingham, Institute of Metabolism and System Research<br>Middleton, Lee; University of Birmingham, School of Health and Population Sciences<br>Cheed, Versha; University of Birmingham, Birmingham Clinical Trials Unit<br>Leighton, Lisa; University of Birmingham, Birmingham Clinical Trials Unit<br>Daniels, Jane; University of Nottingham, Nottingham Clinical Trials Unit; University of Nottingham Clinical Trials Unit<br>Latthe, Pallavi; Birmingham Women's and Children's NHS Foundation Trust, Obstetrics and Gynaecology

Keywords:	pilot, feasibility, pelvic organ prolapse, postmenopausal, recurrence, oestrogen

SCHOLARONE™
Manuscripts

I, the Submitting Author has the right to grant and does grant on behalf of all authors of the Work (as defined in the below author licence), an exclusive licence and/or a non-exclusive licence for contributions from authors who are: i) UK Crown employees; ii) where BMJ has agreed a CC-BY licence shall apply, and/or iii) in accordance with the terms applicable for US Federal Government officers or employees acting as part of their official duties; on a worldwide, perpetual, irrevocable, royalty-free basis to BMJ Publishing Group Ltd ("BMJ") its licensees and where the relevant Journal is co-owned by BMJ to the co-owners of the Journal, to publish the Work in this journal and any other BMJ products and to exploit all rights, as set out in our licence.

The Submitting Author accepts and understands that any supply made under these terms is made by BMJ to the Submitting Author unless you are acting as an employee on behalf of your employer or a postgraduate student of an affiliated institution which is paying any applicable article publishing charge ("APC") for Open Access articles. Where the Submitting Author wishes to make the Work available on an Open Access basis (and intends to pay the relevant APC), the terms of reuse of such Open Access shall be governed by a Creative Commons licence – details of these licences and which Creative Commons licence will apply to this Work are set out in our licence referred to above.

Other than as permitted in any relevant BMJ Author's Self Archiving Policies, I confirm this Work has not been accepted for publication elsewhere, is not being considered for publication elsewhere and does not duplicate material already published. I confirm all authors consent to publication of this Work and authorise the granting of this licence.

For peer review only

A Randomised Controlled Trial to investigate the effectiveness of Local Oestrogen Treatment in Postmenopausal Women Undergoing Pelvic Organ Prolapse Surgery (LOTUS) – a pilot study to assess feasibility of a large multi-centre trial

For peer review only

Tina Sara Verghese MS MRCOG, Clinical Research Fellow, Institute of Metabolism and System Research College of Medical & Dental Sciences, University of Birmingham, B15 2TT, UK; t.s.verghese@bham.ac.uk

Lee Jonathan Middleton, MSc, Senior Medical Statistician, Birmingham Clinical Trials Unit, University of Birmingham, B15 2TT, UK; l.j.middleton@bham.ac.uk

Versha Cheed, MSc, Medical Statistician, Birmingham Clinical Trials Unit, University of Birmingham, B15 2TT, UK; solankiv@adf.bham.ac.uk

Lisa Leighton LOTUS Trial Co-ordinator, Birmingham Clinical Trials Unit, University of Birmingham, B15 2TT, UK; leightlj@adf.bham.ac.uk

Jane P Daniels PhD MSc MMedSci BSc, Professor of Clinical Trials, Faculty of Medicine & Health Sciences, University of Nottingham, NG7 2UH,UK; jane.daniels@nottingham.ac.uk

Pallavi Manish Latthe MD FRCOG DFFP, Consultant Urogynaecologist, Birmingham Women's and Children's NHS Foundation Trust, B15 2TG, UK; pallavi.latthe@bwnft.nhs.uk

on behalf of the **LOTUS trial collaborative group**

Corresponding author:
E-mail: t.s.verghese@bham.ac.uk

Formatted for BMJ open
Abstract: 286
Main body: 3733

For peer review only - http://bmjopen.bmj.com/site/about/guidelines.xhtml

Abstract

Objective: To evaluate the feasibility of a multicentre randomised controlled trial comparing oestrogen treatment with no oestrogen supplementation in women undergoing pelvic organ prolapse (POP) surgery.

Design and setting: A randomised, parallel, open, external pilot trial involving six UK urogynaecology centres (July 2015- August 2016).

Participants: Post-menopausal women with POP opting for surgery, unless involving mesh or for recurrent POP in same compartment.

Intervention: Women were randomised (1:1) to pre and post-operative oestrogen or no treatment. Oestrogen treatment (oestradiol hemihydrate 10mcg vaginal pessaries) commenced 6 weeks prior to surgery (once daily for two weeks, twice weekly for four weeks) and twice weekly for 26 weeks from 6 weeks post-surgery.

Outcome measures: The main outcomes were assessment of eligibility and recruitment rates along with compliance and data completion. Prolapse specific quality of life measures included the Pelvic Floor Distress Inventory Short-Form 20 and the Patient Global Impression of Improvement, completed at 6 and 12 months. Clinical outcomes measured by POP Quantification system, at 6 months post-surgery and post-operative complications.

Results: 325 women seeking POP surgery were screened over 13 months and 157 (48%) were eligible. Of these, 100 (64%) were randomised, 50 to oestrogen and 50 to no oestrogen treatment, with 89 (44/45 respectively) ultimately having surgery. Of these, 89% (79/89) returned complete questionnaires at six months and 78% (32/41) reported good compliance with oestrogen. No serious adverse events were attributable to oestrogen use.

For peer review only - http://bmjopen.bmj.com/site/about/guidelines.xhtml

Conclusions: A large multicentre RCT of oestrogen versus no treatment is feasible as it is possible to randomise and follow up participants with high fidelity. Four predefined feasibility criteria were met. Compliance with treatment regimens is not a barrier. A larger trial is required to definitively address the role of peri-operative oestrogen supplementation.

Trial registration number: ISRCTN46661996.

Keywords

Pilot, feasibility, pelvic organ prolapse, postmenopausal, recurrence, oestrogen.

For peer review only

Article Summary

Strengths and limitations of this study

- This randomised, external, pilot trial had pre-planned feasibility thresholds to judge to assess whether a large trial is feasible.
- The data on number of women at each stage of the trial (screening, randomisation, using the intervention and completing outcome assessments at two follow-up time-points) were collected to estimate recruitment, retention and compliance rates
- There was the potential for post-randomisation exclusions of participants who were ultimately considered unfit for surgery
- Blinding of the oestrogen pessary was not possible, so reported outcomes could be subject to responder bias
- The trial was not designed to be large or long enough to provide convincing evidence for use of pre and post-operative oestrogen treatment

Trial funding

This work was funded by UK National Institute for Health Research, Research for Patient Benefit programme (grant reference PB-PG-0213-30126)

Declaration of interests

PML received sponsorship from Astellas, Contura and Pfizer to attend meetings.

All the other authors declare that they have no competing interests.

For peer review only - http://bmjopen.bmj.com/site/about/guidelines.xhtml

Introduction

Pelvic organ prolapse (POP) reportedly occurs in 40-60% of parous women(1). However the true incidence may be underreported, as many women feel embarrassed to seek medical advice. Women with symptomatic POP present with a sense of vaginal bulge, in association with a variety of urinary, bowel and sexual symptoms. In postmenopausal women lack of oestrogen is hypothesised to weaken the pelvic floor muscles(2). Whilst POP surgery is effective, with symptom improvement in 70-88% of women, the risk of recurrent prolapse is high, estimated at 10-30%(1). Efforts to reduce perioperative complications of POP surgery and enhancement of long-term cure are key priorities for the future management of a rapidly ageing population. In postmenopausal women with vaginal atrophy, surgical dissection may be difficult due to thinning of the vaginal walls. In addition, they may be at a higher risk of surgical wound infection, secondary to changes in the vaginal flora. Oestrogen treatment is often used, on its own or in conjunction with various treatment modalities, for vaginal atrophy(3) . However, despite frequent use, there is no robust data on the benefits of pre and postoperative oestrogen treatment in postmenopausal women undergoing POP surgery.

A Cochrane review by Ismail et al, published in 2010 did not find any clear evidence to suggest whether oestrogens for the prevention and treatment of POP and concluded an adequately powered RCT with long term follow up is needed to identify the benefits or risks associated with oestrogen supplementation, including before and after POP surgery(3).

There is a plausible argument for using low-dose oestrogen to improve the vaginal environment and reduce infections at the time of prolapse surgery, but there is little evidence to support its effect on the quality of surgical repair, prolapse cure rates or recurrence. Furthermore, there is no information about any effect in terms of prolapse related symptoms,

For peer review only - http://bmjopen.bmj.com/site/about/guidelines.xhtml

overall quality of life (QoL) and outcomes important to the women. Finally, the duration of oestrogen treatment, and cost-effectiveness compared with current practice are unknown.

There were over 21,000 pelvic floor repair surgeries performed in England in 2014 on women with an average age of 61 years and incurring a hospital stay of 2 days (HES data, Q08.9, P23.1). These data mask the impact of surgical complications on resource use and quality of life, which could be mitigated if perioperative oestrogen was found to be clinically and cost-effective. Moreover, if rates of recurrence rates of prolapse are also reduced as a result of a quality surgical repair, this may lead to substantial cost savings for health systems.

Feasibility studies serve to address the question "can this study be done", by assessing uncertainty in key trial parameters, whilst pilot studies are a small scale version of the definitive study design to test whether all components can work together. There is often overlap between the objectives of any preparatory studies, as the ultimate aim is to maximise the success of a definitive study by identifying problems with recruitment and data collection processes.

The overall aim of this research is to determine whether pre and postoperative local oestrogen treatment is more effective in improving prolapse related patient reported outcomes and reducing recurrence of prolapse symptoms when compared to no treatment. To answer this question a large, multi-centre RCT is required. In preparation, we conducted an external pilot study called Local Oestrogen Treatment in Postmenopausal Women Undergoing Pelvic Organ Prolapse Surgery (LOTUS). We aimed to assess the effectiveness of the patient identification and screening process, including reasons for failure to randomise eligible patients; evaluated the compliance with the treatment schedule; considered robustness and usability of data collection processes during and after the hospital episode and collected limited data on the proposed primary outcome measure and serious adverse events. We report this study as per the CONSORT checklist for reporting a pilot trial.

Methods

For peer review only - http://bmjopen.bmj.com/site/about/guidelines.xhtml

We conducted a randomised parallel group open external pilot trial of pre and post-operative oestrogen treatment versus no treatment in women undergoing POP surgery. A parallel qualitative evaluation of the barriers and facilitators to participation from a women's perspective was presented at International Continence Society Annual Meeting, Florence 2017. The study received a favourable ethical opinion from NHS Research Ethics Committee (NRES Committee West Midlands, REC number 15/WM/0092) and was registered with a primary clinical trials registry (ISRCTN 46661996).

Population

Eligible participants were postmenopausal women who were having POP surgery without use of vaginal mesh. Participants were excluded if they had any of the following: hormone replacement therapy in the last 12 months; previous breast/uterine malignancy or other hormone-dependant neoplasms; genital bleeding of unknown origin; previous thrombo-embolic episodes in relation to oestrogen; known allergy to oestrogens; two or more episodes of culture positive urinary tract infection (UTI) in the last 6 months; voiding dysfunction; previous POP surgery involving mesh or previous POP surgery in the same compartment.

Study conduct and randomisation

All patients thought to fulfil the eligibility criteria were approached with information regarding the trial. Participants were identified in urogynaecology clinics, provided with a patient information sheet and given the opportunity to consider participation.

Once eligibility was confirmed, the trial team, which comprised of urogynaecologists and research nurses conducted face to face sessions with the participants in order to obtain written informed consent and baseline data. Women who declined trial participation remained eligible to participate in the qualitative study. Randomisation was performed using a web-based central randomisation system (via Birmingham Clinical Trials Unit) to allocate patients to either oestrogen or no treatment in a 1:1 ratio. Minimisation was used to achieve balance between age (<65 years or ≥ 65 years), parity ($<=2$ or >2 vaginal births), maximum stage of prolapse (I, II or III/IV) and whether a concomitant continence surgery was planned. An

For peer review only - http://bmjopen.bmj.com/site/about/guidelines.xhtml

oversight committee was formed to provide independent guidance to the Trial Management Committee and to review accruing safety information during the period of recruitment.

Interventions

Those allocated oestrogen (oestradiol hemihydrate 10mcg vaginal pessaries; Vagifem™, Novo Nordisk) were instructed to use the oestrogen pessaries six weeks prior to surgery (once daily for two weeks and twice weekly for four weeks) up to the night before surgery. The treatment was restarted 6 weeks post-operatively, administering twice weekly for 20 weeks (4). Women were encouraged to insert the pessaries into the vagina at the same time of day. However, if a dose was missed patients were advised it should be administered as soon as possible thereafter, provided the next dose was not due. Participants allocated no treatment received the usual care of the randomising centre. The surgical approach to POP repair was at the discretion of the urogynaecological surgeon. It was not possible to blind clinicians or participants due to the nature of the interventions.

Clinical and patient-completed outcomes

Baseline data including clinical history and symptom-related questionnaires were recorded at the pre-surgery clinic appointment. Intraoperative, post-operative (six weeks) and six months post-surgery data including objective assessment of prolapse (POP quantification system [POP-Q]), complications (including urinary tract and vaginal infections) and repeat surgery information were collected by the randomising and/or operating clinician at scheduled appointments. The patient-reported questionnaires were: the Pelvic Floor Distress Inventory Short Form 20 (PFDI-SF20)(5), which comprises of three sub-domains (Pelvic Organ Prolapse Distress Inventory [POPDI-6], Colorectal-Anal Distress Inventory [CRADI-8], Urinary Distress Inventory [UDI-6]); the Pelvic Floor Impact Questionnaire (PFIQ-7), which also has three sub-domains (Pelvic Organ Prolapse Impact [POPIQ-7], Colorectal-Anal Impact [CRAIQ-7], Urinary Impact [UIQ-7]); the Pelvic Organ Prolapse/Urinary Incontinence Sexual Questionnaire (PISQ-12) (6) and Patient Global Impression of Improvement (PGI-I) (7). Questionnaires were collected at baseline then at six (at the

For peer review only - http://bmjopen.bmj.com/site/about/guidelines.xhtml

scheduled appointment) and twelve months, by postal questionnaire. Serious adverse events, reported by clinical staff, were collected throughout the duration of the study.

Sample size

We planned to randomise 100 women; this number would allow us to measure recruitment and compliance rates with 95% confidence interval (CI) width between 10 and 20%. It would also be enough women to estimate the standard deviation (SD) of POPDI-6 with reasonable confidence for future planning of a larger trial (95% CI for SD would be 7 points, assuming the SD is around 20).

Statistical analysis

Feasibility outcomes were considered with simple summary statistics, with uncertainty estimates provided by 95% confidence intervals. Clinical and patient reported outcome measures (PROMs) were analysed with point estimates (RR or mean difference) and 95% confidence intervals, adjusting for the minimisation variables. They were not subject to hypothesis testing as the size of the sample would not allow reliable assessment of the effect of the interventions. Participants were considered in the groups they were randomised to regardless of compliance in these summaries (intention-to-treat). The dataset used in this manuscript is available on request from Birmingham Clinical Trials Unit.

Pilot outcomes

The following outcomes and targets were set a-priori as being indicative that a larger trial would be feasible to conduct. These were: i) patient eligibility rate (the proportion of screened patients eligible) at least 25%; ii) patient recruitment rate (the proportion of eligible patients randomised) at least 25%; iii) compliance rate (the proportion of patients with good compliance to treatment, i.e. at least 75% allocated pessaries used): at least 50%; iv) data completion rate (number of follow-up questionnaires completed at six months): at least 75%.

For peer review only

For peer review only - http://bmjopen.bmj.com/site/about/guidelines.xhtml

Patient and Public involvement

Throughout the process of the development and the running of the feasibility study, the study team have actively involved participation from patients and public in order to refine the trial and make the study about postmenopausal women suffering from symptoms of pelvic organ prolapse (POP). The research team conducted a qualitative study to identify the motivations for, and barriers to recruitment and participation in LOTUS. Twenty women were interviewed which provided key insight into trial recruitment barriers. This application benefited from the qualitative study in several ways (e.g. approaching patients and the acceptability of the investigational medicinal product).

Focus groups interview were held through which the research team gained insight from women who were given the opportunity to express their views and felt that further research in this area is much needed

Results

Patients and follow-up

Recruitment took place over a 13-month period between July 2015 and August 2016 in six urogynaecology centres in the UK (Birmingham Women's Hospital, Croydon University Hospital, Basingstoke and North Hampshire Hospital, Walsall Manor Hospital, Royal Stoke University Hospital, James Cook University Hospital, Middlesbrough). Once the target sample size was reached, the study stopped randomising women.

Of the women who presented with prolapse and wanted surgery, 325 consecutive women were screened for eligibility, 157 were found to be eligible (48%, 95%CI: 40%-56%) and 100 (64%, 95% CI 56-72%) of those eligible were randomised (Figure 1). The minimisation algorithm provide appropriate balance for the minimisation factors; the average age of participants was 66 years with 47% having parity greater than two with an average BMI of

For peer review only - http://bmjopen.bmj.com/site/about/guidelines.xhtml

28.2. The maximum stage of prolapse was III or IV (increased severity) for 40% of participants (Table 1).

Of the 100 randomised women, 89 ultimately had surgery (details given in Table 2). Of the other 11, 5 could not have surgery due to health issues, 2 changed their mind about surgery, 2 did not attend their surgical appointment and 2 were diagnosed with cancer. Six month follow-up questionnaires were completed by 79/100 (79%) of participants. Of those that had surgery this equated to 79/89 (89%). Of those who had surgery, 100% had completed intraoperative data and 84/89 (94%) had post-operative data recorded at six weeks.

Compliance

Good compliance with oestrogen treatment (denoted as pessaries used at least >=75% of the expected time) was observed in 79% of participants pre-operatively (34/43), 83% at six weeks (35/42) and 71% at six months (20/28).

Clinical and patient-completed outcomes

Overall, scores from the PFDI-SF20 (POPDI-6 domain) were low at both 6 and 12 months, averaging 14.3 (SD: 16.4) and 15.6 (18.1) out of a maximum of 100, indicating a low level of prolapse-related symptoms at these times. Responses from the PFIQ-7 (POP-IQ-7 domain) were similarly low (Table 3). Scores appeared similar in both groups, but with expected high levels of uncertainty given the limited size of sample. The number of participants reporting being improved (very much better or better) on the PGI-I was 92% (73/79) at 6 months and 89% (70/79) at 12 months (Table 4). The number of participants with an objective rating of prolapse failure (from POP-Q) at 6 months was 22/55 (40%) (Table 5). Two repeat incontinence surgeries were recorded in the no treatment group over six months, with none in the oestrogen group.

Safety

For peer review only - http://bmjopen.bmj.com/site/about/guidelines.xhtml

More UTIs (8/42; 19% versus 4/42; 10%) - resulting in more antibiotics prescriptions (9/42; 21% versus 5/42; 12%) were reported in the 'no treatment' group than in the oestrogen group. Two serious adverse events were recorded in the oestrogen group - one woman with high temperature was admitted for intravenous antibiotics but culture of vaginal and urine samples were negative, and another woman was diagnosed with leukaemia. Neither were thought to be related to treatment. Two incidental hospitalisations were also recorded in the 'no treatment' group (one case of heavy bleeding thought to be unrelated to surgery and another with pancreatic cancer). No concerns were expressed by the independent oversight committee who met half way through the recruitment period to review the safety data.

Discussion

In this open label randomised study we sought to examine the feasibility of randomising post-menopausal women undergoing POP surgery to receive vaginal oestrogen. Our four key feasibility indicators were met and we have shown that a large multicentre randomised controlled trial (RCT) is feasible. It is possible to randomise and follow up patients with high fidelity over at least 12 months. Oestrogen treatment in the form of vaginal pessaries was well tolerated and consistently applied by most women. As this was a feasibility study with an open-label design, no inferences can be made about the treatment's therapeutic efficacy. However, the data does suggest that further research is warranted. There was evidence that the majority of women in both trial groups did report improvement in their prolapse symptoms, quality of life and other aspects of urinary and sexual function. The effect was sustained for at least the first six months after surgery irrespective of oestrogen treatment.

Among women planning to undergo surgical repair for prolapse, three studies have reported on preoperative vaginal oestrogen by various methods, compared with placebo or no treatment, with a total of 111 participants (8-10). Follow up varied from 12 weeks to three years of postoperative surveillance. No vaginal oestrogen was given following surgery. The

For peer review only - http://bmjopen.bmj.com/site/about/guidelines.xhtml

overall quality of evidence was poor. Use of vaginal oestrogen improved the vaginal maturation index, a histological measure of the status of the vaginal epithelium, at the time of surgery(11) and increased vaginal epithelial thickness(9) . Vaginal oestrogen application for six weeks preoperatively increased synthesis of mature collagen and increased thickness of the vaginal wall, suggesting this intervention improves both the substrate for suture placement at the time of repair and maintenance of tissue integrity of the pelvic floor(10) . In our study, we did not monitor the vaginal maturation index but we did note that there were fewer UTIs observed in the group that received vaginal oestrogen pre-operatively although the study was not powered for this outcome. The clinicians reported easier planes of dissection in women in the intervention group.

Mikkelsen et al described the three-year postoperative outcomes after preoperative treatment with vaginal oestradiol or placebo tablets before POP repair(9). They included a questionnaire of patient satisfaction, but no standardised or validated metric of prolapse symptoms, discontinuation rates and reasons were reported (8). They suggested that if vaginal oestrogen does improve tissue integrity then therapy may need to be continued postoperatively until the time of complete scar maturation (10). There is no clear evidence to state how long when a women would have adequate scar maturation after a prolapse surgery as this could be dependent on various factors of wound healing. In the LOTUS study women in the intervention group continued oestrogen up to 20 weeks post- surgery. The overall improvement in symptoms was comparable at 6 and 12 months post surgery in the groups. The majority of participants undergoing prolapse surgery in both groups demonstrated improvements in prolapse symptoms (90%) at 12 months, indicating that the. The benefit of vaginal oestrogen may be marginal, with the majority of improvement in patient symptoms pertaining to surgery. This is hypothetical as there has been no RCT with long term follow up on these patients randomised to postoperative local oestrogen vs placebo or no treatment.

Surgical failures requiring repeat POP repairs on an average occur within 2 years after

For peer review only - http://bmjopen.bmj.com/site/about/guidelines.xhtml

primary surgery. The PROSPECT study reported that 2% (6/395 standard repair) repeat surgeries were required as early as 1 year after primary surgery and 5% (16/348 standard repair) repeat surgery within 2 years(12). The authors concluded that follow up of patients for a minimum of 5 years would be required to assess recurrence of symptoms following POP surgery(12). We need to consider the pros and cons of long term maintenance treatment with vaginal oestrogen in the intervention group in a definitive study including costs, compliance, plateauing of benefits and regression of changes on stopping the treatment. The potential outcomes measured in long-term studies may include recurrence rates, interval of recurrence of prolapse symptoms from index surgery, reduction in urinary tract symptoms and sustained quality of life improvements and cost effectiveness analysis.

Strengths

This feasibility study was conducted over a period of 13 months during which the trial achieved its target recruitment of 100 women from 6 centres in the UK. The study efficiently met all their target criteria within the given time frame and justified that a substantial trial will be achievable.

We were able to capture patient responses by using validated PROMs such as the PFDI - SF20, PFIQ7 and PSIQ 12. The feasibility study has been reported in line with the CONSORT reporting guideline for feasibility/ pilot studies. The feasibility study has allowed the trial team to identify areas in the study process that require fine-tuning ahead of a larger trial. We are considering long term follow up of participants for a minimum of 5 years in order to capture the recurrence rates and relapse of POP symptoms after index surgery. In addition, we are considering offering participants in the intervention group to continue with long-term maintenance therapy with vaginal oestrogen.

Limitations

For peer review only - http://bmjopen.bmj.com/site/about/guidelines.xhtml

Ideally we would have liked to conduct a RCT comparing vaginal oestrogen with a placebo pessary. All of the symptom related outcome measures are patient-completed and potentially prone to reporting bias if the participant is aware of the treatment allocation. However, despite extensive discussions with clinical trial suppliers, we were unable to procure a placebo without it being prohibitively expensive. Oestrogen pessaries are supplied in individually packaged, single-use plastic applicators, which cannot be replicated with a placebo due to the trademark on the packaging. Disassembling the pessary from the applicator to repackage in an unbranded applicator and container would require additional stability testing to confirm the bioavailability of the oestradiol hemihydrate. The manufacturer of Vagifem, Novo Nordisk, declined to provide assistance. Oestradiol is also available as a cream, and a placebo cream could be procured, but the acceptability and adherence to a cream as the route of treatment was not evaluated in this pilot and we should not extrapolate adherence based on pessaries.

A few patients post randomisation could not undergo surgery as they were deemed to be unfit. To avoid this from happening, the patients could have undergone pre-operative assessments to determine surgical and anaesthetic risk and randomised once they were scheduled for surgery.We could potentially have sent telephone, email or text reminders to patient to improve compliance with the post operative pessary use but it would not only be expensive but also not reflect a real life situation.

Conclusion

At this stage, we cannot expect that the outcomes of this pilot study to be directly translated into clinical care as the study was not large enough to be able to detect small to moderate realistic-sized difference in rates of prolapse related QoL, nor was the scope wide enough in terms of the number of centres involved to return a generalisable result. However, this study is an important precursor to a larger, substantive trial and provides invaluable information that

For peer review only - http://bmjopen.bmj.com/site/about/guidelines.xhtml

will help to ensure its success, which ultimately is needed to provide a definitive answer this important question.

Funding

The study was funded by a grant from the National Institute for Health Research for Patient Benefit (grant number: PB-PG-0213-30126). The funder had no role in study design, data collection, interpretation or analysis, or in writing the report for publication.

Acknowledgements

We thank the collaborators, women who recruited into this study, the healthcare professionals at the recruitment sites and the funders.

Miss Ranee Thakar, Croydon

Mrs. Preeti Jain, Walsall

Mr. Christian Phillips, Basingstoke

Mr. Jason Cooper, North Staffordshire

Mr. Athele Khunda, South Tees

Prof Jane Daniels and Lisa Leighton

Patient and Public involvement:

Rev. Ann Simons and Dr. Mary Evans

Trial Oversight committee:

Prof Shakila Thangaratinam, Valerie Morton, Tom Maishman and Deepali Sinha.

Declaration of interests

PML received sponsorship from Astellas, Contura and Pfizer to attend meetings.

All the other authors declare that they have no competing interests.

Sponsorship

For peer review only - http://bmjopen.bmj.com/site/about/guidelines.xhtml

The study sponsor was the Birmingham Women's and Children's NHS Foundation Trust. The sponsor had no role in study design, data collection, interpretation or analysis, or in writing the report for publication.

Authors' contributions

LJM, JPD and PML conceptualised the study as co- applicants. LM, JPD and PML contributed to the intervention developmentand design PML oversaw the running of the trial and all the authors contributed to the on-going management of the trial. The LOTUS trial collaborative group collected data for the trial. LJM and VC developed the data analysis plan and summarised the results. TSV and LL evaluated the qualitative data. The manuscript was drafted by TSV with contributions from PML, LJM, JPD, VC and LL. All the authors contributed to the interpretation of the output and revised and reviewed the paper; they are the guarantors. All authors contributed to the writing of the final manuscript and provided critical comments during revisions. All authors approved the final version prior to submission. The Birmingham Clinical Trials Unit undertook the randomisation and data management and monitoring. The authors had full access to all the data from the study. The authors vouch for the accuracy and completeness of the data and analyses. All authors read and approved the final manuscript.

For peer review only

For peer review only - http://bmjopen.bmj.com/site/about/guidelines.xhtml

Table 1: Baseline Characteristics

Characteristic		Oestrogen (n=50)	No Treatment (n=50)	Overall (n=100)
Age, years[1]	<65	21 (42%)	21 (42%)	42 (42%)
	>=65	29 (58%)	29 (58%)	58 (58%)
	Mean (SD)	65.7 (8.2)	65.9 (8.4)	65.8 (8.3)
Ethnic Group	White	45 (90%)	43 (86%)	88 (88%)
	Asian	2 (4%)	3 (6%)	5 (5%)
	Black	3 (6%)	1 (2%)	4 (4%)
	Mixed	0 (-)	2 (4%)	2 (5%)
	Other ethnic group	0 (-)	1 (2%)	1 (1%)
BMI (mg/k^2)	Mean (SD)	28.1 (5.1)	28.2 (5.9)	28.2 (5.5)
	Missing (N)	2	7	9
Parity[1]	<=2	27 (54%)	26 (52%)	53 (53%)
	>2	23 (46%)	24 (48%)	47 (47%)
Maximum Stage of Prolapse[1]	I	7 (14%)	7 (14%)	14 (14%)
	II	23 (46%)	23 (46%)	46 (46%)
	III/IV	20 (40%)	20 (40%)	40 (40%)
Concomitant continence surgery performed[1]	N (%)	2 (4%)	3 (6%)	5 (5%)
Previous operation for prolapse	N (%)	4 (8%)	4 (8%)	8 (8%)
Anterior	No. of repairs Median (IQR)	1 (1 - 1)	1 (1 - 1)	1 (1 - 1)
Posterior	No. of repairs Median (IQR)	1 (1 - 1)	1 (1 - 1)	1 (1 - 1)
Hysterectomy	N (%)	12 (24%)	8 (16%)	20 (20%)
Vaginal pessary/ring currently in place	N (%)	5 (10%)	8 (16%)	13 (13%)
Physiotherapy treatment for prolapse/urinary incontinence in last 12 months	N (%)	8 (16%)	10 (20%)	18 (18%)
Drug treatment for urinary incontinence	N (%)	0 (-)	4 (8%)	4 (4%)
Treatment for overactive bladder	N (%)	0 (-)	0 (-)	0 (-)

[1] minimisation variable

For peer review only

Table 2: Operative details

Aspect of surgery		Oestrogen (n=44)	No Treatment (n=45)
POP surgery performed	Anterior repair	35 (80%)	34 (76%)
	Posterior repair	16 (36%)	19 (42%)
	Vaginal hysterectomy +/- bilateral salping-oopherectomy	26 (59%)	25 (56%)
	Sacrospinous Fixation	6 (14%)	6 (13%)
Concomitant surgery	Sling/Tension-free vaginal tape	1 (2%)	1 (2%)
	Botulinum toxin A	1 (2%)	1 (2%)
	Suprapublic catheter	1 (2%)	1 (2%)
Ease of dissection	Very easy	6 (14%)	5 (11%)
	Easy	22 (50%)	13 (29%)
	Normal	14 (%)	21 (47%)
	Difficult	2 (5%)	5 (11%)
	Very difficult	0 (-)	1 (2%)
Blood loss (g)	N; Median (IQR)	N=44<br>63 (50 - 125)	N=44<br>54 (50 - 100)
	Missing	0 (-)	1 (2%)
Visceral injury	Bladder injury	0 (-)	0 (-)
	Urethral injury	0 (-)	0 (-)
	Ureteric injury	1 (2%)	0 (-)
	Bowel injury	0 (-)	0 (-)
	Button holing of vagina	3 (7%)	1 (2%)
Suture used – Fascial plication[1]	Polyglactin (Vicryl®, Ethicon/Polysorb®, Medtronic)	10 (23%)	10 (22%)
	Polydioxanone (PDS®-II, Ethicon)	35 (80%)	34 (76%)
	Poligelcarprine (Monocryl®, Ethicon)	0 (-)	0 (-)
	Missing	0	1
Suture used – Vagina[1]	Polyglactin (Vicryl®, Ethicon/Polysorb®, Medtronic)	41 (93%)	42 (93%)
	Polydioxanone (PDS®-II, Ethicon)	5 (11%)	6 (13%)
	Poligelcarprine (Monocryl®, Ethicon)	3 (7%)	1 (2%)
	Vaginal packing used	23 (52%)	28 (62%)
Cather inserted in theatre	Yes	43 (98%)	42 (93%)
Post-operative complications noted before discharge	Infection	1 (2%)	3 (7%)
	Ureteric injury	0 (-)	0 (-)
	Bladder injury	0 (-)	0 (-)
	Bowel injury	0 (-)	0 (-)
	Vascular injury	0 (-)	0 (-)
	Neurological injury	0 (-)	0 (-)
	Peri or post-operative blood transfusion	0 (-)	0 (-)
	Peri or post-operative blood thromboembolism	0 (-)	0 (-)
	Death	0 (-)	0 (-)

For peer review only - http://bmjopen.bmj.com/site/about/guidelines.xhtml

[1]More than one can be selected, hence why numbers do not add up to total.

For peer review only

For peer review only - http://bmjopen.bmj.com/site/about/guidelines.xhtml

Table 3: Results of patient reported outcomes – PFDI20, PFIQ-7 and PISQ-12

Outcome measure Timepoint	Oestrogen N; Mean (SD)	No Treatment N; Mean (SD)	Difference between groups (95% CI)[3]
PFDI-SF20; POPDI-6 Domain (0-100, higher=worse pain)[1]			
Baseline	N=48; 43.4 (24.8)	N=49; 46.0 (24.4)	
6 Month	N=38; 16.3 (20.3)	N=41; 12.5 (11.6)	4.1 (-3.9, 12.1)
12 Month	N=37 16.7 (20.6)	N=41 14.6 (15.6)	4.4 (-4.7, 13.5)
PFDI-SF20; CRAD-8 Domain (0-100, higher=worse pain)[1]			
Baseline	N=48; 26.0 (25.0)	N=48; 24.3 (18.0)	
6 Month	N=38; 12.7 (14.4)	N=41; 15.2 (13.2)	-2.4 (-7.6, 3.4)
12 Month	N=37 12.8 (14.8)	N=42 15.8 (15.8)	-1.7 (-8.0, 4.6)
PFDI-SF20; UDI-6 Domain (0-100, higher=worse pain)[1]			
Baseline	N=48; 34.8 (22.6)	N=49; 37.9 (26.7)	
6 Month	N=38; 16.4 (17.5)	N=41; 17.2 (18.5)	0.5 (-8.9, 7.9)
12 Month	N=36 20.3 (17.5)	N=42 21.5 (25.3)	0.7 (-9.1, 10.4)
PFDI-SF20; Summary Score (0-300, higher=worse pain)[1]			
Baseline	N=48; 104.3 (62.8)	N=48; 107.0 (57.4)	
6 Month	N=38; 45.4 (43.8)	N=41; 45.0 (37.1)	0.6 (-18.4, 17.2)
12 Month	N=36 49.8 (44.1)	N=41 51.1 (47.7)	4.8 (-16.2, 25.8)
PFIQ-7; UIQ-7 Domain (0-100, higher=worse condition)[1]			
Baseline	N=50; 21.0 (25.8)	N=50; 21.5 (24.6)	
6 Month	N=38; 9.6 (21.7)	N=41; 5.9 (11.2)	1.3 (-6.9, 9.5)
12 Month	N=37; 8.9 (16.8)	N=42; 8.0 (13.9)	1.4 (-6.0, 8.8)
PFIQ-7; CRAIQ-7 Domain (0-100, higher=worse condition)[1]			
Baseline	N=50; 12.0 (21.0)	N=50; 8.7 (13.5)	
6 Month	N=38; 4.9 (13.1)	N=41; 2.4 (6.1)	1.6 (-2.5, 5.8)
12 Month	N=36; 4.5 (16.3)	N=42; 2.9 (6.1)	2.2 (-2.8, 7.1)
PFIQ-7; POPIQ-7 Domain (0-100, higher=worse condition)[1]			
Baseline	N=50;	N=50;	

For peer review only - http://bmjopen.bmj.com/site/about/guidelines.xhtml

	17.7 (21.2)	17.1 (22.1)	
6 Month	N=38;<br>5.0 (13.4)	N=41;<br>2.3 (6.1)	2.5 (-2.5, 7.5)
12 Month	N=36;<br>1.9 (6.8)	N=42;<br>1.7 (5.2)	0.2 (-2.7, 3.1)
***PFIQ-7; Summary Score (0-300, higher=worse pain)*[1]**			
Baseline	N=50;<br>50.8 (54.8)	N=50;<br>47.3 (51.9)	
6 Month	N=38;<br>19.5 (39.8)	N=41;<br>10.7 (19.8)	6.2 (-8.7, 21.2)
12 Month	N=36;<br>14.3 (29.1)	N=42;<br>12.7 (18.3)	4.5 (-6.8, 15.8)
***PISQ-12 (0-48, higher=better sexual function)*[2]**			
Baseline	N=18;<br>31.2 (6.5)	N=19;<br>33.1 (7.3)	
6 Month	N=11;<br>33.1 (4.1)	N=14;<br>36.1 (5.7)	-2.4 (-7.0, 2.2)
12 Month	N=11;<br>34.5 (2.7)	N=10;<br>32.8 (5.1)	3.4 (0.6, 6.2)

[1]<0 indicates less pain/better condition failures with oestrogen

[2]>0 indicates better with oestrogen

[3]Differences are adjusted for baseline score and minimisation variables

For peer review only

Table 4: Results of patient reported outcomes – patient global impression of improvement (PGI-I)

Timepoint *PGI-I response*		Oestrogen	No Treatment	Relative risk (95%CI)[3]
**6 Week**		N=42	N=42	
1 = Very much better	N (%)	22 (52%)	14 (33%)	
2 = Much better	N (%)	16 (38%)	18 (43%)	
3 = A little better	N (%)	4 (10%)	8 (19%)	
4 = No change	N (%)	0 (-)	2 (5%)	
5 = A little worse	N (%)	0 (-)	0 (-)	
6 = Much Worse	N (%)	0 (-)	0 (-)	
7 = Very Much Worse	N (%)	0 (-)	0 (-)	
Better score[1]	N (%)	42 (100%)	40 (95%)	1.05 (0.98 – 1.12)
Worse score[2]	N (%)	0 (-)	2 (5%)	
**6 Month**		N=38	N=41	
1 = Very much better	N (%)	13 (34%)	18 (44%)	
2 = Much better	N (%)	16 (42%)	17 (41%)	
3 = A little better	N (%)	4 (11%)	5 (12%)	
4 = No change	N (%)	3 (8%)	0 (-)	
5 = A little worse	N (%)	2 (5%)	1 (2%)	
6 = Much Worse	N (%)	0 (-)	0 (-)	
7 = Very Much Worse	N (%)	0 (-)	0 (-)	
Better score[1]	N (%)	33 (87%)	40 (98%)	0.89 (0.78 – 1.02)
Worse score[2]	N (%)	5 (13%)	1 (2%)	
**12 Month**		N=37	N=42	
1 = Very much better	N (%)	14 (38%)	17 (40%)	
2 = Much better	N (%)	15 (41%)	17 (40%)	
3 = A little better	N (%)	3 (8%)	4 (10%)	
4 = No change	N (%)	4 (11%)	2 (5%)	
5 = A little worse	N (%)	1 (3%)	0 (-)	
6 = Much Worse	N (%)	0 (-)	2 (5%)	
7 = Very Much Worse	N (%)	0 (-)	0 (-)	
Better score[1]	N (%)	32 (86%)	38 (90%)	0.96 (0.81 – 1.12)
Worse score[2]	N (%)	5 (14%)	4 (10%)	

[1]Better score is derived from scores 1 (Very much better) to 3 (A little better)
[2]Worse score is derived from scores 4 (No change) to 7 (Very much worse)
[3]>1 indicates better with oestrogen

Table 5: Results of POP-Q assessment

Anatomical site in vagina Timepoint	Oestrogen N; Mean (SD)	No Treatment N; Mean (SD)	Difference between groups (95% CI)
Aa (anterior wall), cm			
Baseline	N=49; 0.1 (1.9)	N=48; -0.3 (1.7)	
6 Months[1]	N=26; -1.7 (2.0)	N=29; -2.6 (2.0)	0.7 (0.1, 1.3)
Ba (anterior edge), cm			
Baseline	N=49; 0.2 (2.0)	N=48; 0.1 (1.8)	
6 Months[1]	N=25; -2.0 (2.0)	N=29; -2.9 (1.9)	0.6 (0.0, 1.2)
C (cervix/cuff), cm			
Baseline	N=49; -3.7 (2.5)	N=48; -3.4 (3.4)	
6 Months[1]	N=25; -2.9 (2.8)	N=29; -2.2 (3.6)	-0.2 (-1.3, 0.9)
Ap (posterior wall), cm			
Baseline	N=49; -1.2 (1.5)	N=48; -1.0 (1.7)	
6 Months[1]	N=26; -0.8 (1.7)	N=29; -1.0 (1.7)	0.5 (-0.2, 1.1)
Bp (posterior edge), cm			
Baseline	N=49; -1.1 (1.6)	N=48; -1.0 (1.8)	
6 Months[1]	N=26; -0.9 (1.8)	N=29; -1.0 (1.8)	0.4 (-0.3, 1.1)
D (posterior fornix), cm			
Baseline	N=39; -5.3 (1.9)	N=40; -5.1 (2.6)	
6 Months[1]	N=7; -1.3 (1.5)	N=4; -1.8 (1.9)	0.7 (-6.2, 7.5)
Total vaginal length, cm			
Baseline	N=49; 8.5 (1.1)	N=48; 8.0 (1.6)	
6 Months[2]	N=26; -0.2 (1.3)	N=29; -0.5 (1.7)	0.3 (0.4, 0.9)
Objective prolapse (any of Aa, Ba, C, Ap, Bp, or D>0) at six months			
	**N (%)**	**N (%)**	**Relative risk (95%CI)[3]**
Failure	9/26	13/29	0.77 (0.40 – 1.50)

[1]Change from baseline adjusted for baseline; difference <0 indicates better with oestrogen

[2]Adjusted for baseline length; difference<0 indicates better with oestrogen

[3]<1 indicates less prolapse failures with oestrogen

For peer review only - http://bmjopen.bmj.com/site/about/guidelines.xhtml

References

1. Maher C, Feiner B, Baessler K, Christmann-Schmid C, Haya N, Brown J. Surgery for women with anterior compartment prolapse. The Cochrane database of systematic reviews. 2016;11:CD004014.
2. Nilsson K, Risberg B, Heimer G. The vaginal epithelium in the postmenopause--cytology, histology and pH as methods of assessment. Maturitas. 1995;21(1):51-6.
3. Ismail SI, Bain C, Hagen S. Oestrogens for treatment or prevention of pelvic organ prolapse in postmenopausal women. The Cochrane database of systematic reviews. 2010(9):CD007063.
4. [Available from: https://www.birmingham.ac.uk/Documents/college-mds/trials/bctu/lotus/LOTUS-protocol-V2.0.pdf

.
5. Barber MD WM BR. Short forms of two condition-specific quality-of-life questionnaires for women with pelvic floor disorders (PFDI-20 and PFIQ-7). . Am J Obstet Gynecol

2005.
6. Rogers RG, Coates KW, Kammerer-Doak D, Khalsa S, Qualls C. A short form of the Pelvic Organ Prolapse/Urinary Incontinence Sexual Questionnaire (PISQ-12). Int Urogynecol J Pelvic Floor Dysfunct. 2003;14(3):164-8; discussion 8.
7. Srikrishna S, Robinson D, Cardozo L. Validation of the Patient Global Impression of Improvement (PGI-I) for urogenital prolapse. Int Urogynecol J. 2010;21(5):523-8.
8. Felding C, Mikkelsen AL, Clausen HV, Loft A, Larsen LG. Preoperative treatment with oestradiol in women scheduled for vaginal operation for genital prolapse. A randomised, double-blind trial. Maturitas. 1992;15(3):241-9.
9. Mikkelsen AL, Felding C, Clausen HV. Clinical effects of preoperative oestradiol treatment before vaginal repair operation. A double-blind, randomized trial. Gynecol Obstet Invest. 1995;40(2):125-8.
10. Vaccaro CM, Mutema GK, Fellner AN, Crisp CC, Estanol MV, Kleeman SD, et al. Histologic and cytologic effects of vaginal estrogen in women with pelvic organ prolapse: a randomized controlled trial. Female Pelvic Med Reconstr Surg. 2013;19(1):34-9.
11. Moalli PA, Shand SH, Zyczynski HM, Gordy SC, Meyn LA. Remodeling of vaginal connective tissue in patients with prolapse. Obstet Gynecol. 2005;106(5 Pt 1):953-63.
12. Glazener CM, Breeman S, Elders A, Hemming C, Cooper KG, Freeman RM, et al. Mesh, graft, or standard repair for women having primary transvaginal anterior or posterior compartment prolapse surgery: two parallel-group, multicentre, randomised, controlled trials (PROSPECT). Lancet. 2017;389(10067):381-92.

For peer review only - http://bmjopen.bmj.com/site/about/guidelines.xhtml

Figure 1: Flow of participants through the trial

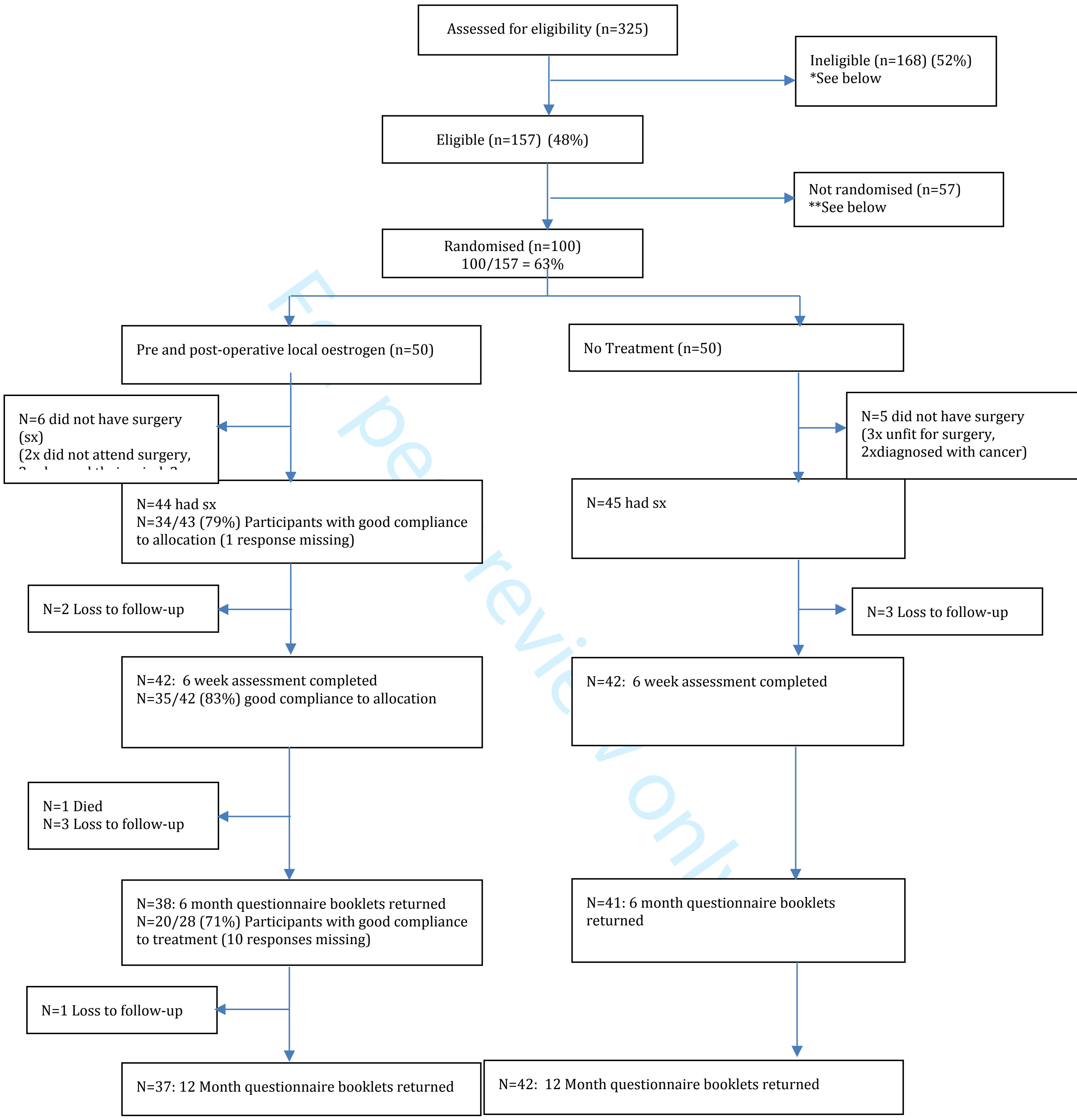

*** Reasons for Ineligibility**

Reason	N=168 (%)
No prolapse surgery required	38 (22)
Recurrence of prolapse in the same compartment	32 (19)

For peer review only - http://bmjopen.bmj.com/site/about/guidelines.xhtml

Has had previous breast or uterine malignancy	20 (12)
Undergoing HRT treatment	17 (10)
Unable to understand English	16 (9)
Genital bleeding of unknown origin	15 (9)
Has had previous hormone-dependent neoplasms	13 (8)
Previous POP surgery involving mesh	6 (3)
Previous thrombo-embolic episode related to oestrogen therapy	4 (2)
Two or more culture positive UTI episodes in the last 6 months	1 (<1)
*Other	6 (3)

**Reasons for other:*
Comorbidities
Wants surgery but at a much later date
Needed further bowel surgery

****Reasons for Non Randomisation**

Reasons	N=57 (%)
Patient did not attend clinic	19 (33)
Not enough time to discuss trial and gain informed consent	14 (25)
Declined Consent/not willing to be randomised	9 (16)
Wants surgical management involving mesh	5 (9)
Not willing to wait 6 weeks for surgery	5 (9)
Unable to gain consent	4 (7)
Did not want to complete questionnaires	1 (2)

For peer review only

CONSORT 2010 checklist of information to include when reporting a randomised trial*

Section/Topic	Item No	Checklist item	Reported on page No
**Title and abstract**			
	1a	Identification as a randomised trial in the title	1
	1b	Structured summary of trial design, methods, results, and conclusions (for specific guidance see CONSORT for abstracts)	2
**Introduction**			
Background and objectives	2a	Scientific background and explanation of rationale	5
	2b	Specific objectives or hypotheses	5
**Methods**			
Trial design	3a	Description of trial design (such as parallel, factorial) including allocation ratio	6
	3b	Important changes to methods after trial commencement (such as eligibility criteria), with reasons	7
Participants	4a	Eligibility criteria for participants	7
	4b	Settings and locations where the data were collected	7
Interventions	5	The interventions for each group with sufficient details to allow replication, including how and when they were actually administered	8
Outcomes	6a	Completely defined pre-specified primary and secondary outcome measures, including how and when they were assessed	8
	6b	Any changes to trial outcomes after the trial commenced, with reasons	8
Sample size	7a	How sample size was determined	9
	7b	When applicable, explanation of any interim analyses and stopping guidelines	9
Randomisation:			
Sequence generation	8a	Method used to generate the random allocation sequence	7
	8b	Type of randomisation; details of any restriction (such as blocking and block size)	7
Allocation concealment mechanism	9	Mechanism used to implement the random allocation sequence (such as sequentially numbered containers), describing any steps taken to conceal the sequence until interventions were assigned	7
Implementation	10	Who generated the random allocation sequence, who enrolled participants, and who assigned participants to interventions	7
Blinding	11a	If done, who was blinded after assignment to interventions (for example, participants, care providers, those	-

For peer review only

		assessing outcomes) and how	
	11b	If relevant, description of the similarity of interventions	-
Statistical methods	12a	Statistical methods used to compare groups for primary and secondary outcomes	9
	12b	Methods for additional analyses, such as subgroup analyses and adjusted analyses	9
**Results**			
Participant flow (a diagram is strongly recommended)	13a	For each group, the numbers of participants who were randomly assigned, received intended treatment, and were analysed for the primary outcome	10
	13b	For each group, losses and exclusions after randomisation, together with reasons	11
Recruitment	14a	Dates defining the periods of recruitment and follow-up	11
	14b	Why the trial ended or was stopped	
Baseline data	15	A table showing baseline demographic and clinical characteristics for each group	10
Numbers analysed	16	For each group, number of participants (denominator) included in each analysis and whether the analysis was by original assigned groups	10-11
Outcomes and estimation	17a	For each primary and secondary outcome, results for each group, and the estimated effect size and its precision (such as 95% confidence interval)	9-11
	17b	For binary outcomes, presentation of both absolute and relative effect sizes is recommended	10-11
Ancillary analyses	18	Results of any other analyses performed, including subgroup analyses and adjusted analyses, distinguishing pre-specified from exploratory	
Harms	19	All important harms or unintended effects in each group (for specific guidance see CONSORT for harms)	11
**Discussion**			
Limitations	20	Trial limitations, addressing sources of potential bias, imprecision, and, if relevant, multiplicity of analyses	14
Generalisability	21	Generalisability (external validity, applicability) of the trial findings	12-14
Interpretation	22	Interpretation consistent with results, balancing benefits and harms, and considering other relevant evidence	12-14
**Other information**			
Registration	23	Registration number and name of trial registry	1-2
Protocol	24	Where the full trial protocol can be accessed, if available	7
Funding	25	Sources of funding and other support (such as supply of drugs), role of funders	15

*We strongly recommend reading this statement in conjunction with the CONSORT 2010 Explanation and Elaboration for important clarifications on all the items. If relevant, we also recommend reading CONSORT extensions for cluster randomised trials, non-inferiority and equivalence trials, non-pharmacological treatments, herbal interventions, and pragmatic trials. Additional extensions are forthcoming: for those and for up to date references relevant to this checklist, see www.consort-statement.org.

For peer review only

BMJ Open

A Randomised Controlled Trial to investigate the effectiveness of Local Oestrogen Treatment in Postmenopausal Women Undergoing Pelvic Organ Prolapse Surgery (LOTUS) – a pilot study to assess feasibility of a large multi-centre trial

Journal:	*BMJ Open*
Manuscript ID	bmjopen-2018-025141.R1
Article Type:	Original research
Date Submitted by the Author:	27-Oct-2019
Complete List of Authors:	Verghese, Tina; University of Birmingham, Institute of Metabolism and System Research<br>Middleton, Lee; University of Birmingham, School of Health and Population Sciences<br>Cheed, Versha; University of Birmingham, Birmingham Clinical Trials Unit<br>Leighton, Lisa; University of Birmingham, Birmingham Clinical Trials Unit<br>Daniels, Jane; University of Nottingham, Nottingham Clinical Trials Unit; University of Nottingham Clinical Trials Unit<br>Latthe, Pallavi; Birmingham Women's and Children's NHS Foundation Trust, Obstetrics and Gynaecology
**Primary Subject Heading**:	Obstetrics and gynaecology
Secondary Subject Heading:	Health services research, Patient-centred medicine, Research methods
Keywords:	pilot, feasibility, pelvic organ prolapse, postmenopausal, recurrence, oestrogen

SCHOLARONE™
Manuscripts

BMJ

I, the Submitting Author has the right to grant and does grant on behalf of all authors of the Work (as defined in the below author licence), an exclusive licence and/or a non-exclusive licence for contributions from authors who are: i) UK Crown employees; ii) where BMJ has agreed a CC-BY licence shall apply, and/or iii) in accordance with the terms applicable for US Federal Government officers or employees acting as part of their official duties; on a worldwide, perpetual, irrevocable, royalty-free basis to BMJ Publishing Group Ltd ("BMJ") its licensees and where the relevant Journal is co-owned by BMJ to the co-owners of the Journal, to publish the Work in this journal and any other BMJ products and to exploit all rights, as set out in our licence.

The Submitting Author accepts and understands that any supply made under these terms is made by BMJ to the Submitting Author unless you are acting as an employee on behalf of your employer or a postgraduate student of an affiliated institution which is paying any applicable article publishing charge ("APC") for Open Access articles. Where the Submitting Author wishes to make the Work available on an Open Access basis (and intends to pay the relevant APC), the terms of reuse of such Open Access shall be governed by a Creative Commons licence – details of these licences and which Creative Commons licence will apply to this Work are set out in our licence referred to above.

Other than as permitted in any relevant BMJ Author's Self Archiving Policies, I confirm this Work has not been accepted for publication elsewhere, is not being considered for publication elsewhere and does not duplicate material already published. I confirm all authors consent to publication of this Work and authorise the granting of this licence.

For peer review only

A Randomised Controlled Trial to investigate the effectiveness of Local Oestrogen Treatment in Postmenopausal Women Undergoing Pelvic Organ Prolapse Surgery (LOTUS) – a pilot study to assess feasibility of a large multi-centre trial

For peer review only

Tina Sara Verghese MS MRCOG, Clinical Research Fellow, Institute of Metabolism and System Research College of Medical & Dental Sciences, University of Birmingham, B15 2TT, UK; t.s.verghese@bham.ac.uk

Lee Jonathan Middleton, MSc, Senior Medical Statistician, Birmingham Clinical Trials Unit, University of Birmingham, B15 2TT, UK; l.j.middleton@bham.ac.uk

Versha Cheed, MSc, Medical Statistician, Birmingham Clinical Trials Unit, University of Birmingham, B15 2TT, UK; solankiv@adf.bham.ac.uk

Lisa Leighton LOTUS Trial Co-ordinator, Birmingham Clinical Trials Unit, University of Birmingham, B15 2TT, UK; leightlj@adf.bham.ac.uk

Jane P Daniels PhD MSc MMedSci BSc, Professor of Clinical Trials, Faculty of Medicine & Health Sciences, University of Nottingham, NG7 2UH,UK; jane.daniels@nottingham.ac.uk

Pallavi Manish Latthe MD FRCOG DFFP, Consultant Urogynaecologist, Birmingham Women's and Children's NHS Foundation Trust, B15 2TG, UK; pallavi.latthe@bwnft.nhs.uk

on behalf of the **LOTUS trial collaborative group**

Corresponding author:

For peer review only - http://bmjopen.bmj.com/site/about/guidelines.xhtml

E-mail: t.s.verghese@bham.ac.uk

Formatted for BMJ open

Abstract: 286

Main body: 3733

Abstract

Objective: To evaluate the feasibility of a multicentre randomised controlled trial (RCT) comparing oestrogen treatment with no oestrogen supplementation in women undergoing pelvic organ prolapse (POP) surgery.

Design and setting: A randomised, parallel, open, external pilot trial involving six UK urogynaecology centres (July 2015- August 2016).

Participants: Post-menopausal women with POP opting for surgery, unless involving mesh or for recurrent POP in same compartment.

Intervention: Women were randomised (1:1) to pre and post-operative oestrogen or no treatment. Oestrogen treatment (oestradiol hemihydrate 10mcg vaginal pessaries) commenced 6 weeks prior to surgery (once daily for two weeks, twice weekly for four weeks) and twice weekly for 26 weeks from 6 weeks post-surgery.

Outcome measures: The main outcomes were assessment of eligibility and recruitment rates along with compliance and data completion. Prolapse specific quality of life measures included the Pelvic Floor Distress Inventory Short-Form 20 and the Patient Global Impression of Improvement, completed at 6 and 12 months. Clinical outcomes measured by POP Quantification system, at 6 months post-surgery and post-operative complications.

For peer review only - http://bmjopen.bmj.com/site/about/guidelines.xhtml

Results: 325 women seeking POP surgery were screened over 13 months and 157 (48%) were eligible. Of these, 100 (64%) were randomised, 50 to oestrogen and 50 to no oestrogen treatment, with 89 (44/45 respectively) ultimately having surgery. Of these, 89% (79/89) returned complete questionnaires at six months and 78% (32/41) reported good compliance with oestrogen. No serious adverse events were attributable to oestrogen use.

Conclusions: A large multicentre RCT of oestrogen versus no treatment is feasible as it is possible to randomise and follow up participants with high fidelity. Four predefined feasibility criteria were met. Compliance with treatment regimens is not a barrier. A larger trial is required to definitively address the role of peri-operative oestrogen supplementation.

Trial registration number: ISRCTN46661996.

Keywords

Pilot, feasibility, pelvic organ prolapse, postmenopausal, recurrence, oestrogen.

For peer review only

Article Summary

Strengths and limitations of this study

- This randomised, external, pilot trial had pre-planned feasibility thresholds to judge to assess whether a large trial is feasible.
- The data on number of women at each stage of the trial (screening, randomisation, using the intervention and completing outcome assessments at two follow-up time-points) were collected to estimate recruitment, retention and compliance rates
- There was the potential for post-randomisation exclusions of participants who were ultimately considered unfit for surgery
- Blinding of the oestrogen pessary was not possible, so reported outcomes could be subject to responder bias
- The trial was not designed to be large or long enough to provide convincing evidence for use of pre and post-operative oestrogen treatment

Trial funding

This work was funded by UK National Institute for Health Research, Research for Patient Benefit programme (grant reference PB-PG-0213-30126)

Declaration of interests

PML received sponsorship from Astellas, Contura and Pfizer to attend meetings.

All the other authors declare that they have no competing interests.

For peer review only

Introduction

Pelvic organ prolapse (POP) reportedly occurs in 40-60% of parous women(1). However the true incidence may be underreported, as many women feel embarrassed to seek medical advice. Women with symptomatic POP present with a sense of vaginal bulge, in association with a variety of urinary, bowel and sexual symptoms. In postmenopausal women lack of oestrogen is hypothesised to weaken the pelvic floor muscles(2). Whilst POP surgery is effective, with symptom improvement in 70-88% of women, the risk of recurrent prolapse is high, estimated at 10-30%(1). Efforts to reduce perioperative complications of POP surgery and enhancement of long-term cure are key priorities for the future management of a rapidly ageing population. In postmenopausal women with vaginal atrophy, surgical dissection may be difficult due to thinning of the vaginal walls. In addition, they may be at a higher risk of surgical wound infection, secondary to changes in the vaginal flora. Oestrogen treatment is often used, on its own or in conjunction with various treatment modalities, for vaginal atrophy(3) . However, despite frequent use, there is no robust data on the benefits of pre and postoperative oestrogen treatment in postmenopausal women undergoing POP surgery.

A Cochrane review by Ismail et al, published in 2010 did not find any clear evidence to suggest whether oestrogens for the prevention and treatment of POP and concluded an adequately powered RCT with long term follow up is needed to identify the benefits or risks associated with oestrogen supplementation, including before and after POP surgery(3).

There is a plausible argument for using low-dose oestrogen to improve the vaginal environment and reduce infections at the time of prolapse surgery, but there is little evidence to support its effect on the quality of surgical repair, prolapse cure rates or recurrence. Furthermore, there is no information about any effect in terms of prolapse related symptoms,

For peer review only - http://bmjopen.bmj.com/site/about/guidelines.xhtml

overall quality of life (QoL) and outcomes important to the women. Finally, the duration of oestrogen treatment, and cost-effectiveness compared with current practice are unknown.

There were over 21,000 pelvic floor repair surgeries performed in England in 2014 on women with an average age of 61 years and incurring a hospital stay of 2 days (HES data, Q08.9, P23.1). These data mask the impact of surgical complications on resource use and quality of life, which could be mitigated if perioperative oestrogen was found to be clinically and cost-effective. Moreover, if rates of recurrence rates of prolapse are also reduced as a result of a quality surgical repair, this may lead to substantial cost savings for health systems.

Feasibility studies serve to address the question "can this study be done", by assessing uncertainty in key trial parameters, whilst pilot studies are a small scale version of the definitive study design to test whether all components can work together. There is often overlap between the objectives of any preparatory studies, as the ultimate aim is to maximise the success of a definitive study by identifying problems with recruitment and data collection processes.

The overall aim of this research is to determine whether pre and postoperative local oestrogen treatment is more effective in improving prolapse related patient reported outcomes and reducing recurrence of prolapse symptoms when compared to no treatment. To answer this question a large, multi-centre RCT is required. In preparation, we conducted an external pilot study called Local Oestrogen Treatment in Postmenopausal Women Undergoing Pelvic Organ Prolapse Surgery (LOTUS). We aimed to assess the effectiveness of the patient identification and screening process, including reasons for failure to randomise eligible patients; evaluated the compliance with the treatment schedule; considered robustness and usability of data collection processes during and after the hospital episode and collected limited data on the proposed primary outcome measure and serious adverse events. We report this study as per the CONSORT checklist for reporting a pilot trial.

Methods

For peer review only - http://bmjopen.bmj.com/site/about/guidelines.xhtml

We conducted a randomised parallel group open external pilot trial of pre and post-operative oestrogen treatment versus no treatment in women undergoing POP surgery. A parallel qualitative evaluation of the barriers and facilitators to participation from a women's perspective was presented at International Continence Society Annual Meeting, Florence 2017. The study received a favourable ethical opinion from NHS Research Ethics Committee (NRES Committee West Midlands, REC number 15/WM/0092) and was registered with a primary clinical trials registry (ISRCTN 46661996).

Population

Eligible participants were postmenopausal women who were having POP surgery without use of vaginal mesh. Participants were excluded if they had any of the following: hormone replacement therapy in the last 12 months; previous breast/uterine malignancy or other hormone-dependant neoplasms; genital bleeding of unknown origin; previous thrombo-embolic episodes in relation to oestrogen; known allergy to oestrogens; two or more episodes of culture positive urinary tract infection (UTI) in the last 6 months; voiding dysfunction; previous POP surgery involving mesh or previous POP surgery in the same compartment.

Study conduct and randomisation

All patients thought to fulfil the eligibility criteria were approached with information regarding the trial. Participants were identified in urogynaecology clinics, provided with a patient information sheet and given the opportunity to consider participation.

Once eligibility was confirmed, the trial team, which comprised of urogynaecologists and research nurses from district general hospital as well as university hospital across United Kingdom. They conducted face to face sessions with the participants in order to obtain written informed consent and baseline data. Women who declined trial participation remained eligible to participate in the qualitative study. Randomisation was performed using a web-based central randomisation system (via Birmingham Clinical Trials Unit) to allocate patients to either oestrogen or no treatment in a 1:1 ratio. Minimisation was used to achieve balance between age (<65 years or ≥65 years), parity (<=2 or >2 vaginal births), maximum stage of

For peer review only - http://bmjopen.bmj.com/site/about/guidelines.xhtml

prolapse (I, II or III/IV) and whether a concomitant continence surgery was planned. An oversight committee was formed to provide independent guidance to the Trial Management Committee and to review accruing safety information during the period of recruitment.

Interventions

Those allocated oestrogen (oestradiol hemihydrate 10mcg vaginal pessaries; Vagifem™, Novo Nordisk) were instructed to use the oestrogen pessaries six weeks prior to surgery (once daily for two weeks and twice weekly for four weeks) up to the night before surgery. The treatment was restarted 6 weeks post-operatively, administering twice weekly for 20 weeks (4). Women were encouraged to insert the pessaries into the vagina at the same time of day. However, if a dose was missed patients were advised it should be administered as soon as possible thereafter, provided the next dose was not due. Participants allocated no treatment received the usual care of the randomising centre. The surgical approach to POP repair was at the discretion of the urogynaecological surgeon. It was not possible to blind clinicians or participants due to the nature of the interventions.

Clinical and patient-completed outcomes

Baseline data including clinical history and symptom-related questionnaires were recorded at the pre-surgery clinic appointment. Intraoperative, post-operative (six weeks) and six months post-surgery data including objective assessment of prolapse (POP quantification system [POP-Q]), complications (including urinary tract and vaginal infections) and repeat surgery information were collected by the randomising and/or operating clinician at scheduled appointments. The patient-reported questionnaires were: the Pelvic Floor Distress Inventory Short Form 20 (PFDI-SF20)(5), which comprises of three sub-domains (Pelvic Organ Prolapse Distress Inventory [POPDI-6], Colorectal-Anal Distress Inventory [CRADI-8], Urinary Distress Inventory [UDI-6]); the Pelvic Floor Impact Questionnaire (PFIQ-7), which also has three sub-domains (Pelvic Organ Prolapse Impact [POPIQ-7], Colorectal-Anal Impact [CRAIQ-7], Urinary Impact [UIQ-7]); the Pelvic Organ Prolapse/Urinary Incontinence Sexual Questionnaire (PISQ-12) (6) and Patient Global Impression of

For peer review only - http://bmjopen.bmj.com/site/about/guidelines.xhtml

Improvement (PGI-I) (7). Questionnaires were collected at baseline then at six (at the scheduled appointment) and twelve months, by postal questionnaire. Serious adverse events, reported by clinical staff, were collected throughout the duration of the study.

Sample size

We planned to randomise 100 women; this number would allow us to measure recruitment and compliance rates with 95% confidence interval (CI) width between 10 and 20%. It would also be enough women to estimate the standard deviation (SD) of POPDI-6 with reasonable confidence for future planning of a larger trial (95% CI for SD would be 7 points, assuming the SD is around 20).

Statistical analysis

Feasibility outcomes were considered with simple summary statistics, with uncertainty estimates provided by 95% confidence intervals. Clinical and patient reported outcome measures (PROMs) were analysed with point estimates (RR or mean difference) and 95% confidence intervals, adjusting for the minimisation variables. This was performed using SAS software, version 9.4 (SAS Institute).

They were not subject to hypothesis testing as the size of the sample would not allow reliable assessment of the effect of the interventions. Participants were considered in the groups they were randomised to regardless of compliance in these summaries (intention-to-treat). The dataset used in this manuscript is available on request from Birmingham Clinical Trials Unit.

Pilot outcomes

The following outcomes and targets were set a-priori as being indicative that a larger trial would be feasible to conduct. These were: i) patient eligibility rate (the proportion of screened patients eligible) at least 25%; ii) patient recruitment rate (the proportion of eligible patients randomised) at least 25%; iii) compliance rate (the proportion of patients with good

For peer review only - http://bmjopen.bmj.com/site/about/guidelines.xhtml

compliance to treatment, i.e. at least 75% allocated pessaries used): at least 50%; iv) data completion rate (number of follow-up questionnaires completed at six months): at least 75%.

Patient and Public involvement

Throughout the process of the development and the running of the feasibility study, the study team have actively involved participation from patients and public in order to refine the trial and make the study about postmenopausal women suffering from symptoms of pelvic organ prolapse (POP). The research team conducted a qualitative study to identify the motivations for, and barriers to recruitment and participation in LOTUS. Twenty women were interviewed which provided key insight into trial recruitment barriers. This application benefited from the qualitative study in several ways (e.g. approaching patients and the acceptability of the investigational medicinal product).

Focus groups interview were held through which the research team gained insight from women who were given the opportunity to express their views and felt that further research in this area is much needed

Results

Patients and follow-up

Recruitment took place over a 13-month period between July 2015 and August 2016 in six urogynaecology centres in the UK (Birmingham Women's Hospital, Croydon University Hospital, Basingstoke and North Hampshire Hospital, Walsall Manor Hospital, Royal Stoke University Hospital, James Cook University Hospital, Middlesbrough). Once the target sample size was reached, the study stopped randomising women.

For peer review only - http://bmjopen.bmj.com/site/about/guidelines.xhtml

Of the women who presented with prolapse and wanted surgery, 325 consecutive women were screened for eligibility, 157 were found to be eligible (48%, 95%CI: 40%-56%) and 100 (64%, 95% CI 56-72%) of those eligible were randomised (Figure 1). The minimisation algorithm provide appropriate balance for the minimisation factors; the average age of participants was 66 years with 47% having parity greater than two with an average BMI of 28.2. The maximum stage of prolapse was III or IV (increased severity) for 40% of participants (Table 1).

Of the 100 randomised women, 89 ultimately had surgery (details given in Table 2). Of the other 11, 5 could not have surgery due to health issues, 2 changed their mind about surgery, 2 did not attend their surgical appointment and 2 were diagnosed with cancer. Six month follow-up questionnaires were completed by 79/100 (79%) of participants. Of those that had surgery this equated to 79/89 (89%). Of those who had surgery, 100% had completed intraoperative data and 84/89 (94%) had post-operative data recorded at six weeks.

Compliance

Good compliance with oestrogen treatment (denoted as pessaries used at least >=75% of the expected time) was observed in 79% of participants pre-operatively (34/43), 83% at six weeks (35/42) and 71% at six months (20/28).

Clinical and patient-completed outcomes

Overall, scores from the PFDI-SF20 (POPDI-6 domain) were low at both 6 and 12 months, averaging 14.3 (SD: 16.4) and 15.6 (18.1) out of a maximum of 100, indicating a low level of prolapse-related symptoms at these times. Responses from the PFIQ-7 (POP-IQ-7 domain) were similarly low (Table 3). Scores appeared similar in both groups, but with expected high levels of uncertainty given the limited size of sample. The number of participants reporting being improved (very much better or better) on the PGI-I was 92% (73/79) at 6 months and 89% (70/79) at 12 months (Table 4). The number of participants with an objective rating of

For peer review only - http://bmjopen.bmj.com/site/about/guidelines.xhtml

prolapse failure (from POP-Q) at 6 months was 22/55 (40%) (Table 5). Two repeat incontinence surgeries were recorded in the no treatment group over six months, with none in the oestrogen group.

Safety

More UTIs (8/42; 19% versus 4/42; 10%) - resulting in more antibiotics prescriptions (9/42; 21% versus 5/42; 12%) were reported in the 'no treatment' group than in the oestrogen group. Two serious adverse events were recorded in the oestrogen group - one woman with high temperature was admitted for intravenous antibiotics but culture of vaginal and urine samples were negative, and another woman was diagnosed with leukaemia. Neither were thought to be related to treatment. Two incidental hospitalisations were also recorded in the 'no treatment' group (one case of heavy bleeding thought to be unrelated to surgery and another with pancreatic cancer). No concerns were expressed by the independent oversight committee who met half way through the recruitment period to review the safety data.

Discussion

In this open label randomised study we sought to examine the feasibility of randomising post-menopausal women undergoing POP surgery to receive vaginal oestrogen. Our four key feasibility indicators were met and we have shown that a large multicentre randomised controlled trial (RCT) is feasible. It is possible to randomise and follow up patients with high fidelity over at least 12 months. Oestrogen treatment in the form of vaginal pessaries was well tolerated and consistently applied by most women. As this was a feasibility study with an open-label design, no inferences can be made about the treatment's therapeutic efficacy. However, the data does suggest that further research is warranted. There was evidence that the majority of women in both trial groups did report improvement in their prolapse symptoms, quality of life (QOL) and other aspects of urinary and sexual function. The effect was sustained for at least the first six months after surgery irrespective of oestrogen treatment.

For peer review only

For peer review only - http://bmjopen.bmj.com/site/about/guidelines.xhtml

Among women planning to undergo surgical repair for prolapse, three studies have reported on preoperative vaginal oestrogen by various methods, compared with placebo or no treatment, with a total of 111 participants (8-10). Follow up varied from 12 weeks to three years of postoperative surveillance. No vaginal oestrogen was given following surgery. The overall quality of evidence was poor. Use of vaginal oestrogen improved the vaginal maturation index, a histological measure of the status of the vaginal epithelium, at the time of surgery(11) and increased vaginal epithelial thickness(9) . Vaginal oestrogen application for six weeks preoperatively increased synthesis of mature collagen and increased thickness of the vaginal wall, suggesting this intervention improves both the substrate for suture placement at the time of repair and maintenance of tissue integrity of the pelvic floor(10) . In our study, we did not monitor the vaginal maturation index but we did note that there were fewer UTIs observed in the group that received vaginal oestrogen pre-operatively although the study was not powered for this outcome. The clinicians reported easier planes of dissection in women in the intervention group.

Mikkelsen et al described the three-year postoperative outcomes after preoperative treatment with vaginal oestradiol or placebo tablets before POP repair(9). They included a questionnaire of patient satisfaction, but no standardised or validated metric of prolapse symptoms, discontinuation rates and reasons were reported (8). They suggested that if vaginal oestrogen does improve tissue integrity then therapy may need to be continued postoperatively until the time of complete scar maturation (10). There is no clear evidence to state how long when a women would have adequate scar maturation after a prolapse surgery as this could be dependent on various factors of wound healing. In the LOTUS study women in the intervention group continued oestrogen up to 20 weeks post- surgery. The overall improvement in symptoms was comparable at 6 and 12 months post surgery in the groups. The majority of participants undergoing prolapse surgery in both groups demonstrated improvements in prolapse symptoms (90%) at 12 months, indicating that the benefit of vaginal oestrogen may be marginal, with the majority of improvement in patient symptoms

For peer review only - http://bmjopen.bmj.com/site/about/guidelines.xhtml

pertaining to surgery. This is hypothetical as there has been no RCT with long term follow up on these patients randomised to postoperative local oestrogen vs placebo or no treatment.

Surgical failures requiring repeat POP repairs on an average occur within 2 years after primary surgery. The PROSPECT study reported that 2% (6/395 standard repair) repeat surgeries were required as early as 1 year after primary surgery and 5% (16/348 standard repair) repeat surgery within 2 years(12). The authors concluded that follow up of patients for a minimum of 5 years would be required to assess recurrence of symptoms following POP surgery(12). We need to consider the pros and cons of long term maintenance treatment with vaginal oestrogen in the intervention group in a definitive study including costs, compliance, plateauing of benefits and regression of changes on stopping the treatment. The potential outcomes measured in long-term studies may include recurrence rates, interval of recurrence of prolapse symptoms from index surgery, reduction in urinary tract symptoms and sustained quality of life improvements and cost effectiveness analysis.

Strengths

This feasibility study was conducted over a period of 13 months during which the trial achieved its target recruitment of 100 women from 6 centres in the UK. The study efficiently met all their target criteria within the given time frame and justified that a substantial trial will be achievable.

We were able to capture patient responses by using validated PROMs such as the PFDI - SF20, PFIQ7 and PSIQ 12. The feasibility study has been reported in line with the CONSORT reporting guideline for feasibility/ pilot studies. The feasibility study has allowed the trial team to identify areas in the study process that require fine-tuning ahead of a larger trial. We are considering long term follow up of participants for a minimum of 5 years in order to capture the recurrence rates and relapse of POP symptoms after index surgery. In

For peer review only

addition, we are considering offering participants in the intervention group to continue with long-term maintenance therapy with vaginal oestrogen.

Limitations

Ideally we would have liked to conduct a RCT comparing vaginal oestrogen with a placebo pessary. All of the symptom related outcome measures are patient-completed and potentially prone to reporting bias if the participant is aware of the treatment allocation. However, despite extensive discussions with clinical trial suppliers, we were unable to procure a placebo without it being prohibitively expensive. Oestrogen pessaries are supplied in individually packaged, single-use plastic applicators, which cannot be replicated with a placebo due to the trademark on the packaging. Disassembling the pessary from the applicator to repackage in an unbranded applicator and container would require additional stability testing to confirm the bioavailability of the oestradiol hemihydrate. The manufacturer of Vagifem, Novo Nordisk, declined to provide assistance. Oestradiol is also available as a cream, and a placebo cream could be procured, but the acceptability and adherence to a cream as the route of treatment was not evaluated in this pilot and we should not extrapolate adherence based on pessaries.

A few patients post randomisation could not undergo surgery as they were deemed to be unfit. To avoid this from happening, the patients could have undergone pre-operative assessments to determine surgical and anaesthetic risk and randomised once they were scheduled for surgery. We could potentially have sent telephone, email or text reminders to patient to improve compliance with the post operative pessary use but it would not only be expensive but also not reflect a real life situation.

Conclusion

At this stage, we cannot expect that the outcomes of this pilot study to be directly translated into clinical care as the study was not large enough to be able to detect small to moderate

For peer review only - http://bmjopen.bmj.com/site/about/guidelines.xhtml

realistic-sized difference in rates of prolapse related QoL, nor was the scope wide enough in terms of the number of centres involved to return a generalisable result. However, this study is an important precursor to a larger, substantive trial and provides invaluable information that will help to ensure its success, which ultimately is needed to provide a definitive answer this important question.

Funding

The study was funded by a grant from the National Institute for Health Research for Patient Benefit (grant number: PB-PG-0213-30126). The funder had no role in study design, data collection, interpretation or analysis, or in writing the report for publication.

Acknowledgements

We thank the collaborators, women who recruited into this study, the healthcare professionals at the recruitment sites and the funders.

Miss Ranee Thakar, Croydon

Mrs. Preeti Jain, Walsall

Mr. Christian Phillips, Basingstoke

Mr. Jason Cooper, North Staffordshire

Mr. Athele Khunda, South Tees

Prof Jane Daniels and Lisa Leighton

Patient and Public involvement:

Rev. Ann Simons and Dr. Mary Evans

Trial Oversight committee:

Prof Shakila Thangaratinam, Valerie Morton, Tom Maishman and Deepali Sinha.

Declaration of interests

PML received sponsorship from Astellas, Contura and Pfizer to attend meetings.

For peer review only - http://bmjopen.bmj.com/site/about/guidelines.xhtml

For peer review only

All the other authors declare that they have no competing interests.

Sponsorship

The study sponsor was the Birmingham Women's and Children's NHS Foundation Trust. The sponsor had no role in study design, data collection, interpretation or analysis, or in writing the report for publication.

Authors' contributions

LJM, JPD and PML conceptualised the study as co- applicants. LJM, JPD and PML contributed to the intervention development and design PML oversaw the running of the trial and all the authors contributed to the on-going management of the trial. The LOTUS trial collaborative group collected data for the trial. LJM and VC developed the data analysis plan and summarised the results. TSV and LL evaluated the qualitative data. The manuscript was drafted by TSV with contributions from PML, LJM, JPD, VC and LL. All the authors contributed to the interpretation of the output and revised and reviewed the paper; they are the guarantors. All authors contributed to the writing of the final manuscript and provided critical comments during revisions. All authors approved the final version prior to submission. The Birmingham Clinical Trials Unit undertook the randomisation and data management and monitoring. The authors had full access to all the data from the study. The authors vouch for the accuracy and completeness of the data and analyses. All authors read and approved the final manuscript.

Data sharing statement

The dataset used in this manuscript is available on request from Birmingham Clinical Trials Unit.

For peer review only

Table 1: Baseline Characteristics

Characteristic		Oestrogen (n=50)	No Treatment (n=50)	Overall (n=100)
Age, years[1]	<65	21 (42%)	21 (42%)	42 (42%)
	>=65	29 (58%)	29 (58%)	58 (58%)
	Mean (SD)	65.7 (8.2)	65.9 (8.4)	65.8 (8.3)
Ethnic Group	White	45 (90%)	43 (86%)	88 (88%)
	Asian	2 (4%)	3 (6%)	5 (5%)
	Black	3 (6%)	1 (2%)	4 (4%)
	Mixed	0 (-)	2 (4%)	2 (5%)
	Other ethnic group	0 (-)	1 (2%)	1 (1%)
BMI (mg/k^2)	Mean (SD)	28.1 (5.1)	28.2 (5.9)	28.2 (5.5)
	Missing (N)	2	7	9
Parity[1]	<=2	27 (54%)	26 (52%)	53 (53%)
	>2	23 (46%)	24 (48%)	47 (47%)
Maximum Stage of Prolapse[1]	I	7 (14%)	7 (14%)	14 (14%)
	II	23 (46%)	23 (46%)	46 (46%)
	III/IV	20 (40%)	20 (40%)	40 (40%)
Concomitant continence surgery performed[1]	N (%)	2 (4%)	3 (6%)	5 (5%)
Previous operation for prolapse	N (%)	4 (8%)	4 (8%)	8 (8%)
Anterior	No. of repairs Median (IQR)	1 (1 - 1)	1 (1 - 1)	1 (1 - 1)
Posterior	No. of repairs Median (IQR)	1 (1 - 1)	1 (1 - 1)	1 (1 - 1)
Hysterectomy	N (%)	12 (24%)	8 (16%)	20 (20%)
Vaginal pessary/ring currently in place	N (%)	5 (10%)	8 (16%)	13 (13%)
Physiotherapy treatment for prolapse/urinary incontinence in last 12 months	N (%)	8 (16%)	10 (20%)	18 (18%)
Drug treatment for urinary incontinence	N (%)	0 (-)	4 (8%)	4 (4%)
Treatment for overactive bladder	N (%)	0 (-)	0 (-)	0 (-)

[1] minimisation variable

For peer review only

Table 2: Operative details

**Aspect of surgery**		**Oestrogen (n=44)**	**No Treatment (n=45)**
POP surgery performed	Anterior repair	35 (80%)	34 (76%)
	Posterior repair	16 (36%)	19 (42%)
	Vaginal hysterectomy +/- bilateral salping-oopherectomy	26 (59%)	25 (56%)
	Sacrospinous Fixation	6 (14%)	6 (13%)
Concomitant surgery	Sling/Tension-free vaginal tape	1 (2%)	1 (2%)
	Botulinum toxin A	1 (2%)	1 (2%)
	Suprapublic catheter	1 (2%)	1 (2%)
Ease of dissection	Very easy	6 (14%)	5 (11%)
	Easy	22 (50%)	13 (29%)
	Normal	14 (%)	21 (47%)
	Difficult	2 (5%)	5 (11%)
	Very difficult	0 (-)	1 (2%)
Blood loss (g)	N; Median (IQR)	N=44<br>63 (50 - 125)	N=44<br>54 (50 - 100)
	Missing	0 (-)	1 (2%)
Visceral injury	Bladder injury	0 (-)	0 (-)
	Urethral injury	0 (-)	0 (-)
	Ureteric injury	1 (2%)	0 (-)
	Bowel injury	0 (-)	0 (-)
	Button holing of vagina	3 (7%)	1 (2%)
Suture used – Fascial plication[1]	Polyglactin (Vicryl®, Ethicon/Polysorb®, Medtronic)	10 (23%)	10 (22%)
	Polydioxanone (PDS®-II, Ethicon)	35 (80%)	34 (76%)
	Poligelcarprine (Monocryl®, Ethicon)	0 (-)	0 (-)
	Missing	0	1
Suture used – Vagina[1]	Polyglactin (Vicryl®, Ethicon/Polysorb®, Medtronic)	41 (93%)	42 (93%)
	Polydioxanone (PDS®-II, Ethicon)	5 (11%)	6 (13%)
	Poligelcarprine (Monocryl®, Ethicon)	3 (7%)	1 (2%)
	Vaginal packing used	23 (52%)	28 (62%)
Cather inserted in theatre	Yes	43 (98%)	42 (93%)
Post-operative complications noted before discharge	Infection	1 (2%)	3 (7%)
	Ureteric injury	0 (-)	0 (-)
	Bladder injury	0 (-)	0 (-)
	Bowel injury	0 (-)	0 (-)
	Vascular injury	0 (-)	0 (-)
	Neurological injury	0 (-)	0 (-)
	Peri or post-operative blood transfusion	0 (-)	0 (-)
	Peri or post-operative blood thromboembolism	0 (-)	0 (-)
	Death	0 (-)	0 (-)

For peer review only

For peer review only - http://bmjopen.bmj.com/site/about/guidelines.xhtml

[1] More than one can be selected, hence why numbers do not add up to total.

For peer review only

For peer review only - http://bmjopen.bmj.com/site/about/guidelines.xhtml

Table 3: Results of patient reported outcomes – PFDI20, PFIQ-7 and PISQ-12

Outcome measure Timepoint	Oestrogen N; Mean (SD)	No Treatment N; Mean (SD)	Difference between groups (95% CI)[3]
PFDI-SF20; POPDI-6 Domain (0-100, higher=worse pain)[1]			
Baseline	N=48; 43.4 (24.8)	N=49; 46.0 (24.4)	
6 Month	N=38; 16.3 (20.3)	N=41; 12.5 (11.6)	4.1 (-3.9, 12.1)
12 Month	N=37 16.7 (20.6)	N=41 14.6 (15.6)	4.4 (-4.7, 13.5)
PFDI-SF20; CRAD-8 Domain (0-100, higher=worse pain)[1]			
Baseline	N=48; 26.0 (25.0)	N=48; 24.3 (18.0)	
6 Month	N=38; 12.7 (14.4)	N=41; 15.2 (13.2)	-2.4 (-7.6, 3.4)
12 Month	N=37 12.8 (14.8)	N=42 15.8 (15.8)	-1.7 (-8.0, 4.6)
PFDI-SF20; UDI-6 Domain (0-100, higher=worse pain)[1]			
Baseline	N=48; 34.8 (22.6)	N=49; 37.9 (26.7)	
6 Month	N=38; 16.4 (17.5)	N=41; 17.2 (18.5)	0.5 (-8.9, 7.9)
12 Month	N=36 20.3 (17.5)	N=42 21.5 (25.3)	0.7 (-9.1, 10.4)
PFDI-SF20; Summary Score (0-300, higher=worse pain)[1]			
Baseline	N=48; 104.3 (62.8)	N=48; 107.0 (57.4)	
6 Month	N=38; 45.4 (43.8)	N=41; 45.0 (37.1)	0.6 (-18.4, 17.2)
12 Month	N=36 49.8 (44.1)	N=41 51.1 (47.7)	4.8 (-16.2, 25.8)
PFIQ-7; UIQ-7 Domain (0-100, higher=worse condition)[1]			
Baseline	N=50; 21.0 (25.8)	N=50; 21.5 (24.6)	
6 Month	N=38; 9.6 (21.7)	N=41; 5.9 (11.2)	1.3 (-6.9, 9.5)
12 Month	N=37; 8.9 (16.8)	N=42; 8.0 (13.9)	1.4 (-6.0, 8.8)
PFIQ-7; CRAIQ-7 Domain (0-100, higher=worse condition)[1]			
Baseline	N=50; 12.0 (21.0)	N=50; 8.7 (13.5)	
6 Month	N=38; 4.9 (13.1)	N=41; 2.4 (6.1)	1.6 (-2.5, 5.8)
12 Month	N=36; 4.5 (16.3)	N=42; 2.9 (6.1)	2.2 (-2.8, 7.1)
PFIQ-7; POPIQ-7 Domain (0-100, higher=worse condition)[1]			
Baseline	N=50;	N=50;	

For peer review only - http://bmjopen.bmj.com/site/about/guidelines.xhtml

	17.7 (21.2)	17.1 (22.1)	
6 Month	N=38;<br>5.0 (13.4)	N=41;<br>2.3 (6.1)	2.5 (-2.5, 7.5)
12 Month	N=36;<br>1.9 (6.8)	N=42;<br>1.7 (5.2)	0.2 (-2.7, 3.1)
PFIQ-7; Summary Score (0-300, higher=worse pain)[1]			
Baseline	N=50;<br>50.8 (54.8)	N=50;<br>47.3 (51.9)	
6 Month	N=38;<br>19.5 (39.8)	N=41;<br>10.7 (19.8)	6.2 (-8.7, 21.2)
12 Month	N=36;<br>14.3 (29.1)	N=42;<br>12.7 (18.3)	4.5 (-6.8, 15.8)
PISQ-12 (0-48, higher=better sexual function)[2]			
Baseline	N=18;<br>31.2 (6.5)	N=19;<br>33.1 (7.3)	
6 Month	N=11;<br>33.1 (4.1)	N=14;<br>36.1 (5.7)	-2.4 (-7.0, 2.2)
12 Month	N=11;<br>34.5 (2.7)	N=10;<br>32.8 (5.1)	3.4 (0.6, 6.2)

[1]<0 indicates less pain/better condition failures with oestrogen

[2]>0 indicates better with oestrogen

[3]Differences are adjusted for baseline score and minimisation variables

For peer review only

For peer review only - http://bmjopen.bmj.com/site/about/guidelines.xhtml

Table 4: Results of patient reported outcomes – patient global impression of improvement (PGI-I)

Timepoint PGI-I response		Oestrogen	No Treatment	Relative risk (95%CI)[3]
**6 Week**		N=42	N=42	
1 = Very much better	N (%)	22 (52%)	14 (33%)	
2 = Much better	N (%)	16 (38%)	18 (43%)	
3 = A little better	N (%)	4 (10%)	8 (19%)	
4 = No change	N (%)	0 (-)	2 (5%)	
5 = A little worse	N (%)	0 (-)	0 (-)	
6 = Much Worse	N (%)	0 (-)	0 (-)	
7 = Very Much Worse	N (%)	0 (-)	0 (-)	
Better score[1]	N (%)	42 (100%)	40 (95%)	1.05 (0.98 – 1.12)
Worse score[2]	N (%)	0 (-)	2 (5%)	
**6 Month**		N=38	N=41	
1 = Very much better	N (%)	13 (34%)	18 (44%)	
2 = Much better	N (%)	16 (42%)	17 (41%)	
3 = A little better	N (%)	4 (11%)	5 (12%)	
4 = No change	N (%)	3 (8%)	0 (-)	
5 = A little worse	N (%)	2 (5%)	1 (2%)	
6 = Much Worse	N (%)	0 (-)	0 (-)	
7 = Very Much Worse	N (%)	0 (-)	0 (-)	
Better score[1]	N (%)	33 (87%)	40 (98%)	0.89 (0.78 – 1.02)
Worse score[2]	N (%)	5 (13%)	1 (2%)	
**12 Month**		N=37	N=42	
1 = Very much better	N (%)	14 (38%)	17 (40%)	
2 = Much better	N (%)	15 (41%)	17 (40%)	
3 = A little better	N (%)	3 (8%)	4 (10%)	
4 = No change	N (%)	4 (11%)	2 (5%)	
5 = A little worse	N (%)	1 (3%)	0 (-)	
6 = Much Worse	N (%)	0 (-)	2 (5%)	
7 = Very Much Worse	N (%)	0 (-)	0 (-)	
Better score[1]	N (%)	32 (86%)	38 (90%)	0.96 (0.81 – 1.12)
Worse score[2]	N (%)	5 (14%)	4 (10%)	

[1]Better score is derived from scores 1 (Very much better) to 3 (A little better)

[2]Worse score is derived from scores 4 (No change) to 7 (Very much worse)

[3]>1 indicates better with oestrogen

For peer review only - http://bmjopen.bmj.com/site/about/guidelines.xhtml

Table 5: Results of POP-Q assessment

Anatomical site in vagina Timepoint	**Oestrogen N; Mean (SD)**	**No Treatment N; Mean (SD)**	**Difference between groups (95% CI)**
Aa (anterior wall), cm			
Baseline	N=49; 0.1 (1.9)	N=48; -0.3 (1.7)	
6 Months[1]	N=26; -1.7 (2.0)	N=29; -2.6 (2.0)	0.7 (0.1, 1.3)
Ba (anterior edge), cm			
Baseline	N=49; 0.2 (2.0)	N=48; 0.1 (1.8)	
6 Months[1]	N=25; -2.0 (2.0)	N=29; -2.9 (1.9)	0.6 (0.0, 1.2)
C (cervix/cuff), cm			
Baseline	N=49; -3.7 (2.5)	N=48; -3.4 (3.4)	
6 Months[1]	N=25; -2.9 (2.8)	N=29; -2.2 (3.6)	-0.2 (-1.3, 0.9)
Ap (posterior wall), cm			
Baseline	N=49; -1.2 (1.5)	N=48; -1.0 (1.7)	
6 Months[1]	N=26; -0.8 (1.7)	N=29; -1.0 (1.7)	0.5 (-0.2, 1.1)
Bp (posterior edge), cm			
Baseline	N=49; -1.1 (1.6)	N=48; -1.0 (1.8)	
6 Months[1]	N=26; -0.9 (1.8)	N=29; -1.0 (1.8)	0.4 (-0.3, 1.1)
D (posterior fornix), cm			
Baseline	N=39; -5.3 (1.9)	N=40; -5.1 (2.6)	
6 Months[1]	N=7; -1.3 (1.5)	N=4; -1.8 (1.9)	0.7 (-6.2, 7.5)
Total vaginal length, cm			
Baseline	N=49; 8.5 (1.1)	N=48; 8.0 (1.6)	
6 Months[2]	N=26; -0.2 (1.3)	N=29; -0.5 (1.7)	0.3 (0.4, 0.9)
Objective prolapse (any of Aa, Ba, C, Ap, Bp, or D>0) at six months			
	**N (%)**	**N (%)**	**Relative risk (95%CI)[3]**
Failure	9/26	13/29	0.77 (0.40 – 1.50)

[1]Change from baseline adjusted for baseline; difference <0 indicates better with oestrogen

[2]Adjusted for baseline length; difference<0 indicates better with oestrogen

[3]<1 indicates less prolapse failures with oestrogen

Figure1: Flow of participants through the trial

For peer review only - http://bmjopen.bmj.com/site/about/guidelines.xhtml

References

1. Maher C, Feiner B, Baessler K, Christmann-Schmid C, Haya N, Brown J. Surgery for women with anterior compartment prolapse. The Cochrane database of systematic reviews. 2016;11:CD004014.
2. Nilsson K, Risberg B, Heimer G. The vaginal epithelium in the postmenopause--cytology, histology and pH as methods of assessment. Maturitas. 1995;21(1):51-6.
3. Ismail SI, Bain C, Hagen S. Oestrogens for treatment or prevention of pelvic organ prolapse in postmenopausal women. The Cochrane database of systematic reviews. 2010(9):CD007063.
4. [Available from: https://www.birmingham.ac.uk/Documents/college-mds/trials/bctu/lotus/LOTUS-protocol-V2.0.pdf

.
5. Barber MD WM BR. Short forms of two condition-specific quality-of-life questionnaires for women with pelvic floor disorders (PFDI-20 and PFIQ-7). . Am J Obstet Gynecol

2005.
6. Rogers RG, Coates KW, Kammerer-Doak D, Khalsa S, Qualls C. A short form of the Pelvic Organ Prolapse/Urinary Incontinence Sexual Questionnaire (PISQ-12). Int Urogynecol J Pelvic Floor Dysfunct. 2003;14(3):164-8; discussion 8.
7. Srikrishna S, Robinson D, Cardozo L. Validation of the Patient Global Impression of Improvement (PGI-I) for urogenital prolapse. Int Urogynecol J. 2010;21(5):523-8.
8. Felding C, Mikkelsen AL, Clausen HV, Loft A, Larsen LG. Preoperative treatment with oestradiol in women scheduled for vaginal operation for genital prolapse. A randomised, double-blind trial. Maturitas. 1992;15(3):241-9.
9. Mikkelsen AL, Felding C, Clausen HV. Clinical effects of preoperative oestradiol treatment before vaginal repair operation. A double-blind, randomized trial. Gynecol Obstet Invest. 1995;40(2):125-8.
10. Vaccaro CM, Mutema GK, Fellner AN, Crisp CC, Estanol MV, Kleeman SD, et al. Histologic and cytologic effects of vaginal estrogen in women with pelvic organ prolapse: a randomized controlled trial. Female Pelvic Med Reconstr Surg. 2013;19(1):34-9.
11. Moalli PA, Shand SH, Zyczynski HM, Gordy SC, Meyn LA. Remodeling of vaginal connective tissue in patients with prolapse. Obstet Gynecol. 2005;106(5 Pt 1):953-63.
12. Glazener CM, Breeman S, Elders A, Hemming C, Cooper KG, Freeman RM, et al. Mesh, graft, or standard repair for women having primary transvaginal anterior or posterior compartment prolapse surgery: two parallel-group, multicentre, randomised, controlled trials (PROSPECT). Lancet. 2017;389(10067):381-92.

For peer review only - http://bmjopen.bmj.com/site/about/guidelines.xhtml

Figure 1: Flow of participants through the trial

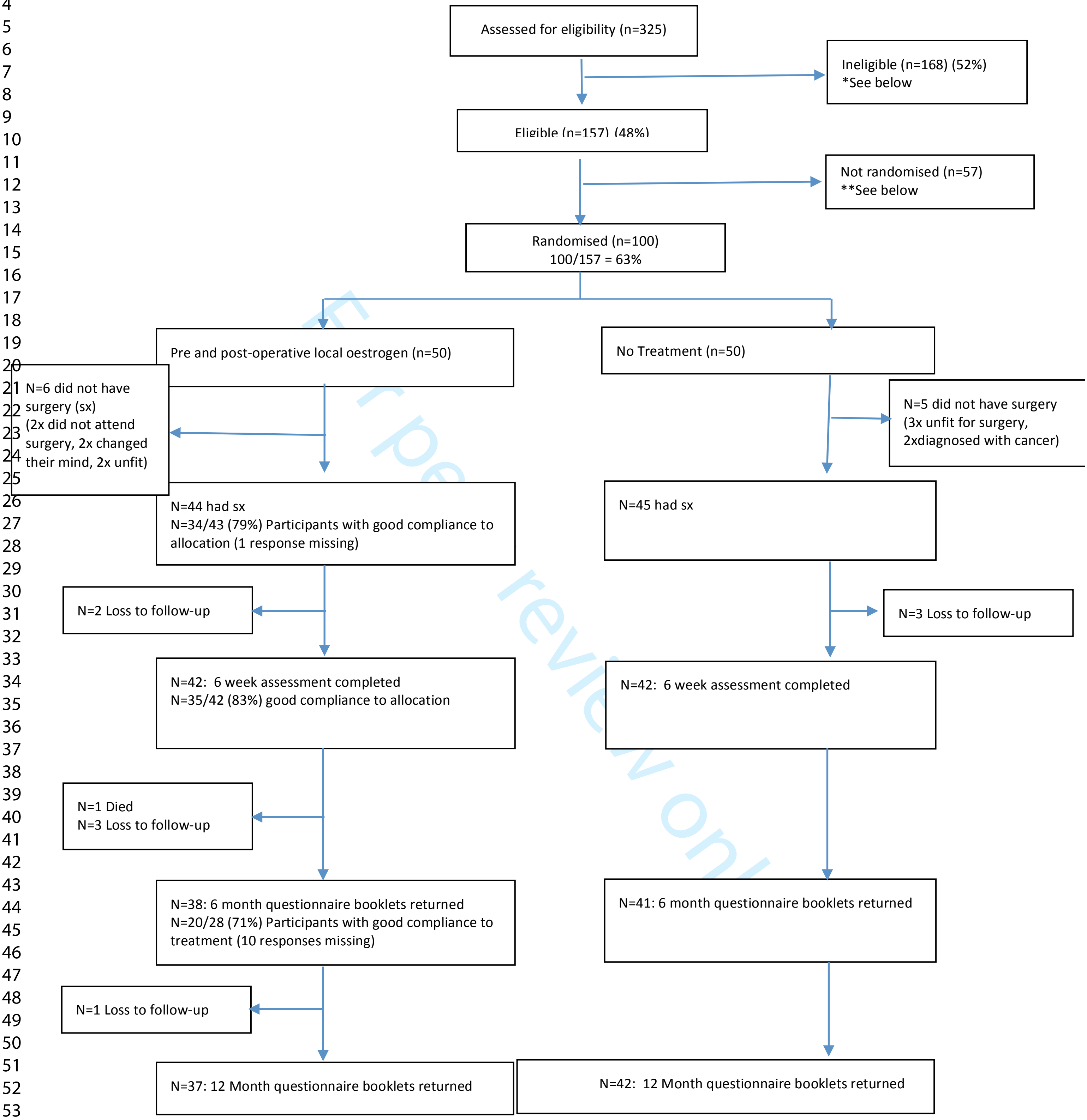

*Reasons for Ineligibility N=168 (%): No prolapse surgery required 38 (22), Recurrence of prolapse in the same compartment 32(19), Has had previous breast or uterine malignancy 20 (12), Undergoing HRT treatment 17 (10), Unable to understand English 16 (9), Genital bleeding of unknown origin 15 (9), Has had previous hormone-dependent neoplasms 13 (8), Previous POP surgery involving mesh 6 (3), Previous thrombo-embolic episode related to oestrogen therapy 4 (2), Two or more culture positive UTI episodes in the last 6 months 1 (<1), Other 6 (3)

**Reasons for Non Randomisation N=57 (%):Patient did not attend clinic 19 (33), Not enough time to discuss trial and gain informed consent 14 (25), Declined Consent/not willing to be randomised 9 (16), Not willing to wait 6 weeks for surgery 5 (9), Unable to gain consent 4 (7), Did not want to complete questionnaires, 1(2)

For peer review only - http://bmjopen.bmj.com/site/about/guidelines.xhtml

For peer review only

CONSORT 2010 checklist of information to include when reporting a randomised trial*

Section/Topic	Item No	Checklist item	Reported on page No
**Title and abstract**			
	1a	Identification as a randomised trial in the title	1
	1b	Structured summary of trial design, methods, results, and conclusions (for specific guidance see CONSORT for abstracts)	2
**Introduction**			
Background and objectives	2a	Scientific background and explanation of rationale	5
	2b	Specific objectives or hypotheses	5
**Methods**			
Trial design	3a	Description of trial design (such as parallel, factorial) including allocation ratio	6
	3b	Important changes to methods after trial commencement (such as eligibility criteria), with reasons	7
Participants	4a	Eligibility criteria for participants	7
	4b	Settings and locations where the data were collected	7
Interventions	5	The interventions for each group with sufficient details to allow replication, including how and when they were actually administered	8
Outcomes	6a	Completely defined pre-specified primary and secondary outcome measures, including how and when they were assessed	8
	6b	Any changes to trial outcomes after the trial commenced, with reasons	8
Sample size	7a	How sample size was determined	9
	7b	When applicable, explanation of any interim analyses and stopping guidelines	9
Randomisation:			
Sequence generation	8a	Method used to generate the random allocation sequence	7
	8b	Type of randomisation; details of any restriction (such as blocking and block size)	7
Allocation concealment mechanism	9	Mechanism used to implement the random allocation sequence (such as sequentially numbered containers), describing any steps taken to conceal the sequence until interventions were assigned	7
Implementation	10	Who generated the random allocation sequence, who enrolled participants, and who assigned participants to interventions	7
Blinding	11a	If done, who was blinded after assignment to interventions (for example, participants, care providers, those	-

For peer review only - http://bmjopen.bmj.com/site/about/guidelines.xhtml

		assessing outcomes) and how	
	11b	If relevant, description of the similarity of interventions	-
Statistical methods	12a	Statistical methods used to compare groups for primary and secondary outcomes	9
	12b	Methods for additional analyses, such as subgroup analyses and adjusted analyses	9
**Results**			
Participant flow (a diagram is strongly recommended)	13a	For each group, the numbers of participants who were randomly assigned, received intended treatment, and were analysed for the primary outcome	10
	13b	For each group, losses and exclusions after randomisation, together with reasons	11
Recruitment	14a	Dates defining the periods of recruitment and follow-up	11
	14b	Why the trial ended or was stopped	
Baseline data	15	A table showing baseline demographic and clinical characteristics for each group	10
Numbers analysed	16	For each group, number of participants (denominator) included in each analysis and whether the analysis was by original assigned groups	10-11
Outcomes and estimation	17a	For each primary and secondary outcome, results for each group, and the estimated effect size and its precision (such as 95% confidence interval)	9-11
	17b	For binary outcomes, presentation of both absolute and relative effect sizes is recommended	10-11
Ancillary analyses	18	Results of any other analyses performed, including subgroup analyses and adjusted analyses, distinguishing pre-specified from exploratory	
Harms	19	All important harms or unintended effects in each group (for specific guidance see CONSORT for harms)	11
**Discussion**			
Limitations	20	Trial limitations, addressing sources of potential bias, imprecision, and, if relevant, multiplicity of analyses	14
Generalisability	21	Generalisability (external validity, applicability) of the trial findings	12-14
Interpretation	22	Interpretation consistent with results, balancing benefits and harms, and considering other relevant evidence	12-14
**Other information**			
Registration	23	Registration number and name of trial registry	1-2
Protocol	24	Where the full trial protocol can be accessed, if available	7
Funding	25	Sources of funding and other support (such as supply of drugs), role of funders	15

*We strongly recommend reading this statement in conjunction with the CONSORT 2010 Explanation and Elaboration for important clarifications on all the items. If relevant, we also recommend reading CONSORT extensions for cluster randomised trials, non-inferiority and equivalence trials, non-pharmacological treatments, herbal interventions, and pragmatic trials. Additional extensions are forthcoming: for those and for up to date references relevant to this checklist, see www.consort-statement.org.

For peer review only

BMJ Open

A Randomised Controlled Trial to investigate the effectiveness of Local Oestrogen Treatment in Postmenopausal Women Undergoing Pelvic Organ Prolapse Surgery (LOTUS) – a pilot study to assess feasibility of a large multi-centre trial

Journal:	*BMJ Open*
Manuscript ID	bmjopen-2018-025141.R2
Article Type:	Original research
Date Submitted by the Author:	07-Feb-2020
Complete List of Authors:	Verghese, Tina; University of Birmingham, Institute of Metabolism and System Research<br>Middleton, Lee; University of Birmingham, School of Health and Population Sciences<br>Cheed, Versha; University of Birmingham, Birmingham Clinical Trials Unit<br>Leighton, Lisa; University of Birmingham, Birmingham Clinical Trials Unit<br>Daniels, Jane; University of Nottingham, Nottingham Clinical Trials Unit; University of Nottingham Clinical Trials Unit<br>Latthe, Pallavi; Birmingham Women's and Children's NHS Foundation Trust, Obstetrics and Gynaecology
**Primary Subject Heading**:	Obstetrics and gynaecology
Secondary Subject Heading:	Health services research, Patient-centred medicine, Research methods
Keywords:	pilot, feasibility, pelvic organ prolapse, postmenopausal, recurrence, oestrogen

SCHOLARONE™
Manuscripts

BMJ

I, the Submitting Author has the right to grant and does grant on behalf of all authors of the Work (as defined in the below author licence), an exclusive licence and/or a non-exclusive licence for contributions from authors who are: i) UK Crown employees; ii) where BMJ has agreed a CC-BY licence shall apply, and/or iii) in accordance with the terms applicable for US Federal Government officers or employees acting as part of their official duties; on a worldwide, perpetual, irrevocable, royalty-free basis to BMJ Publishing Group Ltd ("BMJ") its licensees and where the relevant Journal is co-owned by BMJ to the co-owners of the Journal, to publish the Work in this journal and any other BMJ products and to exploit all rights, as set out in our licence.

The Submitting Author accepts and understands that any supply made under these terms is made by BMJ to the Submitting Author unless you are acting as an employee on behalf of your employer or a postgraduate student of an affiliated institution which is paying any applicable article publishing charge ("APC") for Open Access articles. Where the Submitting Author wishes to make the Work available on an Open Access basis (and intends to pay the relevant APC), the terms of reuse of such Open Access shall be governed by a Creative Commons licence – details of these licences and which Creative Commons licence will apply to this Work are set out in our licence referred to above.

Other than as permitted in any relevant BMJ Author's Self Archiving Policies, I confirm this Work has not been accepted for publication elsewhere, is not being considered for publication elsewhere and does not duplicate material already published. I confirm all authors consent to publication of this Work and authorise the granting of this licence.

For peer review only

A Randomised Controlled Trial to investigate the effectiveness of Local Oestrogen Treatment in Postmenopausal Women Undergoing Pelvic Organ Prolapse Surgery (LOTUS) – a pilot study to assess feasibility of a large multi-centre trial

Tina Sara Verghese MS MRCOG, Clinical Research Fellow, Institute of Metabolism and System Research College of Medical & Dental Sciences, University of Birmingham, B15 2TT, UK; t.s.verghese@bham.ac.uk

Lee Jonathan Middleton, MSc, Senior Medical Statistician, Birmingham Clinical Trials Unit, University of Birmingham, B15 2TT, UK; l.j.middleton@bham.ac.uk

Versha Cheed, MSc, Medical Statistician, Birmingham Clinical Trials Unit, University of Birmingham, B15 2TT, UK; solankiv@adf.bham.ac.uk

Lisa Leighton LOTUS Trial Co-ordinator, Birmingham Clinical Trials Unit, University of Birmingham, B15 2TT, UK; leightlj@adf.bham.ac.uk

Jane P Daniels PhD MSc MMedSci BSc, Professor of Clinical Trials, Faculty of Medicine & Health Sciences, University of Nottingham, NG7 2UH,UK; jane.daniels@nottingham.ac.uk

Pallavi Manish Latthe MD FRCOG DFFP, Consultant Urogynaecologist, Birmingham Women's and Children's NHS Foundation Trust, B15 2TG, UK; pallavi.latthe@bwnft.nhs.uk

on behalf of the **LOTUS trial collaborative group**

Corresponding author:

For peer review only - http://bmjopen.bmj.com/site/about/guidelines.xhtml

E-mail: t.s.verghese@bham.ac.uk

Formatted for BMJ open

Abstract: 275

Main body: 3802

For peer review only

For peer review only - http://bmjopen.bmj.com/site/about/guidelines.xhtml

Abstract

Objective: To evaluate the feasibility of a multicentre randomised controlled trial (RCT) comparing oestrogen treatment with no oestrogen supplementation in women undergoing pelvic organ prolapse (POP) surgery.

Design and setting: A randomised, parallel, open, external pilot trial involving six UK urogynaecology centres (July 2015- August 2016).

Participants: Post-menopausal women with POP opting for surgery, unless involving mesh or for recurrent POP in same compartment.

Intervention: Women were randomised (1:1) to pre and post-operative oestrogen or no treatment. Oestrogen treatment (oestradiol hemihydrate 10mcg vaginal pessaries) commenced 6 weeks prior to surgery (once daily for two weeks, twice weekly for four weeks) and twice weekly for 26 weeks from 6 weeks post-surgery.

Outcome measures: The main outcomes were assessment of eligibility and recruitment rates along with compliance and data completion. To obtain estimates for important aspects of the protocol to allow development of a definitive trial.

Results: 325 women seeking POP surgery were screened over 13 months and 157 (48%) were eligible. Of these, 100 (64%) were randomised, 50 to oestrogen and 50 to no oestrogen treatment, with 89 (44/45 respectively) ultimately having surgery. Of these, 89% (79/89) returned complete questionnaires at six months and 78% (32/41) reported good compliance with oestrogen. No serious adverse events were attributable to oestrogen use.

For peer review only - http://bmjopen.bmj.com/site/about/guidelines.xhtml

Conclusions: A large multicentre RCT of oestrogen versus no treatment is feasible as it is possible to randomise and follow up participants with high fidelity. Four predefined feasibility criteria were met. Compliance with treatment regimens is not a barrier. A larger trial is required to definitively address the role of peri-operative oestrogen supplementation.

Trial registration number: ISRCTN46661996.

Keywords

Pilot, feasibility, pelvic organ prolapse, postmenopausal, recurrence, oestrogen.

For peer review only

Article Summary

Strengths and limitations of this study

- This randomised, external, pilot trial had pre-planned feasibility thresholds to judge to assess whether a large trial is feasible.
- The data on number of women at each stage of the trial (screening, randomisation, using the intervention and completing outcome assessments at two follow-up time-points) were collected to estimate recruitment, retention and compliance rates
- There was the potential for post-randomisation exclusions of participants who were ultimately considered unfit for surgery
- Blinding of the oestrogen pessary was not possible, so reported outcomes could be subject to responder bias
- The trial was not designed to be large or long enough to provide convincing evidence for use of pre and post-operative oestrogen treatment

Trial funding

This work was funded by UK National Institute for Health Research, Research for Patient Benefit programme (grant reference PB-PG-0213-30126)

Declaration of interests

PML received sponsorship from Astellas, Contura and Pfizer to attend meetings.

All the other authors declare that they have no competing interests.

For peer review only

Introduction

Pelvic organ prolapse (POP) is the descent of one or more of the anterior vaginal wall, posterior vaginal wall, the uterus (cervix), or the apex of the vagina (after hysterectomy) (1). This definition is based on anatomical change; however, diagnosis of prolapse should start with symptoms and relate these symptoms to the descent of pelvic organs (1). Women with symptomatic POP present with a sense of vaginal bulge, in association with a variety of urinary, bowel and sexual symptoms. In postmenopausal women lack of oestrogen is hypothesised to weaken the pelvic floor muscles(2). Whilst POP surgery is effective, with symptom improvement in 70-88% of women, the risk of recurrent prolapse is high, estimated at 10-30%(1). Efforts to reduce perioperative complications of POP surgery and enhancement of long-term cure are key priorities for the future management of a rapidly ageing population. In postmenopausal women with vaginal atrophy, surgical dissection may be difficult due to thinning of the vaginal walls. In addition, they may be at a higher risk of surgical wound infection, secondary to changes in the vaginal flora. Oestrogen treatment is often used, on its own or in conjunction with various treatment modalities, for vaginal atrophy(3) . However, despite frequent use, there are no robust data on the benefits of pre and postoperative oestrogen treatment in postmenopausal women undergoing POP surgery.

A Cochrane review by Ismail et al, published in 2010 did not find any clear evidence to suggest whether oestrogens for the prevention and treatment of POP and concluded an adequately powered RCT with long term follow up is needed to identify the benefits or risks associated with oestrogen supplementation, including before and after POP surgery(3).

There is a plausible argument for using low-dose oestrogen to improve the vaginal environment and reduce infections at the time of prolapse surgery, but there is little evidence to support its effect on the quality of surgical repair, prolapse cure rates or recurrence.

For peer review only

For peer review only - http://bmjopen.bmj.com/site/about/guidelines.xhtml

Furthermore, there is no information about any effect in terms of prolapse related symptoms, overall quality of life (QoL) and outcomes important to the women. Finally, the duration of oestrogen treatment, and cost-effectiveness compared with current practice are unknown.

There were over 21,000 pelvic floor repair surgeries performed in England in 2014 on women with an average age of 61 years and incurring a hospital stay of 2 days (HES data, Q08.9, P23.1). These data mask the impact of surgical complications on resource use and quality of life, which could be mitigated if perioperative oestrogen was found to be clinically and cost-effective. Moreover, if this use reduces failure and recurrence rates, this would save cost.

Feasibility studies serve to address the question "can this study be done", by assessing uncertainty in key trial parameters, whilst pilot studies are a small scale version of the definitive study design to test whether all components can work together. There is often overlap between the objectives of any preparatory studies, as the ultimate aim is to maximise the success of a definitive study by identifying problems with recruitment and data collection processes (4).

The aim of the feasibility study was to find out if an appropriately powered randomised controlled trial (RCT) can be realistically undertaken The overall aim of this research is to determine whether pre and postoperative local oestrogen treatment is more effective in improving prolapse related patient reported outcomes and reducing recurrence of prolapse symptoms when compared to no treatment. To answer this question a large, multi-centre RCT is required. We report this study as per the CONSORT checklist for reporting a pilot trial (5).

Methods

We conducted a randomised parallel group open external pilot trial of pre and post-operative oestrogen treatment versus no treatment in women undergoing POP surgery. The study received a favourable ethical opinion from NHS Research Ethics Committee (NRES

For peer review only - http://bmjopen.bmj.com/site/about/guidelines.xhtml

Committee West Midlands, REC number 15/WM/0092) and was registered with a primary clinical trials registry (ISRCTN 46661996).

Population

Eligible participants were postmenopausal women who were having POP surgery without use of vaginal mesh. Participants were excluded if they had any of the following: hormone replacement therapy in the last 12 months; previous breast/uterine malignancy or other hormone-dependant neoplasms; genital bleeding of unknown origin; previous thrombo-embolic episodes in relation to oestrogen; known allergy to oestrogens; two or more episodes of culture positive urinary tract infection (UTI) in the last 6 months; voiding dysfunction; previous POP surgery involving mesh or previous POP surgery in the same compartment.

Study conduct and randomisation

All patients thought to fulfil the eligibility criteria were approached with information regarding the trial. Participants were identified in urogynaecology clinics, provided with a patient information sheet and given the opportunity to consider participation.

Once eligibility was confirmed, the trial team, which comprised of urogynaecologists and research nurses from district general hospitals as well as university hospitals across United Kingdom. They conducted face to face sessions with the participants in order to obtain written informed consent and baseline data. Women who declined trial participation remained eligible to participate in the qualitative study. Randomisation was performed using a web-based central randomisation system (via Birmingham Clinical Trials Unit) to allocate patients to either oestrogen or no treatment in a 1:1 ratio. Minimisation was used to achieve balance between age (<65 years or ≥65 years), parity (<=2 or >2 vaginal births), maximum stage of prolapse (I, II or III/IV) and whether a concomitant continence surgery was planned. An oversight committee was formed to provide independent guidance to the Trial Management Committee and to review accruing safety information during the period of recruitment.

Interventions

For peer review only - http://bmjopen.bmj.com/site/about/guidelines.xhtml

Those allocated oestrogen (oestradiol hemihydrate 10mcg vaginal pessaries; Vagifem™, Novo Nordisk) were instructed to use the oestrogen pessaries six weeks prior to surgery (once daily for two weeks and twice weekly for four weeks) up to the night before surgery. The treatment was restarted 6 weeks post-operatively, administering twice weekly for 20 weeks (6). Women were encouraged to insert the pessaries into the vagina at the same time of day. However, if a dose was missed patients were advised it should be administered as soon as possible thereafter, provided the next dose was not due. Participants allocated no treatment received the usual care of the randomising centre. The surgical approach to POP repair was at the discretion of the urogynaecological surgeon. It was not possible to blind clinicians or participants due to the nature of the interventions.

Clinical and patient-completed outcomes

Primary outcome measure of the feasibility study

To obtain estimates for important aspects of the protocol to allow development of a definitive trial.

Secondary outcome measure of the feasibility study

1. Assessment of effectiveness of patient identification and screening processes

2. Assessment of the effectiveness of the randomisation process of patients

3. Evaluation of robustness of data collection processes

4. The proportion of patients followed up at six months

5. Derivation of the preliminary data from clinical outcome measures (e.g.PFDI-SF20) to inform the sample size calculation for the substantive study

Baseline data including clinical history and symptom-related questionnaires were recorded at the pre-surgery clinic appointment. Intraoperative, post-operative (six weeks) and six months post-surgery data including objective assessment of prolapse (POP quantification system [POP-Q]), complications (including urinary tract and vaginal infections) and repeat surgery information were collected by the randomising and/or operating clinician at scheduled appointments. The patient-reported questionnaires were: the Pelvic Floor Distress Inventory

For peer review only - http://bmjopen.bmj.com/site/about/guidelines.xhtml

Short Form 20 (PFDI-SF20)(7), which comprises of three sub-domains (Pelvic Organ Prolapse Distress Inventory [POPDI-6], Colorectal-Anal Distress Inventory [CRADI-8], Urinary Distress Inventory [UDI-6]); the Pelvic Floor Impact Questionnaire (PFIQ-7), which also has three sub-domains (Pelvic Organ Prolapse Impact [POPIQ-7], Colorectal-Anal Impact [CRAIQ-7], Urinary Impact [UIQ-7]); the Pelvic Organ Prolapse/Urinary Incontinence Sexual Questionnaire (PISQ-12) (8) and Patient Global Impression of Improvement (PGI-I) (9). Questionnaires were collected at baseline then at six (at the scheduled appointment) and twelve months, by postal questionnaire. Serious adverse events, reported by clinical staff, were collected throughout the duration of the study.

Sample size

We planned to randomise 100 women; this number would allow us to measure recruitment and compliance rates with 95% confidence interval (CI) width between 10 and 20%. It would also be enough women to estimate the standard deviation (SD) of POPDI-6 with reasonable confidence for future planning of a larger trial (95% CI for SD would be 7 points, assuming the SD is around 20).

Statistical analysis

Feasibility outcomes were considered with simple summary statistics, with uncertainty estimates provided by 95% confidence intervals. Clinical and patient reported outcome measures (PROMs) were analysed with point estimates (RR or mean difference) and 95% confidence intervals, adjusting for the minimisation variables. This was performed using SAS software, version 9.4 (SAS Institute).

They were not subject to hypothesis testing as the size of the sample would not allow reliable assessment of the effect of the interventions. Participants were considered in the groups they were randomised to regardless of compliance in these summaries (intention-to-treat). The dataset used in this manuscript is available on request from Birmingham Clinical Trials Unit.

For peer review only - http://bmjopen.bmj.com/site/about/guidelines.xhtml

Pilot outcomes

The following outcomes and targets were set a-priori as being indicative that a larger trial would be feasible to conduct. These were: i) patient eligibility rate (the proportion of screened patients eligible) at least 25%; ii) patient recruitment rate (the proportion of eligible patients randomised) at least 25%; iii) compliance rate (the proportion of patients with good compliance to treatment, i.e. at least 75% allocated pessaries used): at least 50%; iv) data completion rate (number of follow-up questionnaires completed at six months): at least 75%.

Patient and Public involvement

Throughout the process of the development and the running of the feasibility study, the study team have actively involved participation from patients and public in order to refine the trial and make the study about postmenopausal women suffering from symptoms of pelvic organ prolapse (POP). The research team conducted a qualitative study to identify the motivations for, and barriers to recruitment and participation in LOTUS. Twenty women were interviewed which provided key insight into trial recruitment barriers. This application benefited from the qualitative study in several ways (e.g. approaching patients and the acceptability of the investigational medicinal product).

Focus group interviews were held through which the research team gained insight from women who were given the opportunity to express their views and felt that further research in this area is much needed

Results

Patients and follow-up

Recruitment took place over a 13-month period between July 2015 and August 2016 in six urogynaecology centres in the UK (Birmingham Women's Hospital, Croydon University Hospital, Basingstoke and North Hampshire Hospital, Walsall Manor Hospital, Royal Stoke University Hospital, James Cook University Hospital, Middlesbrough). Once the target sample size was reached, the study stopped randomising women.

For peer review only - http://bmjopen.bmj.com/site/about/guidelines.xhtml

Of the women who presented with prolapse and wanted surgery, 325 consecutive women were screened for eligibility, 157 were found to be eligible (48%, 95%CI: 40%-56%) and 100 (64%, 95% CI 56-72%) of those eligible were randomised (Figure 1). The minimisation algorithm provide appropriate balance for the minimisation factors; the average age of participants was 66 years with 47% having parity greater than two with an average BMI of 28.2. The maximum stage of prolapse was III or IV (increased severity) for 40% of participants (Table 1).

Of the 100 randomised women, 89 ultimately had surgery (details given in Table 2). Of the other 11, 5 could not have surgery due to health issues, 2 changed their mind about surgery, 2 did not attend their surgical appointment and 2 were diagnosed with cancer. Six month follow-up questionnaires were completed by 79/100 (79%) of participants. Of those that had surgery this equated to 79/89 (89%). Of those who had surgery, 100% had completed intraoperative data and 84/89 (94%) had post-operative data recorded at six weeks.

Compliance

Good compliance with oestrogen treatment (denoted as pessaries used at least >=75% of the expected time) was observed in 79% of participants pre-operatively (34/43), 83% at six weeks (35/42) and 71% at six months (20/28).

Clinical and patient-completed outcomes

Overall, scores from the PFDI-SF20 (POPDI-6 domain) were low at both 6 and 12 months, averaging 14.3 (SD: 16.4) and 15.6 (18.1) out of a maximum of 100, indicating a low level of prolapse-related symptoms at these times. Responses from the PFIQ-7 (POP-IQ-7 domain) were similarly low (Table 3). Scores appeared similar in both groups, but with expected high levels of uncertainty given the limited size of sample. The number of participants reporting being improved (very much better or better) on the PGI-I was 92% (73/79) at 6 months and

For peer review only - http://bmjopen.bmj.com/site/about/guidelines.xhtml

89% (70/79) at 12 months (Table 4). The number of participants with an objective rating of prolapse failure (from POP-Q) at 6 months was 22/55 (40%) (Table 5). Two repeat incontinence surgeries were recorded in the no treatment group over six months, with none in the oestrogen group.

Safety

More UTIs (8/42; 19% versus 4/42; 10%) - resulting in more antibiotics prescriptions (9/42; 21% versus 5/42; 12%) were reported in the 'no treatment' group than in the oestrogen group. Two serious adverse events were recorded in the oestrogen group - one woman with high temperature was admitted for intravenous antibiotics but culture of vaginal and urine samples were negative, and another woman was diagnosed with leukaemia. Neither were thought to be related to treatment. Two incidental hospitalisations were also recorded in the 'no treatment' group (one case of heavy bleeding thought to be unrelated to surgery and another with pancreatic cancer). No concerns were expressed by the independent oversight committee who met half way through the recruitment period to review the safety data.

Discussion

In this open label randomised study we sought to examine the feasibility of randomising post-menopausal women undergoing POP surgery to receive vaginal oestrogen. Our four key feasibility indicators were met and we have shown that a large multicentre randomised controlled trial (RCT) is feasible. It is possible to randomise and follow up patients with high fidelity over at least 12 months. Oestrogen treatment in the form of vaginal pessaries was well tolerated and consistently applied by most women. As this was a feasibility study with an open-label design, no inferences can be made about the treatment's therapeutic efficacy. However, the data does suggest that further research is warranted. There was evidence that the majority of women in both trial groups did report improvement in their prolapse symptoms, quality of life (QOL) and other aspects of urinary and sexual function. The effect was sustained for at least the first six months after surgery irrespective of oestrogen treatment.

For peer review only - http://bmjopen.bmj.com/site/about/guidelines.xhtml

Among women planning to undergo surgical repair for prolapse, three studies have reported on preoperative vaginal oestrogen by various methods, compared with placebo or no treatment, with a total of 111 participants (10-12). Follow up varied from 12 weeks to three years of postoperative surveillance. No vaginal oestrogen was given following surgery. The overall quality of evidence was poor. Use of vaginal oestrogen improved the vaginal maturation index, a histological measure of the status of the vaginal epithelium, at the time of surgery(13) and increased vaginal epithelial thickness(9) . Vaginal oestrogen application for six weeks preoperatively increased synthesis of mature collagen and increased thickness of the vaginal wall, suggesting this intervention improves both the substrate for suture placement at the time of repair and maintenance of tissue integrity of the pelvic floor(10) . In our study, we did not monitor the vaginal maturation index but we did note that there were fewer UTIs observed in the group that received vaginal oestrogen pre-operatively although the study was not powered for this outcome. The clinicians reported easier planes of dissection in women in the intervention group.

Mikkelsen et al described the three-year postoperative outcomes after preoperative treatment with vaginal oestradiol or placebo tablets before POP repair(11). They included a questionnaire of patient satisfaction, but no standardised or validated metric of prolapse symptoms, discontinuation rates and reasons were reported (10). They suggested that if vaginal oestrogen does improve tissue integrity then therapy may need to be continued postoperatively until the time of complete scar maturation (12). There is no clear evidence to state how long when a women would have adequate scar maturation after a prolapse surgery as this could be dependent on various factors of wound healing. In the current study women in the intervention group continued oestrogen up to 20 weeks post- surgery. The overall improvement in symptoms was comparable at 6 and 12 months post surgery in both groups. The majority of participants undergoing prolapse surgery in both groups demonstrated improvements in prolapse symptoms (90%) at 12 months, indicating that the benefit of

For peer review only - http://bmjopen.bmj.com/site/about/guidelines.xhtml

vaginal oestrogen may be marginal, with the majority of improvement in patient symptoms pertaining to surgery. This is hypothetical as there has been no RCT with long term follow up on these patients randomised to postoperative local oestrogen vs placebo or no treatment.

Surgical failures requiring repeat POP repairs on an average occur within 2 years after primary surgery(14). The PROSPECT study reported that 2% (6/395 standard repair) repeat surgeries were required as early as 1 year after primary surgery and 5% (16/348 standard repair) repeat surgery within 2 years(12). The authors concluded that follow up of patients for a minimum of 5 years would be required to assess recurrence of symptoms following POP surgery(14). We need to consider the pros and cons of long term maintenance treatment with vaginal oestrogen in the intervention group in a definitive study including cost, compliance, plateauing of benefits and regression of changes on stopping the treatment. The potential outcomes measured in long-term studies may include recurrence rates, interval of recurrence of prolapse symptoms from index surgery, reduction in urinary tract symptoms and sustained quality of life improvements and cost effectiveness analysis.

Strengths

This feasibility study was conducted over a period of 13 months during which the trial achieved its target recruitment of 100 women from 6 centres in the UK. The study efficiently met all the target criteria within the given time frame and showed that a substantial trial could be achieved.

We were able to capture patient responses by using validated PROMs such as the PFDI - SF20, PFIQ7 and PSIQ 12. The feasibility study has been reported in line with the CONSORT reporting guideline for feasibility/ pilot studies. The feasibility study has allowed the trial team to identify areas in the study process that require fine-tuning ahead of a larger trial. We are considering long term follow up of participants for a minimum of 5 years in order to capture the recurrence rates and relapse of POP symptoms after index surgery. In

For peer review only - http://bmjopen.bmj.com/site/about/guidelines.xhtml

addition, we are considering offering participants in the intervention group to continue with long-term maintenance therapy with vaginal oestrogen.

Limitations

Ideally we would have liked to conduct a RCT comparing vaginal oestrogen with a placebo pessary. All of the symptom related outcome measures are patient-completed and potentially prone to reporting bias if the participant is aware of the treatment allocation. However, despite extensive discussions with clinical trial suppliers, we were unable to procure a placebo without it being prohibitively expensive. Oestrogen pessaries are supplied in individually packaged, single-use plastic applicators, which cannot be replicated with a placebo due to the trademark on the packaging. Disassembling the pessary from the applicator to repackage in an unbranded applicator and container would require additional stability testing to confirm the bioavailability of the oestradiol hemihydrate. The manufacturer of Vagifem, Novo Nordisk, declined to provide assistance. Oestradiol is also available as a cream, and a placebo cream could be procured, but the acceptability and adherence to a cream as the route of treatment was not evaluated in this pilot and we should not extrapolate adherence based on pessaries.

A few patients post randomisation could not undergo surgery as they were deemed to be unfit. To avoid this from happening, the patients could have undergone pre-operative assessments to determine surgical and anaesthetic risk and randomised once they were scheduled for surgery. We could potentially have sent telephone, email or text reminders to patient to improve compliance with the post operative pessary use but it would not only be expensive but also not reflect a real life situation.

Conclusion

At this stage, we cannot expect that the outcomes of this pilot study to be directly translated into clinical care as the study was not large enough to be able to detect small to moderate

For peer review only - http://bmjopen.bmj.com/site/about/guidelines.xhtml

realistic-sized difference in rates of prolapse related QoL, nor was the scope wide enough in terms of the number of centres involved to return a generalisable result. However, this study is an important precursor to a larger, substantive trial and provides invaluable information that will help to ensure its success, which is ultimately needed to provide a definitive answer this important question.

Funding

The study was funded by a grant from the National Institute for Health Research for Patient Benefit (grant number: PB-PG-0213-30126). The funder had no role in study design, data collection, interpretation or analysis, or in writing the report for publication.

Acknowledgements

We thank the collaborators, women who recruited into this study, the healthcare professionals at the recruitment sites and the funders.

Miss Ranee Thakar, Croydon

Mrs. Preeti Jain, Walsall

Mr. Christian Phillips, Basingstoke

Mr. Jason Cooper, North Staffordshire

Mr. Athele Khunda, South Tees

Prof Jane Daniels and Lisa Leighton

Patient and Public involvement:

Rev. Ann Simons and Dr. Mary Evans

Trial Oversight committee:

Prof Shakila Thangaratinam, Valerie Morton, Tom Maishman and Deepali Sinha.

Declaration of interests

PML received sponsorship from Astellas, Contura and Pfizer to attend meetings.

For peer review only - http://bmjopen.bmj.com/site/about/guidelines.xhtml

For peer review only

All the other authors declare that they have no competing interests.

Sponsorship

The study sponsor was the Birmingham Women's and Children's NHS Foundation Trust. The sponsor had no role in study design, data collection, interpretation or analysis, or in writing the report for publication.

Authors' contributions

LJM, JPD and PML conceptualised the study as co- applicants. LM, JPD and PML contributed to the intervention development and design. PML oversaw the running of the trial and all the authors contributed to the on-going management of the trial. The LOTUS trial collaborative group collected data for the trial. LJM and VC developed the data analysis plan and summarised the results. TSV and LL evaluated the qualitative data. The manuscript was drafted by TSV with contributions from PML, LJM, JPD, VC and LL. All the authors contributed to the interpretation of the output and revised and reviewed the paper; they are the guarantors. All authors contributed to the writing of the final manuscript and provided critical comments during revisions. All authors approved the final version prior to submission. The Birmingham Clinical Trials Unit undertook the randomisation and data management and monitoring. The authors had full access to all the data from the study. The authors vouch for the accuracy and completeness of the data and analyses. All authors read and approved the final manuscript.

Data sharing statement

The dataset used in this manuscript is available on request from Birmingham Clinical Trials Unit.

For peer review only

For peer review only - http://bmjopen.bmj.com/site/about/guidelines.xhtml

Table 1: Baseline Characteristics

Characteristic		Oestrogen (n=50)	No Treatment (n=50)	Overall (n=100)
Age, years[1]	<65	21 (42%)	21 (42%)	42 (42%)
	>=65	29 (58%)	29 (58%)	58 (58%)
	Mean (SD)	65.7 (8.2)	65.9 (8.4)	65.8 (8.3)
Ethnic Group	White	45 (90%)	43 (86%)	88 (88%)
	Asian	2 (4%)	3 (6%)	5 (5%)
	Black	3 (6%)	1 (2%)	4 (4%)
	Mixed	0 (-)	2 (4%)	2 (5%)
	Other ethnic group	0 (-)	1 (2%)	1 (1%)
BMI (mg/k^2)	Mean (SD)	28.1 (5.1)	28.2 (5.9)	28.2 (5.5)
	Missing (N)	2	7	9
Parity[1]	<=2	27 (54%)	26 (52%)	53 (53%)
	>2	23 (46%)	24 (48%)	47 (47%)
Maximum Stage of Prolapse[1]	I	7 (14%)	7 (14%)	14 (14%)
	II	23 (46%)	23 (46%)	46 (46%)
	III/IV	20 (40%)	20 (40%)	40 (40%)
Concomitant continence surgery performed[1]	N (%)	2 (4%)	3 (6%)	5 (5%)
Previous operation for prolapse	N (%)	4 (8%)	4 (8%)	8 (8%)
Anterior	No. of repairs Median (IQR)	1 (1 - 1)	1 (1 - 1)	1 (1 - 1)
Posterior	No. of repairs Median (IQR)	1 (1 - 1)	1 (1 - 1)	1 (1 - 1)
Hysterectomy	N (%)	12 (24%)	8 (16%)	20 (20%)
Vaginal pessary/ring currently in place	N (%)	5 (10%)	8 (16%)	13 (13%)
Physiotherapy treatment for prolapse/urinary incontinence in last 12 months	N (%)	8 (16%)	10 (20%)	18 (18%)
Drug treatment for urinary incontinence	N (%)	0 (-)	4 (8%)	4 (4%)
Treatment for overactive bladder	N (%)	0 (-)	0 (-)	0 (-)

[1] minimisation variable

For peer review only - http://bmjopen.bmj.com/site/about/guidelines.xhtml
For peer review only

Table 2: Operative details

**Aspect of surgery**		**Oestrogen (n=44)**	**No Treatment (n=45)**
POP surgery performed	Anterior repair	35 (80%)	34 (76%)
	Posterior repair	16 (36%)	19 (42%)
	Vaginal hysterectomy +/- bilateral salping-oopherectomy	26 (59%)	25 (56%)
	Sacrospinous Fixation	6 (14%)	6 (13%)
Concomitant surgery	Sling/Tension-free vaginal tape	1 (2%)	1 (2%)
	Botulinum toxin A	1 (2%)	1 (2%)
	Suprapublic catheter	1 (2%)	1 (2%)
Ease of dissection	Very easy	6 (14%)	5 (11%)
	Easy	22 (50%)	13 (29%)
	Normal	14 (%)	21 (47%)
	Difficult	2 (5%)	5 (11%)
	Very difficult	0 (-)	1 (2%)
Blood loss (g)	N; Median (IQR)	N=44<br>63 (50 - 125)	N=44<br>54 (50 - 100)
	Missing	0 (-)	1 (2%)
Visceral injury	Bladder injury	0 (-)	0 (-)
	Urethral injury	0 (-)	0 (-)
	Ureteric injury	1 (2%)	0 (-)
	Bowel injury	0 (-)	0 (-)
	Button holing of vagina	3 (7%)	1 (2%)
Suture used – Fascial plication[1]	Polyglactin (Vicryl®, Ethicon/Polysorb®, Medtronic)	10 (23%)	10 (22%)
	Polydioxanone (PDS®-II, Ethicon)	35 (80%)	34 (76%)
	Poligelcarprine (Monocryl®, Ethicon)	0 (-)	0 (-)
	Missing	0	1
Suture used – Vagina[1]	Polyglactin (Vicryl®, Ethicon/Polysorb®, Medtronic)	41 (93%)	42 (93%)
	Polydioxanone (PDS®-II, Ethicon)	5 (11%)	6 (13%)
	Poligelcarprine (Monocryl®, Ethicon)	3 (7%)	1 (2%)
	Vaginal packing used	23 (52%)	28 (62%)
Cather inserted in theatre	Yes	43 (98%)	42 (93%)
Post-operative complications noted before discharge	Infection	1 (2%)	3 (7%)
	Ureteric injury	0 (-)	0 (-)
	Bladder injury	0 (-)	0 (-)
	Bowel injury	0 (-)	0 (-)
	Vascular injury	0 (-)	0 (-)
	Neurological injury	0 (-)	0 (-)
	Peri or post-operative blood transfusion	0 (-)	0 (-)
	Peri or post-operative thromboembolism	0 (-)	0 (-)

For peer review only

	Death	0 (-)	0 (-)

[1]More than one can be selected, hence why numbers do not add up to total.

For peer review only

Table 3: Results of patient reported outcomes – PFDI20, PFIQ-7 and PISQ-12

Outcome measure Timepoint	Oestrogen N; Mean (SD)	No Treatment N; Mean (SD)	Difference between groups (95% CI)[3]
PFDI-SF20; POPDI-6 Domain (0-100, higher=worse pain)[1]			
Baseline	N=48; 43.4 (24.8)	N=49; 46.0 (24.4)	
6 Month	N=38; 16.3 (20.3)	N=41; 12.5 (11.6)	4.1 (-3.9, 12.1)
12 Month	N=37 16.7 (20.6)	N=41 14.6 (15.6)	4.4 (-4.7, 13.5)
PFDI-SF20; CRAD-8 Domain (0-100, higher=worse pain)[1]			
Baseline	N=48; 26.0 (25.0)	N=48; 24.3 (18.0)	
6 Month	N=38; 12.7 (14.4)	N=41; 15.2 (13.2)	-2.4 (-7.6, 3.4)
12 Month	N=37 12.8 (14.8)	N=42 15.8 (15.8)	-1.7 (-8.0, 4.6)
PFDI-SF20; UDI-6 Domain (0-100, higher=worse pain)[1]			
Baseline	N=48; 34.8 (22.6)	N=49; 37.9 (26.7)	
6 Month	N=38; 16.4 (17.5)	N=41; 17.2 (18.5)	0.5 (-8.9, 7.9)
12 Month	N=36 20.3 (17.5)	N=42 21.5 (25.3)	0.7 (-9.1, 10.4)
PFDI-SF20; Summary Score (0-300, higher=worse pain)[1]			
Baseline	N=48; 104.3 (62.8)	N=48; 107.0 (57.4)	
6 Month	N=38; 45.4 (43.8)	N=41; 45.0 (37.1)	0.6 (-18.4, 17.2)
12 Month	N=36 49.8 (44.1)	N=41 51.1 (47.7)	4.8 (-16.2, 25.8)
PFIQ-7; UIQ-7 Domain (0-100, higher=worse condition)[1]			
Baseline	N=50; 21.0 (25.8)	N=50; 21.5 (24.6)	
6 Month	N=38; 9.6 (21.7)	N=41; 5.9 (11.2)	1.3 (-6.9, 9.5)
12 Month	N=37; 8.9 (16.8)	N=42; 8.0 (13.9)	1.4 (-6.0, 8.8)
PFIQ-7; CRAIQ-7 Domain (0-100, higher=worse condition)[1]			
Baseline	N=50; 12.0 (21.0)	N=50; 8.7 (13.5)	
6 Month	N=38; 4.9 (13.1)	N=41; 2.4 (6.1)	1.6 (-2.5, 5.8)
12 Month	N=36; 4.5 (16.3)	N=42; 2.9 (6.1)	2.2 (-2.8, 7.1)
PFIQ-7; POPIQ-7 Domain (0-100, higher=worse condition)[1]			
Baseline	N=50;	N=50;	

For peer review only - http://bmjopen.bmj.com/site/about/guidelines.xhtml

	17.7 (21.2)	17.1 (22.1)	
6 Month	N=38; 5.0 (13.4)	N=41; 2.3 (6.1)	2.5 (-2.5, 7.5)
12 Month	N=36; 1.9 (6.8)	N=42; 1.7 (5.2)	0.2 (-2.7, 3.1)
PFIQ-7; Summary Score (0-300, higher=worse pain)[1]			
Baseline	N=50; 50.8 (54.8)	N=50; 47.3 (51.9)	
6 Month	N=38; 19.5 (39.8)	N=41; 10.7 (19.8)	6.2 (-8.7, 21.2)
12 Month	N=36; 14.3 (29.1)	N=42; 12.7 (18.3)	4.5 (-6.8, 15.8)
PISQ-12 (0-48, higher=better sexual function)[2]			
Baseline	N=18; 31.2 (6.5)	N=19; 33.1 (7.3)	
6 Month	N=11; 33.1 (4.1)	N=14; 36.1 (5.7)	-2.4 (-7.0, 2.2)
12 Month	N=11; 34.5 (2.7)	N=10; 32.8 (5.1)	3.4 (0.6, 6.2)

[1]<0 indicates less pain/better condition failures with oestrogen

[2]>0 indicates better with oestrogen

[3]Differences are adjusted for baseline score and minimisation variables

For peer review only

For peer review only - http://bmjopen.bmj.com/site/about/guidelines.xhtml

Table 4: Results of patient reported outcomes – patient global impression of improvement (PGI-I)

Timepoint<br>*PGI-I response*		Oestrogen	No Treatment	Relative risk (95%CI)[3]
**6 Week**		N=42	N=42	
1 = Very much better	N (%)	22 (52%)	14 (33%)	
2 = Much better	N (%)	16 (38%)	18 (43%)	
3 = A little better	N (%)	4 (10%)	8 (19%)	
4 = No change	N (%)	0 (-)	2 (5%)	
5 = A little worse	N (%)	0 (-)	0 (-)	
6 = Much Worse	N (%)	0 (-)	0 (-)	
7 = Very Much Worse	N (%)	0 (-)	0 (-)	
Better score[1]	N (%)	42 (100%)	40 (95%)	1.05 (0.98 – 1.12)
Worse score[2]	N (%)	0 (-)	2 (5%)	
**6 Month**		N=38	N=41	
1 = Very much better	N (%)	13 (34%)	18 (44%)	
2 = Much better	N (%)	16 (42%)	17 (41%)	
3 = A little better	N (%)	4 (11%)	5 (12%)	
4 = No change	N (%)	3 (8%)	0 (-)	
5 = A little worse	N (%)	2 (5%)	1 (2%)	
6 = Much Worse	N (%)	0 (-)	0 (-)	
7 = Very Much Worse	N (%)	0 (-)	0 (-)	
Better score[1]	N (%)	33 (87%)	40 (98%)	0.89 (0.78 – 1.02)
Worse score[2]	N (%)	5 (13%)	1 (2%)	
**12 Month**		N=37	N=42	
1 = Very much better	N (%)	14 (38%)	17 (40%)	
2 = Much better	N (%)	15 (41%)	17 (40%)	
3 = A little better	N (%)	3 (8%)	4 (10%)	
4 = No change	N (%)	4 (11%)	2 (5%)	
5 = A little worse	N (%)	1 (3%)	0 (-)	
6 = Much Worse	N (%)	0 (-)	2 (5%)	
7 = Very Much Worse	N (%)	0 (-)	0 (-)	
Better score[1]	N (%)	32 (86%)	38 (90%)	0.96 (0.81 – 1.12)
Worse score[2]	N (%)	5 (14%)	4 (10%)	

[1]Better score is derived from scores 1 (Very much better) to 3 (A little better)
[2]Worse score is derived from scores 4 (No change) to 7 (Very much worse)
[3]>1 indicates better with oestrogen

For peer review only - http://bmjopen.bmj.com/site/about/guidelines.xhtml

Table 5: Results of POP-Q assessment

Anatomical site in vagina Timepoint	Oestrogen N; Mean (SD)	No Treatment N; Mean (SD)	Difference between groups (95% CI)
Aa (anterior wall), cm			
Baseline	N=49; 0.1 (1.9)	N=48; -0.3 (1.7)	
6 Months[1]	N=26; -1.7 (2.0)	N=29; -2.6 (2.0)	0.7 (0.1, 1.3)
Ba (anterior edge), cm			
Baseline	N=49; 0.2 (2.0)	N=48; 0.1 (1.8)	
6 Months[1]	N=25; -2.0 (2.0)	N=29; -2.9 (1.9)	0.6 (0.0, 1.2)
C (cervix/cuff), cm			
Baseline	N=49; -3.7 (2.5)	N=48; -3.4 (3.4)	
6 Months[1]	N=25; -2.9 (2.8)	N=29; -2.2 (3.6)	-0.2 (-1.3, 0.9)
Ap (posterior wall), cm			
Baseline	N=49; -1.2 (1.5)	N=48; -1.0 (1.7)	
6 Months[1]	N=26; -0.8 (1.7)	N=29; -1.0 (1.7)	0.5 (-0.2, 1.1)
Bp (posterior edge), cm			
Baseline	N=49; -1.1 (1.6)	N=48; -1.0 (1.8)	
6 Months[1]	N=26; -0.9 (1.8)	N=29; -1.0 (1.8)	0.4 (-0.3, 1.1)
D (posterior fornix), cm			
Baseline	N=39; -5.3 (1.9)	N=40; -5.1 (2.6)	
6 Months[1]	N=7; -1.3 (1.5)	N=4; -1.8 (1.9)	0.7 (-6.2, 7.5)
Total vaginal length, cm			
Baseline	N=49; 8.5 (1.1)	N=48; 8.0 (1.6)	
6 Months[2]	N=26; -0.2 (1.3)	N=29; -0.5 (1.7)	0.3 (0.4, 0.9)
Objective prolapse (any of Aa, Ba, C, Ap, Bp, or D>0) at six months			
	**N (%)**	**N (%)**	**Relative risk (95%CI)[3]**
Failure	9/26	13/29	0.77 (0.40 – 1.50)

[1]Change from baseline adjusted for baseline; difference <0 indicates better with oestrogen

[2]Adjusted for baseline length; difference<0 indicates better with oestrogen

[3]<1 indicates less prolapse failures with oestrogen

Figure1:Flow of participants through the trial

For peer review only - http://bmjopen.bmj.com/site/about/guidelines.xhtml

References

1.Haylen BT, MaherCF,Barber MD et al.An International Urogynecological Association (IUGA) / International Continence Society (ICS) joint report on the terminology for female pelvic organ prolapse (POP).*Int Urogynecol J.* 2016; 27 : 165-194

2. Nilsson K, Risberg B, Heimer G. The vaginal epithelium in the postmenopause--cytology, histology and pH as methods of assessment. Maturitas. 1995;21(1):51-6.
3.Ismail SI, Bain C, Hagen S. Oestrogens for treatment or prevention of pelvic organ prolapse in postmenopausal women. The Cochrane database of systematic reviews. 2010(9):CD007063.

4. Blatch-Jones AJ, Pek W, Kirkpatrick E, *et al.* Role of feasibility and pilot studies in randomised controlled trials: a cross-sectional study *BMJ Open* 2018;**8:**e022233. doi: 10.1136/bmjopen-2018-022233

5.Eldridge SM, Chan CL, Campbell MJ, Bond CM, Hopewell S, Thabane L, Lancaster GA; PAFS consensus group. CONSORT 2010 statement: extension to randomised pilot and feasibility trials. BMJ. 2016 Oct 24;355:i5239. PMID: 27777223

6.[Available from: https://www.birmingham.ac.uk/Documents/college-mds/trials/bctu/lotus/LOTUS-protocol-V2.0.pdf

7.Barber MD WM BR. Short forms of two condition-specific quality-of-life questionnaires for women with pelvic floor disorders (PFDI-20 and PFIQ-7). . Am J Obstet Gynecol 2005.

8. Rogers RG, Coates KW, Kammerer-Doak D, Khalsa S, Qualls C. A short form of the Pelvic Organ Prolapse/Urinary Incontinence Sexual Questionnaire (PISQ-12). Int Urogynecol J Pelvic Floor Dysfunct. 2003;14(3):164-8.

9. Srikrishna S, Robinson D, Cardozo L. Validation of the Patient Global Impression of Improvement (PGI-I) for urogenital prolapse. Int Urogynecol J. 2010;21(5):523-8.

10. Felding C, Mikkelsen AL, Clausen HV, Loft A, Larsen LG. Preoperative treatment with oestradiol in women scheduled for vaginal operation for genital prolapse. A randomised, double-blind trial. Maturitas. 1992;15(3):241-9.

11. Mikkelsen AL, Felding C, Clausen HV. Clinical effects of preoperative oestradiol treatment before vaginal repair operation. A double-blind, randomized trial. Gynecol Obstet Invest. 1995;40(2):125-8.

12. Vaccaro CM, Mutema GK, Fellner AN, Crisp CC, Estanol MV, Kleeman SD, et al. Histologic and cytologic effects of vaginal estrogen in women with pelvic organ prolapse: a randomized controlled trial. Female Pelvic Med Reconstr Surg. 2013;19(1):34-9.

13. Moalli PA, Shand SH, Zyczynski HM, Gordy SC, Meyn LA. Remodeling of vaginal connective tissue in patients with prolapse. Obstet Gynecol. 2005;106(5 Pt 1):953-63.

For peer review only - http://bmjopen.bmj.com/site/about/guidelines.xhtml

14. Glazener CM, Breeman S, Elders A, Hemming C, Cooper KG, Freeman RM, et al. Mesh, graft, or standard repair for women having primary transvaginal anterior or posterior compartment prolapse surgery: two parallel-group, multicentre, randomised, controlled trials (PROSPECT). Lancet. 2017;389(10067):381-92.

For peer review only

For peer review only - http://bmjopen.bmj.com/site/about/guidelines.xhtml

Figure 1: Flow of participants through the trial

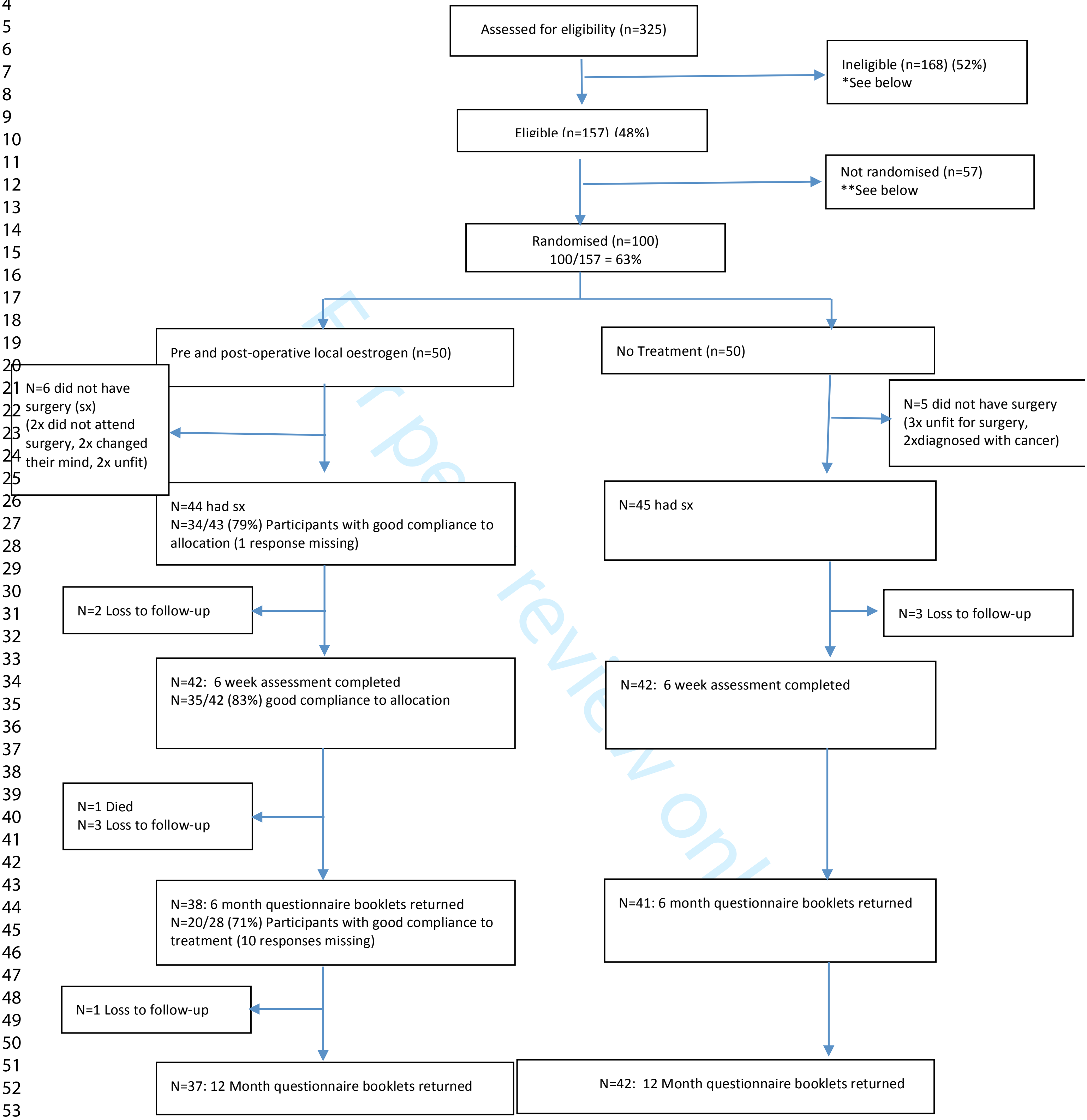

*Reasons for Ineligibility N=168 (%): No prolapse surgery required 38 (22), Recurrence of prolapse in the same compartment 32(19), Has had previous breast or uterine malignancy 20 (12), Undergoing HRT treatment 17 (10), Unable to understand English 16 (9), Genital bleeding of unknown origin 15 (9), Has had previous hormone-dependent neoplasms 13 (8), Previous POP surgery involving mesh 6 (3), Previous thrombo-embolic episode related to oestrogen therapy 4 (2), Two or more culture positive UTI episodes in the last 6 months 1 (<1), Other 6 (3)

**Reasons for Non Randomisation N=57 (%):Patient did not attend clinic 19 (33), Not enough time to discuss trial and gain informed consent 14 (25), Declined Consent/not willing to be randomised 9 (16), Not willing to wait 6 weeks for surgery 5 (9), Unable to gain consent 4 (7), Did not want to complete questionnaires, 1(2)

For peer review only - http://bmjopen.bmj.com/site/about/guidelines.xhtml

For peer review only

CONSORT 2010 checklist of information to include when reporting a randomised trial*

Section/Topic	Item No	Checklist item	Reported on page No
**Title and abstract**			
	1a	Identification as a randomised trial in the title	1
	1b	Structured summary of trial design, methods, results, and conclusions (for specific guidance see CONSORT for abstracts)	2
**Introduction**			
Background and objectives	2a	Scientific background and explanation of rationale	5
	2b	Specific objectives or hypotheses	5
**Methods**			
Trial design	3a	Description of trial design (such as parallel, factorial) including allocation ratio	6
	3b	Important changes to methods after trial commencement (such as eligibility criteria), with reasons	7
Participants	4a	Eligibility criteria for participants	7
	4b	Settings and locations where the data were collected	7
Interventions	5	The interventions for each group with sufficient details to allow replication, including how and when they were actually administered	8
Outcomes	6a	Completely defined pre-specified primary and secondary outcome measures, including how and when they were assessed	8
	6b	Any changes to trial outcomes after the trial commenced, with reasons	8
Sample size	7a	How sample size was determined	9
	7b	When applicable, explanation of any interim analyses and stopping guidelines	9
Randomisation:			
Sequence generation	8a	Method used to generate the random allocation sequence	7
	8b	Type of randomisation; details of any restriction (such as blocking and block size)	7
Allocation concealment mechanism	9	Mechanism used to implement the random allocation sequence (such as sequentially numbered containers), describing any steps taken to conceal the sequence until interventions were assigned	7
Implementation	10	Who generated the random allocation sequence, who enrolled participants, and who assigned participants to interventions	7
Blinding	11a	If done, who was blinded after assignment to interventions (for example, participants, care providers, those	-

For peer review only - http://bmjopen.bmj.com/site/about/guidelines.xhtml

		assessing outcomes) and how	
	11b	If relevant, description of the similarity of interventions	-
Statistical methods	12a	Statistical methods used to compare groups for primary and secondary outcomes	9
	12b	Methods for additional analyses, such as subgroup analyses and adjusted analyses	9
**Results**			
Participant flow (a diagram is strongly recommended)	13a	For each group, the numbers of participants who were randomly assigned, received intended treatment, and were analysed for the primary outcome	10
	13b	For each group, losses and exclusions after randomisation, together with reasons	11
Recruitment	14a	Dates defining the periods of recruitment and follow-up	11
	14b	Why the trial ended or was stopped	
Baseline data	15	A table showing baseline demographic and clinical characteristics for each group	10
Numbers analysed	16	For each group, number of participants (denominator) included in each analysis and whether the analysis was by original assigned groups	10-11
Outcomes and estimation	17a	For each primary and secondary outcome, results for each group, and the estimated effect size and its precision (such as 95% confidence interval)	9-11
	17b	For binary outcomes, presentation of both absolute and relative effect sizes is recommended	10-11
Ancillary analyses	18	Results of any other analyses performed, including subgroup analyses and adjusted analyses, distinguishing pre-specified from exploratory	
Harms	19	All important harms or unintended effects in each group (for specific guidance see CONSORT for harms)	11
**Discussion**			
Limitations	20	Trial limitations, addressing sources of potential bias, imprecision, and, if relevant, multiplicity of analyses	14
Generalisability	21	Generalisability (external validity, applicability) of the trial findings	12-14
Interpretation	22	Interpretation consistent with results, balancing benefits and harms, and considering other relevant evidence	12-14
**Other information**			
Registration	23	Registration number and name of trial registry	1-2
Protocol	24	Where the full trial protocol can be accessed, if available	7
Funding	25	Sources of funding and other support (such as supply of drugs), role of funders	15

*We strongly recommend reading this statement in conjunction with the CONSORT 2010 Explanation and Elaboration for important clarifications on all the items. If relevant, we also recommend reading CONSORT extensions for cluster randomised trials, non-inferiority and equivalence trials, non-pharmacological treatments, herbal interventions, and pragmatic trials. Additional extensions are forthcoming: for those and for up to date references relevant to this checklist, see www.consort-statement.org.

For peer review only - http://bmjopen.bmj.com/site/about/guidelines.xhtml

BMJ Open

A Randomised Controlled Trial to investigate the effectiveness of Local Oestrogen Treatment in Postmenopausal Women Undergoing Pelvic Organ Prolapse Surgery (LOTUS) – a pilot study to assess feasibility of a large multi-centre trial

Journal:	*BMJ Open*
Manuscript ID	bmjopen-2018-025141.R3
Article Type:	Original research
Date Submitted by the Author:	21-May-2020

Complete List of Authors:	Verghese, Tina; University of Birmingham, Institute of Metabolism and System Research<br>Middleton, Lee; University of Birmingham, School of Health and Population Sciences<br>Cheed, Versha; University of Birmingham, Birmingham Clinical Trials Unit<br>Leighton, Lisa; University of Birmingham, Birmingham Clinical Trials Unit<br>Daniels, Jane; University of Nottingham, Nottingham Clinical Trials Unit; University of Nottingham Clinical Trials Unit<br>Latthe, Pallavi; Birmingham Women's and Children's NHS Foundation Trust, Obstetrics and Gynaecology

**Primary Subject Heading**:	Obstetrics and gynaecology
Secondary Subject Heading:	Health services research, Patient-centred medicine, Research methods
Keywords:	pilot, feasibility, pelvic organ prolapse, postmenopausal, recurrence, oestrogen

SCHOLARONE™
Manuscripts

BMJ

I, the Submitting Author has the right to grant and does grant on behalf of all authors of the Work (as defined in the below author licence), an exclusive licence and/or a non-exclusive licence for contributions from authors who are: i) UK Crown employees; ii) where BMJ has agreed a CC-BY licence shall apply, and/or iii) in accordance with the terms applicable for US Federal Government officers or employees acting as part of their official duties; on a worldwide, perpetual, irrevocable, royalty-free basis to BMJ Publishing Group Ltd ("BMJ") its licensees and where the relevant Journal is co-owned by BMJ to the co-owners of the Journal, to publish the Work in this journal and any other BMJ products and to exploit all rights, as set out in our licence.

The Submitting Author accepts and understands that any supply made under these terms is made by BMJ to the Submitting Author unless you are acting as an employee on behalf of your employer or a postgraduate student of an affiliated institution which is paying any applicable article publishing charge ("APC") for Open Access articles. Where the Submitting Author wishes to make the Work available on an Open Access basis (and intends to pay the relevant APC), the terms of reuse of such Open Access shall be governed by a Creative Commons licence – details of these licences and which Creative Commons licence will apply to this Work are set out in our licence referred to above.

Other than as permitted in any relevant BMJ Author's Self Archiving Policies, I confirm this Work has not been accepted for publication elsewhere, is not being considered for publication elsewhere and does not duplicate material already published. I confirm all authors consent to publication of this Work and authorise the granting of this licence.

For peer review only

A Randomised Controlled Trial to investigate the effectiveness of Local Oestrogen Treatment in Postmenopausal Women Undergoing Pelvic Organ Prolapse Surgery (LOTUS) – a pilot study to assess feasibility of a large multi-centre trial

For peer review only

Tina Sara Verghese MS MRCOG, Clinical Research Fellow, Institute of Metabolism and System Research College of Medical & Dental Sciences, University of Birmingham, B15 2TT, UK; t.s.verghese@bham.ac.uk

Lee Jonathan Middleton, MSc, Senior Medical Statistician, Birmingham Clinical Trials Unit, University of Birmingham, B15 2TT, UK; l.j.middleton@bham.ac.uk

Versha Cheed, MSc, Medical Statistician, Birmingham Clinical Trials Unit, University of Birmingham, B15 2TT, UK; solankiv@adf.bham.ac.uk

Lisa Leighton LOTUS Trial Co-ordinator, Birmingham Clinical Trials Unit, University of Birmingham, B15 2TT, UK; leightlj@adf.bham.ac.uk

Jane P Daniels PhD MSc MMedSci BSc, Professor of Clinical Trials, Faculty of Medicine & Health Sciences, University of Nottingham, NG7 2UH,UK; jane.daniels@nottingham.ac.uk

Pallavi Manish Latthe MD FRCOG DFFP, Consultant Urogynaecologist, Birmingham Women's and Children's NHS Foundation Trust, B15 2TG, UK; pallavi.latthe@bwnft.nhs.uk

on behalf of the **LOTUS trial collaborative group**

Corresponding author:

E-mail: t.s.verghese@bham.ac.uk

Formatted for BMJ open

Abstract: 275

Main body: 3802

For peer review only

For peer review only - http://bmjopen.bmj.com/site/about/guidelines.xhtml

Abstract

Objective: To evaluate the feasibility of a multicentre randomised controlled trial (RCT) comparing oestrogen treatment with no oestrogen supplementation in women undergoing pelvic organ prolapse (POP) surgery.

Design and setting: A randomised, parallel, open, external pilot trial involving six UK urogynaecology centres (July 2015- August 2016).

Participants: Post-menopausal women with POP opting for surgery, unless involving mesh or for recurrent POP in same compartment.

Intervention: Women were randomised (1:1) to pre and post-operative oestrogen or no treatment. Oestrogen treatment (oestradiol hemihydrate 10mcg vaginal pessaries) commenced 6 weeks prior to surgery (once daily for two weeks, twice weekly for four weeks) and twice weekly for 26 weeks from 6 weeks post-surgery.

Outcome measures: The main outcomes were assessment of eligibility and recruitment rates along with compliance and data completion. To obtain estimates for important aspects of the protocol to allow development of a definitive trial.

Results: 325 women seeking POP surgery were screened over 13 months and 157 (48%) were eligible. Of these, 100 (64%) were randomised, 50 to oestrogen and 50 to no oestrogen treatment, with 89 (44/45 respectively) ultimately having surgery. Of these, 89% (79/89) returned complete questionnaires at six months and 78% (32/41) reported good compliance with oestrogen. No serious adverse events were attributable to oestrogen use.

For peer review only - http://bmjopen.bmj.com/site/about/guidelines.xhtml

Conclusions: A large multicentre RCT of oestrogen versus no treatment is feasible, as it is possible to randomise and follow up participants with high fidelity. Four predefined feasibility criteria were met. Compliance with treatment regimens is not a barrier. A larger trial is required to definitively address the role of peri-operative oestrogen supplementation.

Trial registration number: ISRCTN46661996.

Keywords

Pilot, feasibility, pelvic organ prolapse, postmenopausal, recurrence, oestrogen.

For peer review only

For peer review only - http://bmjopen.bmj.com/site/about/guidelines.xhtml

Article Summary

Strengths and limitations of this study

- This randomised, external, pilot trial had pre-planned feasibility thresholds to judge to assess whether a large trial is feasible.
- The data on number of women at each stage of the trial (screening, randomisation, using the intervention and completing outcome assessments at two follow-up time-points) were collected to estimate recruitment, retention and compliance rates.
- There was the potential for post-randomisation exclusions of participants who were ultimately considered unfit for surgery.
- Blinding of the oestrogen pessary was not possible, so reported outcomes could be subject to responder bias.
- The trial was not designed to be large or long enough to provide convincing evidence for use of pre and post-operative oestrogen treatment.

Trial funding

This work was funded by UK National Institute for Health Research, Research for Patient Benefit programme (grant reference PB-PG-0213-30126)

Declaration of interests

PML received sponsorship from Astellas, Contura and Pfizer to attend meetings.

All the other authors declare that they have no competing interests.

For peer review only

Introduction

Pelvic organ prolapse (POP) is the descent of one or more of the anterior vaginal wall, posterior vaginal wall, the uterus (cervix), or the apex of the vagina (after hysterectomy)(1). This definition is based on anatomical change. However, the diagnosis of prolapse should start with symptoms and relate these symptoms to the descent of pelvic organs(1). Women with symptomatic POP present with a sense of vaginal bulge, in association with a variety of urinary, bowel and sexual symptoms. In postmenopausal women lack of oestrogen is hypothesised to weaken the pelvic floor muscles(2). Whilst POP surgery is effective, with symptom improvement in 70-88% of women, the risk of recurrent prolapse is high, estimated at 10-30%(1). Efforts to reduce perioperative complications of POP surgery and enhancement of long-term cure are key priorities for the future management of a rapidly ageing population. In postmenopausal women with vaginal atrophy, surgical dissection may be difficult due to thinning of the vaginal walls. In addition, they may be at a higher risk of surgical wound infection, secondary to changes in the vaginal flora. Oestrogen treatment is often used, on its own or in conjunction with various treatment modalities, for vaginal atrophy(3) . However, despite frequent use, there are no robust data on the benefits of pre and postoperative oestrogen treatment in postmenopausal women undergoing POP surgery.

A Cochrane review by Ismail et al, published in 2010 did not find any clear evidence to suggest whether oestrogens for the prevention and treatment of POP and concluded an adequately powered RCT with long term follow up is needed to identify the benefits or risks associated with oestrogen supplementation, including before and after POP surgery(3).

There is a plausible argument for using low-dose oestrogen to improve the vaginal environment and reduce infections at the time of prolapse surgery, but there is little evidence to support its effect on the quality of surgical repair, prolapse cure rates or recurrence.

For peer review only - http://bmjopen.bmj.com/site/about/guidelines.xhtml

Furthermore, there is no information about any effect in terms of prolapse related symptoms, overall quality of life (QoL) and outcomes important to women. Finally, the duration of oestrogen treatment, and cost-effectiveness compared with current practice are unknown.

There were over 21,000 pelvic floor repair surgeries performed in England in 2014 on women with an average age of 61 years and incurring a hospital stay of 2 days (HES data, Q08.9, P23.1). These data mask the impact of surgical complications on resource use and quality of life, which could be mitigated if perioperative oestrogen was found to be clinically and cost-effective. Moreover, if this use reduces failure and recurrence rates, this would save cost.

Feasibility studies serve to address the question "can this study be done", by assessing uncertainty in key trial parameters, whilst pilot studies are a small scale version of the definitive study design to test whether all components can work together. There is often overlap between the objectives of any preparatory studies, as the ultimate aim is to maximise the success of a definitive study by identifying problems with recruitment and data collection processes(4). The aim of the feasibility study was to find out if an appropriately powered randomised controlled trial (RCT) can be realistically undertaken. The overall aim of this research is to determine whether pre and postoperative local oestrogen treatment is more effective in improving prolapse related patient reported outcomes and reducing recurrence of prolapse symptoms when compared to no treatment. To answer this question a large, multi-centre RCT is required. We report this study as per the CONSORT checklist for reporting a pilot trial (5)

Methods

We conducted a randomised parallel group open external pilot trial of pre and post-operative oestrogen treatment versus no treatment in women undergoing POP surgery. An external pilot is a rehearsal of the definitive study where the outcome data are not included as part of the main trial outcome data set. The study received a favourable ethical opinion from NHS

For peer review only - http://bmjopen.bmj.com/site/about/guidelines.xhtml

Research Ethics Committee (NRES Committee West Midlands, REC number 15/WM/0092) and was registered with a primary clinical trials registry (ISRCTN 46661996).

Population

Eligible participants were postmenopausal women who were having POP surgery without use of vaginal mesh. Participants were excluded if they had any of the following: hormone replacement therapy in the last 12 months; previous breast/uterine malignancy or other hormone-dependant neoplasms; genital bleeding of unknown origin; previous thrombo-embolic episodes in relation to oestrogen; known allergy to oestrogens; two or more episodes of culture positive urinary tract infection (UTI) in the last 6 months; voiding dysfunction; previous POP surgery involving mesh or previous POP surgery in the same compartment.

Study conduct and randomisation

All patients thought to fulfil the eligibility criteria were approached with information regarding the trial. Participants were identified in urogynaecology clinics, provided with a patient information sheet and given the opportunity to consider participation.

Once eligibility was confirmed, the trial team, which comprised of urogynaecologists and research nurses from district general hospitals as well as university hospitals across United Kingdom. They conducted face to face sessions with the participants in order to obtain written informed consent and baseline data. Women who declined trial participation remained eligible to participate in the qualitative study. Randomisation was performed using a web-based central randomisation system (via Birmingham Clinical Trials Unit) to allocate patients to either oestrogen or no treatment in a 1:1 ratio. Minimisation was used to achieve balance between age (<65 years or ≥65 years), parity (<=2 or >2 vaginal births), maximum stage of prolapse (I, II or III/IV) and whether a concomitant continence surgery was planned. An oversight committee was formed to provide independent guidance to the Trial Management Committee and to review accruing safety information during the period of recruitment.

For peer review only - http://bmjopen.bmj.com/site/about/guidelines.xhtml

Interventions

Those allocated oestrogen (oestradiol hemihydrate 10mcg vaginal pessaries; Vagifem™, Novo Nordisk) were instructed to use the oestrogen pessaries six weeks prior to surgery (once daily for two weeks and twice weekly for four weeks) up to the night before surgery. The treatment was restarted 6 weeks post-operatively, administering twice weekly for 20 weeks(6)Patient group discussion expressed that they felt they would be confident to insert an intra vaginal pessary 6 weeks after surgery and not earlier as they were worried about disturbing the healing process.

Women were encouraged to insert the pessaries into the vagina at the same time of day. However, if a dose was missed patients were advised it should be administered as soon as possible thereafter, provided the next dose was not due. Participants allocated no treatment received the usual care of the randomising centre. The surgical approach to POP repair was at the discretion of the urogynaecological surgeon. It was not possible to blind clinicians or participants due to the nature of the interventions.

Feasibility outcomes

Primary outcome measure of the feasibility study

To obtain estimates for important aspects of the protocol to allow development of a definitive trial.

Secondary outcome measure of the feasibility study

1. Assessment of effectiveness of patient identification and screening processes
2. Assessment of the effectiveness of the randomisation process of patients
3. Evaluation of robustness of data collection processes
4. The proportion of patients followed up at six months
5. Derivation of the preliminary data from clinical outcome measures (e.g.PFDI-SF20) to inform the sample size calculation for the substantive study

For peer review only - http://bmjopen.bmj.com/site/about/guidelines.xhtml

Clinical outcomes

Baseline data including clinical history and symptom-related questionnaires were recorded at the pre-surgery clinic appointment. Intraoperative, post-operative (six weeks) and six months post-surgery data including objective assessment of prolapse (POP quantification system [POP-Q]), complications (including urinary tract and vaginal infections) and repeat surgery information were collected by the randomising and/or operating clinician at scheduled appointments. The patient-reported questionnaires were: the Pelvic Floor Distress Inventory Short Form 20 (PFDI-SF20)(7), which comprises of three sub-domains (Pelvic Organ Prolapse Distress Inventory [POPDI-6], Colorectal-Anal Distress Inventory [CRADI-8], Urinary Distress Inventory [UDI-6]); the Pelvic Floor Impact Questionnaire (PFIQ-7), which also has three sub-domains (Pelvic Organ Prolapse Impact [POPIQ-7], Colorectal-Anal Impact [CRAIQ-7], Urinary Impact [UIQ-7]); the Pelvic Organ Prolapse/Urinary Incontinence Sexual Questionnaire (PISQ-12)(8) and Patient Global Impression of Improvement (PGI-I)(9). Questionnaires were collected at baseline then at six (at the scheduled appointment) and twelve months, by postal questionnaire. Serious adverse events, reported by clinical staff, were collected throughout the duration of the study.

Sample size

We planned to randomise 100 women; this number would allow us to measure recruitment and compliance rates with 95% confidence interval (CI) width between 10 and 20%. It would also be enough women to estimate the standard deviation (SD) of POPDI-6 with reasonable confidence for future planning of a larger trial (95% CI for SD would be 7 points, assuming the SD is around 20).

Statistical analysis

Feasibility outcomes were considered with simple summary statistics, with uncertainty estimates provided by 95% confidence intervals. Clinical and patient reported outcome measures (PROMs) were analysed with point estimates (RR or mean difference) and 95%

For peer review only - http://bmjopen.bmj.com/site/about/guidelines.xhtml

confidence intervals, adjusting for the minimisation variables. This was performed using SAS software, version 9.4 (SAS Institute).

They were not subject to hypothesis testing as the size of the sample would not allow reliable assessment of the effect of the interventions. Participants were considered in the groups they were randomised to regardless of compliance in these summaries (intention-to-treat). The dataset used in this manuscript is available on request from Birmingham Clinical Trials Unit.

Pilot outcomes

The following outcomes and targets were set a-priori as being indicative that a larger trial would be feasible to conduct. These were: i) patient eligibility rate (the proportion of screened patients eligible) at least 25%; ii) patient recruitment rate (the proportion of eligible patients randomised) at least 25%; iii) compliance rate (the proportion of patients with good compliance to treatment, i.e. at least 75% allocated pessaries used): at least 50%; iv) data completion rate (number of follow-up questionnaires completed at six months): at least 75%.

Patient and Public involvement

Throughout the process of the development and the running of the feasibility study, the study team have actively involved participation from patients and public in order to refine the trial and make the study about postmenopausal women suffering from symptoms of pelvic organ prolapse (POP). The research team conducted a qualitative study to identify the motivations for, and barriers to recruitment and participation in LOTUS. Twenty women were interviewed which provided key insight into trial recruitment barriers. This application benefited from the qualitative study in several ways (e.g. approaching patients and the acceptability of the investigational medicinal product).

Focus group interviews were held through which the research team gained insight from women who were given the opportunity to express their views and felt that further research in this area is much needed

For peer review only

Results

Patients and follow-up

Recruitment took place over a 13-month period between July 2015 and August 2016 in six urogynaecology centres in the UK (Birmingham Women's Hospital, Croydon University Hospital, Basingstoke and North Hampshire Hospital, Walsall Manor Hospital, Royal Stoke University Hospital, James Cook University Hospital, Middlesbrough). Once the target sample size was reached, the study stopped randomising women.

Of the women who presented with prolapse and wanted surgery, 325 consecutive women were screened for eligibility, 157 were found to be eligible (48%, 95%CI: 40%-56%) and 100 (64%, 95% CI 56-72%) of those eligible were randomised (Figure 1). The minimisation algorithm provide appropriate balance for the minimisation factors; the average age of participants was 66 years with 47% having parity greater than two with an average BMI of 28.2. The maximum stage of prolapse was III or IV (increased severity) for 40% of participants (Table 1).

Of the 100 randomised women, 89 ultimately had surgery (details given in Table 2). Of the other 11, 5 could not have surgery due to health issues, 2 changed their mind about surgery, 2 did not attend their surgical appointment and 2 were diagnosed with cancer. Six month follow-up questionnaires were completed by 79/100 (79%) of participants. Of those that had surgery this equated to 79/89 (89%). Of those who had surgery, 100% had completed intraoperative data and 84/89 (94%) had post-operative data recorded at six weeks.

Compliance

Good compliance with oestrogen treatment (denoted as pessaries used at least >=75% of the expected time) was observed in 79% of participants pre-operatively (34/43), 83% at six weeks (35/42) and 71% at six months (20/28).

For peer review only - http://bmjopen.bmj.com/site/about/guidelines.xhtml

Clinical and patient-completed outcomes

Overall, scores from the PFDI-SF20 (POPDI-6 domain) were low at both 6 and 12 months, averaging 14.3 (SD: 16.4) and 15.6 (18.1) out of a maximum of 100, indicating a low level of prolapse-related symptoms at these times. Responses from the PFIQ-7 (POP-IQ-7 domain) were similarly low (Table 3). Scores appeared similar in both groups, but with expected high levels of uncertainty given the limited size of sample. The number of participants reporting being improved (very much better or better) on the PGI-I was 92% (73/79) at 6 months and 89% (70/79) at 12 months (Table 4). The number of participants with an objective rating of prolapse failure (from POP-Q) at 6 months was 22/55 (40%) (Table 5). Two repeat incontinence surgeries were recorded in the no treatment group over six months, with none in the oestrogen group.

Safety

More UTIs (8/42; 19% versus 4/42; 10%) - resulting in more antibiotics prescriptions (9/42; 21% versus 5/42; 12%) were reported in the 'no treatment' group than in the oestrogen group. Two serious adverse events were recorded in the oestrogen group - one woman with high temperature was admitted for intravenous antibiotics but culture of vaginal and urine samples were negative, and another woman was diagnosed with leukaemia. Neither were thought to be related to treatment. Two incidental hospitalisations were also recorded in the 'no treatment' group (one case of heavy bleeding thought to be unrelated to surgery and another with pancreatic cancer). No concerns were expressed by the independent oversight committee who met half way through the recruitment period to review the safety data.

Discussion

In this open label randomised study, we sought to examine the feasibility of randomising post-menopausal women undergoing POP surgery to receive vaginal oestrogen. Our four key feasibility indicators were met and we have shown that a large multicentre randomised

For peer review only - http://bmjopen.bmj.com/site/about/guidelines.xhtml

controlled trial (RCT) is feasible. It is possible to randomise and follow up patients with high fidelity over at least 12 months. Oestrogen treatment in the form of vaginal pessaries was well tolerated and consistently applied by most women. As this was a feasibility study with an open-label design, no inferences can be made about the treatment's therapeutic efficacy. However, the data does suggest that further research is warranted. There was evidence that the majority of women in both trial groups did report improvement in their prolapse symptoms, quality of life (QOL) and other aspects of urinary and sexual function. The effect was sustained for at least the first six months after surgery irrespective of oestrogen treatment.

Among women planning to undergo surgical repair for prolapse, three studies have reported on preoperative vaginal oestrogen by various methods, compared with placebo or no treatment, with a total of 111 participants (10-12). Follow up varied from 12 weeks to three years of postoperative surveillance. No vaginal oestrogen was given following surgery. The overall quality of evidence was poor. Use of vaginal oestrogen improved the vaginal maturation index, a histological measure of the status of the vaginal epithelium, at the time of surgery(13) and increased vaginal epithelial thickness(9) . Vaginal oestrogen application for six weeks preoperatively increased synthesis of mature collagen and increased thickness of the vaginal wall, suggesting this intervention improves both the substrate for suture placement at the time of repair and maintenance of tissue integrity of the pelvic floor(10) . In our study, we did not monitor the vaginal maturation index but we did note that there were fewer UTIs observed in the group that received vaginal oestrogen pre-operatively although the study was not powered for this outcome. The clinicians reported easier planes of dissection in women in the intervention group.

Mikkelsen et al described the three-year postoperative outcomes after preoperative treatment with vaginal oestradiol or placebo tablets before POP repair(11). They included a questionnaire of patient satisfaction, but no standardised or validated metric of prolapse symptoms, discontinuation rates and reasons were reported(10). They suggested that if

For peer review only - http://bmjopen.bmj.com/site/about/guidelines.xhtml

vaginal oestrogen does improve tissue integrity then therapy may need to be continued postoperatively until the time of complete scar maturation (12). There is no clear evidence to state how long when a women would have adequate scar maturation after a prolapse surgery as this could be dependent on various factors of wound healing. In the current study women in the intervention group continued oestrogen up to 20 weeks post- surgery. The overall improvement in symptoms was comparable at 6 and 12 months post surgery in both groups. The majority of participants undergoing prolapse surgery in both groups demonstrated improvements in prolapse symptoms (90%) at 12 months, indicating that the benefit of vaginal oestrogen may be marginal, with the majority of improvement in patient symptoms pertaining to surgery. This is hypothetical as there has been no RCT with long term follow up on these patients randomised to postoperative local oestrogen vs placebo or no treatment. Karp et al found that early administration of vaginal estrogen after vaginal surgery via an estradiol-releasing ring is feasible and results in improved markers of tissue quality postoperatively compared to placebo and controls (14).

Surgical failures requiring repeat POP repairs on an average occur within 2 years after primary surgery (12). The PROSPECT study reported that 2% (6/395 standard repair) repeat surgeries were required as early as 1 year after primary surgery and 5% (16/348 standard repair) repeat surgery within 2 years(15). The authors concluded that follow up of patients for a minimum of 5 years would be required to assess recurrence of symptoms following POP surgery(15). We need to consider the pros and cons of long term maintenance treatment with vaginal oestrogen in the intervention group in a definitive study including cost, compliance, plateauing of benefits and regression of changes on stopping the treatment. The potential outcomes measured in long-term studies may include recurrence rates, interval of recurrence of prolapse symptoms from index surgery, reduction in urinary tract symptoms and sustained quality of life improvements and cost effectiveness analysis.

For peer review only - http://bmjopen.bmj.com/site/about/guidelines.xhtml

Strengths

This feasibility study was conducted over a period of 13 months during which the trial achieved its target recruitment of 100 women from 6 centres in the UK. The study met all the target criteria within the given time frame and showed that a substantial trial could be achieved.

We were able to capture patient responses by using validated PROMs such as the PFDI - SF20, PFIQ7 and PSIQ 12. The feasibility study has been reported in line with the CONSORT reporting guideline for feasibility/ pilot studies. The feasibility study has allowed the trial team to identify areas in the study process that require fine-tuning ahead of a larger trial. We are considering long term follow up of participants for a minimum of 5 years in order to capture the recurrence rates and relapse of POP symptoms after index surgery. In addition, we are considering offering participants in the intervention group to continue with long-term maintenance therapy with vaginal oestrogen.

Limitations

Ideally we would have liked to conduct a RCT comparing vaginal oestrogen with a placebo pessary. All of the symptom related outcome measures are patient-completed and potentially prone to reporting bias if the participant is aware of the treatment allocation. However, despite extensive discussions with clinical trial suppliers, we were unable to procure a placebo, without it being prohibitively expensive. Oestrogen pessaries are supplied in individually packaged, single-use plastic applicators, which cannot be replicated with a placebo due to the trademark on the packaging. Disassembling the pessary from the applicator to repackage in an unbranded applicator and container would require additional stability testing to confirm the bioavailability of the oestradiol hemihydrate. The manufacturer of Vagifem, Novo Nordisk, declined to provide assistance. Oestradiol is also available as a cream, and a placebo cream could be procured, but the acceptability and adherence to a cream

For peer review only - http://bmjopen.bmj.com/site/about/guidelines.xhtml

as the route of treatment was not evaluated in this pilot and we should not extrapolate adherence based on pessaries.

A few patients post randomisation could not undergo surgery as they were deemed to be unfit. To avoid this from happening, the patients could have undergone pre-operative assessments to determine surgical and anaesthetic risk and randomised once they were scheduled for surgery. We could potentially have sent telephone, email or text reminders to patient to improve compliance with the post operative pessary use but it would not only be expensive but also not reflect a real life situation.

Conclusion

At this stage, we cannot expect that the outcomes of this pilot study to be directly translated into clinical care as the study was not large enough to be able to detect small to moderate realistic-sized difference in rates of prolapse related QoL, nor was the scope wide enough in terms of the number of centres involved to return a generalisable result. However, this study is an important precursor to a larger, substantive trial and provides invaluable information that will help to ensure its success, which is ultimately needed to provide a definitive answer this important question.

Funding

The study was funded by a grant from the National Institute for Health Research for Patient Benefit (grant number: PB-PG-0213-30126). The funder had no role in study design, data collection, interpretation or analysis, or in writing the report for publication.

Acknowledgements

We thank the collaborators, women who recruited into this study, the healthcare professionals at the recruitment sites and the funders.

For peer review only - http://bmjopen.bmj.com/site/about/guidelines.xhtml

Miss Ranee Thakar, Croydon

Mrs. Preeti Jain, Walsall

Mr. Christian Phillips, Basingstoke

Mr. Jason Cooper, North Staffordshire

Mr. Athele Khunda, South Tees

Prof Jane Daniels and Lisa Leighton

Patient and Public involvement:

Rev. Ann Simons and Dr. Mary Evans

Trial Oversight committee:

Prof Shakila Thangaratinam, Valerie Morton, Tom Maishman and Deepali Sinha.

Declaration of interests

PML received sponsorship from Astellas, Contura and Pfizer to attend meetings.

All the other authors declare that they have no competing interests.

Sponsorship

The study sponsor was the Birmingham Women's and Children's NHS Foundation Trust. The sponsor had no role in study design, data collection, interpretation or analysis, or in writing the report for publication.

Authors' contributions

LJM, JPD and PML conceptualised the study as co- applicants. LJM, JPD and PML contributed to the intervention development and design. PML oversaw the running of the trial and all the authors contributed to the on-going management of the trial. The LOTUS trial collaborative group collected data for the trial. LJM and VC developed the data analysis plan and summarised the results. TSV and LL evaluated the qualitative data. The manuscript was drafted by TSV with contributions from PML, LJM, JPD, VC and LL. All the authors contributed to the interpretation of the output and revised and reviewed the paper; they are the guarantors. All authors contributed to the writing of the final manuscript and provided critical

For peer review only - http://bmjopen.bmj.com/site/about/guidelines.xhtml

comments during revisions. All authors approved the final version prior to submission. The Birmingham Clinical Trials Unit undertook the randomisation and data management and monitoring. The authors had full access to all the data from the study. The authors vouch for the accuracy and completeness of the data and analyses. All authors read and approved the final manuscript.

Data sharing statement

The dataset used in this manuscript is available on request from Birmingham Clinical Trials Unit.

peer review only

Table 1: Baseline Characteristics

**Characteristic**		**Oestrogen (n=50)**	**No Treatment (n=50)**	**Overall (n=100)**
Age, years[1]	<65	21 (42%)	21 (42%)	42 (42%)
	>=65	29 (58%)	29 (58%)	58 (58%)
	Mean (SD)	65.7 (8.2)	65.9 (8.4)	65.8 (8.3)
Ethnic Group	White	45 (90%)	43 (86%)	88 (88%)
	Asian	2 (4%)	3 (6%)	5 (5%)
	Black	3 (6%)	1 (2%)	4 (4%)
	Mixed	0 (-)	2 (4%)	2 (5%)
	Other ethnic group	0 (-)	1 (2%)	1 (1%)
BMI (mg/k^2)	Mean (SD)	28.1 (5.1)	28.2 (5.9)	28.2 (5.5)
	Missing (N)	2	7	9
Parity[1]	<=2	27 (54%)	26 (52%)	53 (53%)
	>2	23 (46%)	24 (48%)	47 (47%)
Maximum Stage of Prolapse[1]	I	7 (14%)	7 (14%)	14 (14%)
	II	23 (46%)	23 (46%)	46 (46%)
	III/IV	20 (40%)	20 (40%)	40 (40%)
Concomitant continence surgery performed[1]	N (%)	2 (4%)	3 (6%)	5 (5%)
Previous operation for prolapse	N (%)	4 (8%)	4 (8%)	8 (8%)
Anterior	No. of repairs Median (IQR)	1 (1 - 1)	1 (1 - 1)	1 (1 - 1)
Posterior	No. of repairs Median (IQR)	1 (1 - 1)	1 (1 - 1)	1 (1 - 1)
Hysterectomy	N (%)	12 (24%)	8 (16%)	20 (20%)
Vaginal pessary/ring currently in place	N (%)	5 (10%)	8 (16%)	13 (13%)
Physiotherapy treatment for prolapse/urinary incontinence in last 12 months	N (%)	8 (16%)	10 (20%)	18 (18%)

For peer review only - http://bmjopen.bmj.com/site/about/guidelines.xhtml

Drug treatment for urinary incontinence	N (%)	0 (-)	4 (8%)	4 (4%)
Treatment for overactive bladder	N (%)	0 (-)	0 (-)	0 (-)

[1] minimisation variable

For peer review only

For peer review only - http://bmjopen.bmj.com/site/about/guidelines.xhtml

Table 2: Operative details

**Aspect of surgery**		**Oestrogen (n=44)**	**No Treatment (n=45)**
POP surgery performed	Anterior repair	35 (80%)	34 (76%)
	Posterior repair	16 (36%)	19 (42%)
	Vaginal hysterectomy +/- bilateral salping-oopherectomy	26 (59%)	25 (56%)
	Sacrospinous Fixation	6 (14%)	6 (13%)
Concomitant surgery	Sling/Tension-free vaginal tape	1 (2%)	1 (2%)
	Botulinum toxin A	1 (2%)	1 (2%)
	Suprapublic catheter	1 (2%)	1 (2%)
Ease of dissection	Very easy	6 (14%)	5 (11%)
	Easy	22 (50%)	13 (29%)
	Normal	14 (%)	21 (47%)
	Difficult	2 (5%)	5 (11%)
	Very difficult	0 (-)	1 (2%)
Blood loss (g)	N; Median (IQR)	N=44<br>63 (50 - 125)	N=44<br>54 (50 - 100)
	Missing	0 (-)	1 (2%)
Visceral injury	Bladder injury	0 (-)	0 (-)
	Urethral injury	0 (-)	0 (-)
	Ureteric injury	1 (2%)	0 (-)
	Bowel injury	0 (-)	0 (-)
	Button holing of vagina	3 (7%)	1 (2%)
Suture used – Fascial plication[1]	Polyglactin (Vicryl®, Ethicon/Polysorb®, Medtronic)	10 (23%)	10 (22%)
	Polydioxanone (PDS®-II, Ethicon)	35 (80%)	34 (76%)
	Poligelcarprine (Monocryl®, Ethicon)	0 (-)	0 (-)
	Missing	0	1
Suture used – Vagina[1]	Polyglactin (Vicryl®, Ethicon/Polysorb®, Medtronic)	41 (93%)	42 (93%)
	Polydioxanone (PDS®-II, Ethicon)	5 (11%)	6 (13%)
	Poligelcarprine (Monocryl®, Ethicon)	3 (7%)	1 (2%)
	Vaginal packing used	23 (52%)	28 (62%)
Cather inserted in theatre	Yes	43 (98%)	42 (93%)
Post-operative complications noted before discharge	Infection	1 (2%)	3 (7%)
	Ureteric injury	0 (-)	0 (-)
	Bladder injury	0 (-)	0 (-)
	Bowel injury	0 (-)	0 (-)
	Vascular injury	0 (-)	0 (-)
	Neurological injury	0 (-)	0 (-)
	Peri or post-operative blood transfusion	0 (-)	0 (-)
	Peri or post-operative thromboembolism	0 (-)	0 (-)

For peer review only - http://bmjopen.bmj.com/site/about/guidelines.xhtml

Death	0 (-)	0 (-)

[1]More than one can be selected, hence why numbers do not add up to total.

For peer review only

For peer review only - http://bmjopen.bmj.com/site/about/guidelines.xhtml

Table 3: Results of patient reported outcomes – PFDI20, PFIQ-7 and PISQ-12

Outcome measure Timepoint	Oestrogen N; Mean (SD)	No Treatment N; Mean (SD)	Mean Difference between groups (95% CI)[3]
PFDI-SF20; POPDI-6 Domain (0-100, higher=worse pain)[1]			
Baseline	N=48; 43.4 (24.8)	N=49; 46.0 (24.4)	
6 Month	N=38; 16.3 (20.3)	N=41; 12.5 (11.6)	4.1 (-3.9, 12.1)
12 Month	N=37 16.7 (20.6)	N=41 14.6 (15.6)	4.4 (-4.7, 13.5)
PFDI-SF20; CRAD-8 Domain (0-100, higher=worse pain)[1]			
Baseline	N=48; 26.0 (25.0)	N=48; 24.3 (18.0)	
6 Month	N=38; 12.7 (14.4)	N=41; 15.2 (13.2)	-2.4 (-7.6, 3.4)
12 Month	N=37 12.8 (14.8)	N=42 15.8 (15.8)	-1.7 (-8.0, 4.6)
PFDI-SF20; UDI-6 Domain (0-100, higher=worse pain)[1]			
Baseline	N=48; 34.8 (22.6)	N=49; 37.9 (26.7)	
6 Month	N=38; 16.4 (17.5)	N=41; 17.2 (18.5)	0.5 (-8.9, 7.9)
12 Month	N=36 20.3 (17.5)	N=42 21.5 (25.3)	0.7 (-9.1, 10.4)
PFDI-SF20; Summary Score (0-300, higher=worse pain)[1]			
Baseline	N=48; 104.3 (62.8)	N=48; 107.0 (57.4)	
6 Month	N=38; 45.4 (43.8)	N=41; 45.0 (37.1)	0.6 (-18.4, 17.2)
12 Month	N=36 49.8 (44.1)	N=41 51.1 (47.7)	4.8 (-16.2, 25.8)
PFIQ-7; UIQ-7 Domain (0-100, higher=worse condition)[1]			
Baseline	N=50; 21.0 (25.8)	N=50; 21.5 (24.6)	
6 Month	N=38; 9.6 (21.7)	N=41; 5.9 (11.2)	1.3 (-6.9, 9.5)
12 Month	N=37; 8.9 (16.8)	N=42; 8.0 (13.9)	1.4 (-6.0, 8.8)
PFIQ-7; CRAIQ-7 Domain (0-100, higher=worse condition)[1]			
Baseline	N=50; 12.0 (21.0)	N=50; 8.7 (13.5)	
6 Month	N=38; 4.9 (13.1)	N=41; 2.4 (6.1)	1.6 (-2.5, 5.8)
12 Month	N=36; 4.5 (16.3)	N=42; 2.9 (6.1)	2.2 (-2.8, 7.1)
PFIQ-7; POPIQ-7 Domain (0-100, higher=worse condition)[1]			
Baseline	N=50;	N=50;	

For peer review only - http://bmjopen.bmj.com/site/about/guidelines.xhtml

	17.7 (21.2)	17.1 (22.1)	
6 Month	N=38; 5.0 (13.4)	N=41; 2.3 (6.1)	2.5 (-2.5, 7.5)
12 Month	N=36; 1.9 (6.8)	N=42; 1.7 (5.2)	0.2 (-2.7, 3.1)
PFIQ-7; Summary Score (0-300, higher=worse pain)[1]			
Baseline	N=50; 50.8 (54.8)	N=50; 47.3 (51.9)	
6 Month	N=38; 19.5 (39.8)	N=41; 10.7 (19.8)	6.2 (-8.7, 21.2)
12 Month	N=36; 14.3 (29.1)	N=42; 12.7 (18.3)	4.5 (-6.8, 15.8)
PISQ-12 (0-48, higher=better sexual function)[2]			
Baseline	N=18; 31.2 (6.5)	N=19; 33.1 (7.3)	
6 Month	N=11; 33.1 (4.1)	N=14; 36.1 (5.7)	-2.4 (-7.0, 2.2)
12 Month	N=11; 34.5 (2.7)	N=10; 32.8 (5.1)	3.4 (0.6, 6.2)

[1]<0 indicates less pain/better condition failures with oestrogen

[2]>0 indicates better with oestrogen

[3]Differences are adjusted for baseline score and minimisation variables

For peer review only

Table 4: Results of patient reported outcomes – patient global impression of improvement (PGI-I)

Timepoint<br>*PGI-I response*		Oestrogen	No Treatment	Relative risk (95%CI)[3]
**6 Week**		N=42	N=42	
1 = Very much better	N (%)	22 (52%)	14 (33%)	
2 = Much better	N (%)	16 (38%)	18 (43%)	
3 = A little better	N (%)	4 (10%)	8 (19%)	
4 = No change	N (%)	0 (-)	2 (5%)	
5 = A little worse	N (%)	0 (-)	0 (-)	
6 = Much Worse	N (%)	0 (-)	0 (-)	
7 = Very Much Worse	N (%)	0 (-)	0 (-)	
Better score[1]	N (%)	42 (100%)	40 (95%)	1.05 (0.98 – 1.12)
Worse score[2]	N (%)	0 (-)	2 (5%)	
**6 Month**		N=38	N=41	
1 = Very much better	N (%)	13 (34%)	18 (44%)	
2 = Much better	N (%)	16 (42%)	17 (41%)	
3 = A little better	N (%)	4 (11%)	5 (12%)	
4 = No change	N (%)	3 (8%)	0 (-)	
5 = A little worse	N (%)	2 (5%)	1 (2%)	
6 = Much Worse	N (%)	0 (-)	0 (-)	
7 = Very Much Worse	N (%)	0 (-)	0 (-)	
Better score[1]	N (%)	33 (87%)	40 (98%)	0.89 (0.78 – 1.02)
Worse score[2]	N (%)	5 (13%)	1 (2%)	
**12 Month**		N=37	N=42	
1 = Very much better	N (%)	14 (38%)	17 (40%)	
2 = Much better	N (%)	15 (41%)	17 (40%)	
3 = A little better	N (%)	3 (8%)	4 (10%)	
4 = No change	N (%)	4 (11%)	2 (5%)	
5 = A little worse	N (%)	1 (3%)	0 (-)	
6 = Much Worse	N (%)	0 (-)	2 (5%)	
7 = Very Much Worse	N (%)	0 (-)	0 (-)	
Better score[1]	N (%)	32 (86%)	38 (90%)	0.96 (0.81 – 1.12)
Worse score[2]	N (%)	5 (14%)	4 (10%)	

[1]Better score is derived from scores 1 (Very much better) to 3 (A little better)
[2]Worse score is derived from scores 4 (No change) to 7 (Very much worse)
[3]>1 indicates better with oestrogen

For peer review only

Table 5: Results of POP-Q assessment

Anatomical site in vagina Timepoint	**Oestrogen N; Mean (SD)**	**No Treatment N; Mean (SD)**	**Mean Difference between groups (95% CI)**
Aa (anterior wall), cm			
Baseline	N=49; 0.1 (1.9)	N=48; -0.3 (1.7)	
6 Months[1]	N=26; -1.7 (2.0)	N=29; -2.6 (2.0)	0.7 (0.1, 1.3)
Ba (anterior edge), cm			
Baseline	N=49; 0.2 (2.0)	N=48; 0.1 (1.8)	
6 Months[1]	N=25; -2.0 (2.0)	N=29; -2.9 (1.9)	0.6 (0.0, 1.2)
C (cervix/cuff), cm			
Baseline	N=49; -3.7 (2.5)	N=48; -3.4 (3.4)	
6 Months[1]	N=25; -2.9 (2.8)	N=29; -2.2 (3.6)	-0.2 (-1.3, 0.9)
Ap (posterior wall), cm			
Baseline	N=49; -1.2 (1.5)	N=48; -1.0 (1.7)	
6 Months[1]	N=26; -0.8 (1.7)	N=29; -1.0 (1.7)	0.5 (-0.2, 1.1)
Bp (posterior edge), cm			
Baseline	N=49; -1.1 (1.6)	N=48; -1.0 (1.8)	
6 Months[1]	N=26; -0.9 (1.8)	N=29; -1.0 (1.8)	0.4 (-0.3, 1.1)
D (posterior fornix), cm			
Baseline	N=39; -5.3 (1.9)	N=40; -5.1 (2.6)	
6 Months[1]	N=7; -1.3 (1.5)	N=4; -1.8 (1.9)	0.7 (-6.2, 7.5)
Total vaginal length, cm			
Baseline	N=49; 8.5 (1.1)	N=48; 8.0 (1.6)	
6 Months[2]	N=26; -0.2 (1.3)	N=29; -0.5 (1.7)	0.3 (0.4, 0.9)
Objective prolapse (any of Aa, Ba, C, Ap, Bp, or D>0) at six months			
	**N (%)**	**N (%)**	**Relative risk (95%CI)[3]**
Failure	9/26	13/29	0.77 (0.40 – 1.50)

[1]Change from baseline adjusted for baseline; difference <0 indicates better with oestrogen

[2]Adjusted for baseline length; difference<0 indicates better with oestrogen

[3]<1 indicates less prolapse failures with oestrogen

Figure1:Flow of participants through the trial

For peer review only - http://bmjopen.bmj.com/site/about/guidelines.xhtml

References

1.Haylen BT, MaherCF,Barber MD et al.An International Urogynecological Association (IUGA) / International Continence Society (ICS) joint report on the terminology for female pelvic organ prolapse (POP).*Int Urogynecol J.* 2016; 27 : 165-194

2. Nilsson K, Risberg B, Heimer G. The vaginal epithelium in the postmenopause--cytology, histology and pH as methods of assessment. Maturitas. 1995;21(1):51-6.
3.Ismail SI, Bain C, Hagen S. Oestrogens for treatment or prevention of pelvic organ prolapse in postmenopausal women. The Cochrane database of systematic reviews. 2010(9):CD007063.

4. Blatch-Jones AJ, Pek W, Kirkpatrick E, *et al.* Role of feasibility and pilot studies in randomised controlled trials: a cross-sectional study *BMJ Open* 2018;**8:**e022233. doi: 10.1136/bmjopen-2018-022233

5.Eldridge SM, Chan CL, Campbell MJ, Bond CM, Hopewell S, Thabane L, Lancaster GA; PAFS consensus group. CONSORT 2010 statement: extension to randomised pilot and feasibility trials. BMJ. 2016 Oct 24;355:i5239. PMID: 27777223

6.[Available from: https://www.birmingham.ac.uk/Documents/college-mds/trials/bctu/lotus/LOTUS-protocol-V2.0.pdf

7.Barber MD WM BR. Short forms of two condition-specific quality-of-life questionnaires for women with pelvic floor disorders (PFDI-20 and PFIQ-7). . Am J Obstet Gynecol 2005.

8. Rogers RG, Coates KW, Kammerer-Doak D, Khalsa S, Qualls C. A short form of the Pelvic Organ Prolapse/Urinary Incontinence Sexual Questionnaire (PISQ-12). Int Urogynecol J Pelvic Floor Dysfunct. 2003;14(3):164-8.

9. Srikrishna S, Robinson D, Cardozo L. Validation of the Patient Global Impression of Improvement (PGI-I) for urogenital prolapse. Int Urogynecol J. 2010;21(5):523-8.

10. Felding C, Mikkelsen AL, Clausen HV, Loft A, Larsen LG. Preoperative treatment with oestradiol in women scheduled for vaginal operation for genital prolapse. A randomised, double-blind trial. Maturitas. 1992;15(3):241-9.

11. Mikkelsen AL, Felding C, Clausen HV. Clinical effects of preoperative oestradiol treatment before vaginal repair operation. A double-blind, randomized trial. Gynecol Obstet Invest. 1995;40(2):125-8.

12. Vaccaro CM, Mutema GK, Fellner AN, Crisp CC, Estanol MV, Kleeman SD, et al. Histologic and cytologic effects of vaginal estrogen in women with pelvic organ prolapse: a randomized controlled trial. Female Pelvic Med Reconstr Surg. 2013;19(1):34-9.

13. Moalli PA, Shand SH, Zyczynski HM, Gordy SC, Meyn LA. Remodeling of vaginal connective tissue in patients with prolapse. Obstet Gynecol. 2005;106(5 Pt 1):953-63.

For peer review only

14. Karp DR, Jean-Michel M, Johnston Y, Suciu G, Aguilar VC, Davila GW. A randomized clinical trial of the impact of local estrogen on postoperative tissue quality after vaginal reconstructive surgery. Female Pelvic Med Reconstr Surg. 2012 Jul-Aug;18(4):211-5.

15. Glazener CM, Breeman S, Elders A, Hemming C, Cooper KG, Freeman RM, et al. Mesh, graft, or standard repair for women having primary transvaginal anterior or posterior compartment prolapse surgery: two parallel-group, multicentre, randomised, controlled trials (PROSPECT). Lancet. 2017;389(10067):381-92.

For peer review only

For peer review only - http://bmjopen.bmj.com/site/about/guidelines.xhtml

Figure 1: Flow of participants through the trial

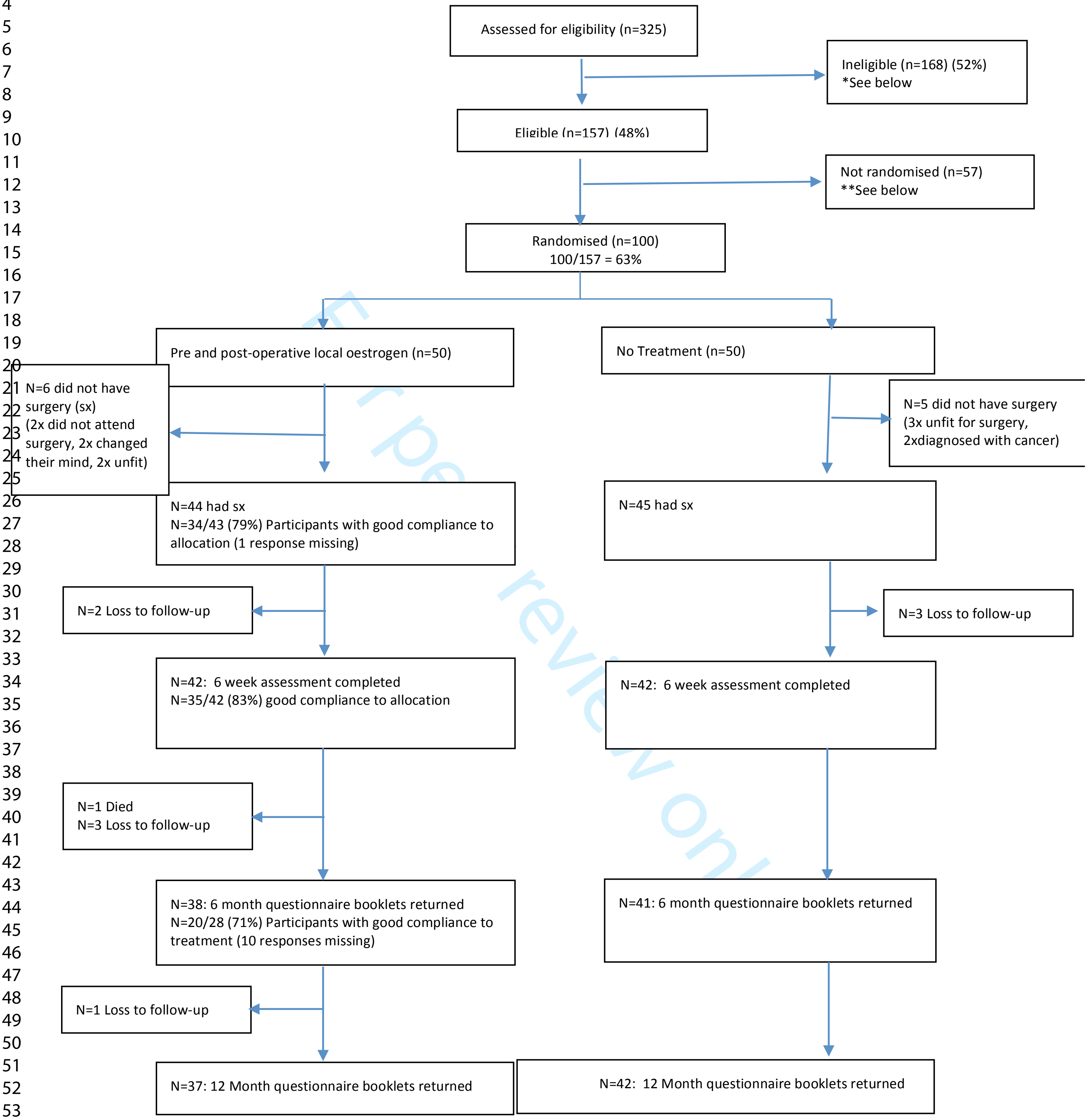

• *Reasons for Ineligibility N=168 (%): No prolapse surgery required 38 (22), Recurrence of prolapse in the same compartment 32(19), Has had previous breast or uterine malignancy 20 (12), Undergoing HRT treatment 17 (10), Unable to understand English 16 (9), Genital bleeding of unknown origin 15 (9), Has had previous hormone-dependent neoplasms 13 (8), Previous POP surgery involving mesh 6 (3), Previous thrombo-embolic episode related to oestrogen therapy 4 (2), Two or more culture positive UTI episodes in the last 6 months 1 (<1), Other 6 (3)

**Reasons for Non Randomisation N=57 (%):Patient did not attend clinic 19 (33), Not enough time to discuss trial and gain informed consent 14 (25), Declined Consent/not willing to be randomised 9 (16), Not willing to wait 6 weeks for surgery 5 (9), Unable to gain consent 4 (7), Did not want to complete questionnaires, 1(2)

For peer review only - http://bmjopen.bmj.com/site/about/guidelines.xhtml

For peer review only

CONSORT 2010 checklist of information to include when reporting a randomised trial*

Section/Topic	Item No	Checklist item	Reported on page No
**Title and abstract**			
	1a	Identification as a randomised trial in the title	1
	1b	Structured summary of trial design, methods, results, and conclusions (for specific guidance see CONSORT for abstracts)	2
**Introduction**			
Background and objectives	2a	Scientific background and explanation of rationale	5
	2b	Specific objectives or hypotheses	5
**Methods**			
Trial design	3a	Description of trial design (such as parallel, factorial) including allocation ratio	6
	3b	Important changes to methods after trial commencement (such as eligibility criteria), with reasons	7
Participants	4a	Eligibility criteria for participants	7
	4b	Settings and locations where the data were collected	7
Interventions	5	The interventions for each group with sufficient details to allow replication, including how and when they were actually administered	8
Outcomes	6a	Completely defined pre-specified primary and secondary outcome measures, including how and when they were assessed	8
	6b	Any changes to trial outcomes after the trial commenced, with reasons	8
Sample size	7a	How sample size was determined	9
	7b	When applicable, explanation of any interim analyses and stopping guidelines	9
Randomisation:			
Sequence generation	8a	Method used to generate the random allocation sequence	7
	8b	Type of randomisation; details of any restriction (such as blocking and block size)	7
Allocation concealment mechanism	9	Mechanism used to implement the random allocation sequence (such as sequentially numbered containers), describing any steps taken to conceal the sequence until interventions were assigned	7
Implementation	10	Who generated the random allocation sequence, who enrolled participants, and who assigned participants to interventions	7
Blinding	11a	If done, who was blinded after assignment to interventions (for example, participants, care providers, those	-

For peer review only

For peer review only - http://bmjopen.bmj.com/site/about/guidelines.xhtml

		assessing outcomes) and how	
	11b	If relevant, description of the similarity of interventions	-
Statistical methods	12a	Statistical methods used to compare groups for primary and secondary outcomes	9
	12b	Methods for additional analyses, such as subgroup analyses and adjusted analyses	9
**Results**			
Participant flow (a diagram is strongly recommended)	13a	For each group, the numbers of participants who were randomly assigned, received intended treatment, and were analysed for the primary outcome	10
	13b	For each group, losses and exclusions after randomisation, together with reasons	11
Recruitment	14a	Dates defining the periods of recruitment and follow-up	11
	14b	Why the trial ended or was stopped	
Baseline data	15	A table showing baseline demographic and clinical characteristics for each group	10
Numbers analysed	16	For each group, number of participants (denominator) included in each analysis and whether the analysis was by original assigned groups	10-11
Outcomes and estimation	17a	For each primary and secondary outcome, results for each group, and the estimated effect size and its precision (such as 95% confidence interval)	9-11
	17b	For binary outcomes, presentation of both absolute and relative effect sizes is recommended	10-11
Ancillary analyses	18	Results of any other analyses performed, including subgroup analyses and adjusted analyses, distinguishing pre-specified from exploratory	
Harms	19	All important harms or unintended effects in each group (for specific guidance see CONSORT for harms)	11
**Discussion**			
Limitations	20	Trial limitations, addressing sources of potential bias, imprecision, and, if relevant, multiplicity of analyses	14
Generalisability	21	Generalisability (external validity, applicability) of the trial findings	12-14
Interpretation	22	Interpretation consistent with results, balancing benefits and harms, and considering other relevant evidence	12-14
**Other information**			
Registration	23	Registration number and name of trial registry	1-2
Protocol	24	Where the full trial protocol can be accessed, if available	7
Funding	25	Sources of funding and other support (such as supply of drugs), role of funders	15

*We strongly recommend reading this statement in conjunction with the CONSORT 2010 Explanation and Elaboration for important clarifications on all the items. If relevant, we also recommend reading CONSORT extensions for cluster randomised trials, non-inferiority and equivalence trials, non-pharmacological treatments, herbal interventions, and pragmatic trials. Additional extensions are forthcoming: for those and for up to date references relevant to this checklist, see www.consort-statement.org.

For peer review only
